# A cross-national study of factors associated with women’s perinatal mental health and wellbeing during the COVID-19 pandemic

**DOI:** 10.1371/journal.pone.0249780

**Published:** 2021-04-21

**Authors:** Archana Basu, Hannah H. Kim, Rebecca Basaldua, Karmel W. Choi, Lily Charron, Nora Kelsall, Sonia Hernandez-Diaz, Diego F. Wyszynski, Karestan C. Koenen

**Affiliations:** 1 Harvard T.H. Chan School of Public Health, Boston, Massachusetts, United States of America; 2 Massachusetts General Hospital, Boston, Massachusetts, United States of America; 3 Hamilton College, Clinton, New York, United States of America; 4 Pregistry, Los Angeles, California, United States of America; Erasmus Medical Center, NETHERLANDS

## Abstract

Pregnant and postpartum women face unique challenges during the COVID-19 pandemic that may put them at elevated risk of mental health problems. However, few large-scale and no cross-national studies have been conducted to date that investigate modifiable pandemic-related behavioral or cognitive factors that may influence mental health in this vulnerable group. This international study sought to identify and measure the associations between pandemic-related information seeking, worries, and prevention behaviors on perinatal mental health during the COVID-19 pandemic. An anonymous, online, cross-sectional survey of pregnant and postpartum women was conducted in 64 countries between May 26, 2020 and June 13, 2020. The survey, available in twelve languages, was hosted on the Pregistry platform for COVID-19 studies (https://corona.pregistry.com) and advertised in social media channels and online parenting forums. Participants completed measures on demographics, COVID-19 exposure and worries, information seeking, COVID-19 prevention behaviors, and mental health symptoms including posttraumatic stress via the IES-6, anxiety/depression via the PHQ-4, and loneliness via the UCLA-3. Of the 6,894 participants, substantial proportions of women scored at or above the cut-offs for elevated posttraumatic stress (2,979 [43%]), anxiety/depression (2,138 [31%], and loneliness (3,691 [53%]). Information seeking from any source (e.g., social media, news, talking to others) five or more times per day was associated with more than twice the odds of elevated posttraumatic stress and anxiety/depression, in adjusted models. A majority of women (86%) reported being somewhat or very worried about COVID-19. The most commonly reported worries were related to pregnancy and delivery, including family being unable to visit after delivery (59%), the baby contracting COVID-19 (59%), lack of a support person during delivery (55%), and COVID-19 causing changes to the delivery plan (41%). Greater worries related to children (i.e., inadequate childcare, their infection risk) and missing medical appointments were associated with significantly higher odds of posttraumatic stress, anxiety/depression and loneliness. Engaging in hygiene-related COVID-19 prevention behaviors (face mask-wearing, washing hands, disinfecting surfaces) were not related to mental health symptoms or loneliness. Elevated posttraumatic stress, anxiety/depression, and loneliness are highly prevalent in pregnant and postpartum women across 64 countries during the COVID-19 pandemic. Excessive information seeking and worries related to children and medical care are associated with elevated symptoms, whereas engaging in hygiene-related preventive measures were not. In addition to screening and monitoring mental health symptoms, addressing excessive information seeking and women’s worries about access to medical care and their children’s well-being, and developing strategies to target loneliness (e.g., online support groups) should be part of intervention efforts for perinatal women. Public health campaigns and medical care systems need to explicitly address the impact of COVID-19 related stressors on mental health in perinatal women, as prevention of viral exposure itself does not mitigate the pandemic’s mental health impact.

## Introduction

The United Nations Children’s Fund estimates that there have been 116 million births in the ten months since COVID-19 was declared a pandemic by the World Health Organization (WHO) on March 11 2020 [1, 2]. The unique challenges facing pregnant and postpartum women include concerns about greater severity of COVID-19 disease in this population, potential vertical transmission from an infected mother to her newborn, and increased risk of adverse neonatal outcomes [3, 4]. Recent studies also suggest elevated rates of mental health problems among perinatal women during the pandemic including depression [5–10], anxiety [5–13], dissociation [5], and posttraumatic stress symptoms [5, 13], as well as loneliness and isolation [14] as compared to perinatal women before the pandemic. Perinatal women who have a history of psychological disorders, are experiencing higher concerns regarding their family’s health, their baby’s future and society due to the COVID-19 pandemic [15]. Meanwhile, obstetricians report that perinatal women are contacting them expressing concern about hospital visits, protection methods, their infant’s safety, anxieties due to social media and fears of contracting COVID-19 [16]. Perinatal mental health problems are a critical public health issue as such problems adversely impact women’s own health, infant outcomes [17], mother-infant bonding, and later offspring physical and behavioral health [18–20]. Thus, identifying the factors that influence mental health of pregnant and postpartum women during the COVID-19 pandemic is critical for both their own well-being and that of future generations.

To date, there have been no large-scale cross-national studies of women’s perinatal mental health during the COVID-19 pandemic. Moreover, previous studies have not assessed loneliness as a mental health experience in this vulnerable group, despite the well-established importance of social connection during the perinatal period [21] and known adverse effects of loneliness on mental health, morbidity and mortality [22]. Recent work has also examined only a narrow range of behavioral and cognitive factors associated with mental health outcomes in this vulnerable group. In terms of cognitive factors such as worries and concerns, the COVID-19 pandemic has radically altered the perinatal experience as healthcare services broadly shift from in person to online and as support persons are restricted at appointments and delivery. In addition to the concerns about one’s own risk of contracting COVID-19, pregnant or postpartum women are faced with the possible effects of infection on the developing fetus or infant. Economic uncertainty coupled with increased isolation may also negatively affect maternal mental health. Finally, little is known about pregnant and postpartum women’s engagement in behaviors aimed at understanding the pandemic (e.g., information seeking) and preventing COVID-19 infection, and how such behaviors may or may not be associated with their mental health.

We conducted a cross-national survey of pregnant and postpartum women to document the prevalence of posttraumatic stress, anxiety, and depression symptoms as well as loneliness in this population. We then examined the relation of type and amount of information seeking via various channels (social media, news, discussion with others) to women’s mental health and loneliness. Next, we analyzed the role of pandemic-related worries in mental health and loneliness. Finally, we investigated the association between engaging in COVID-19 prevention behaviors and mental health and loneliness. The primary goal of the study was to identify modifiable behavioral and cognitive factors, which if addressed, may reduce mental health risk and improve well-being among pregnant and postpartum women.

## Materials and methods

### Study design and setting

An anonymous, online, cross-sectional survey targeting pregnant and postpartum women was conducted in 64 countries between May 26, 2020 and June 13, 2020. Participation in this study was voluntary. All potential participants were informed about the research objectives and standards of confidentiality regarding the use of the data. The survey, hosted on the Pregistry platform for COVID-19 studies (https://corona.pregistry.com), was advertised predominantly in social media channels and online parenting forums. Advertisements and the survey were available in twelve languages (Arabic, Chinese, English, French, German, Italian, Korean, Portuguese, Russian, Spanish, Turkish, and Urdu) by human translators. The study sought to obtain at least 100 responses from each of the countries with the highest number of COVID-19 cases at the time of recruitment. Interested participants were invited to follow a link to take the survey. The survey collected standard demographic data and included questions that addressed topics such as COVID-19 exposure and worries, lifestyle changes, information seeking, protective factors, and mental health. The survey (including all translations) can be found in S1 Text and the informed consent is displayed in S1A Fig.

### Participants

Women who self-identified as being 18 years or older at the time of the survey and as currently pregnant or having given birth within the past 6 months were eligible to participate. The study was classified exempt by the Harvard Longwood Campus Institutional Review Board (HLC IRB) per the regulations found at 45 CFR 46.104(d)(2) on the basis that it poses no greater than minimal risk and that the recorded information cannot readily identify the subject (directly or indirectly). The total number of participants at the close of enrollment was 7,562 individuals across 64 countries.

### COVID-19 assessments

#### COVID-19 exposure

Questions assessing whether participants were tested, diagnosed, or in contact with an individual who had COVID-19 were adapted for this study based on those formulated by the US Centers for Disease Control and Prevention for the Household Pulse Survey [23].

#### COVID-19 information seeking

Participants were asked about the frequency (never, <1x/day, 2-4x/day, 5-8x/day, 9-16x/day, and >16x/day) of their interactions with various sources of information, including the news, social media, and interpersonal discussions about COVID-19 using a measure modified from other published studies [24, 25]. For analyses, interactions were categorized as never, <1x/day, 2-4x/day, 5+x/day.

#### COVID-19 worries

Participants were asked to rate their overall level of worry about COVID-19 on a Likert Scale ranging from 1 for “not worried at all” to 4 for “very worried” [26]. They were then asked to endorse fifteen specific worries on a list developed for this study. Exploratory factor analyses were conducted to identify domains within the questionnaire, with oblimin rotation on a tetrachoric correlation matrix due to the binary nature of the variables. Details of the factor analyses are presented in the S2 Table. Worries were categorized into the following domains: social (parents/grandparents unable to visit, family unable to visit, not able to have a baby shower, not able to attend a funeral), COVID-19 infection-related (participant or partner will bring infection home, family or friends will get COVID-19), child-related (no adequate childcare, other children will get COVID-19), delivery-related (partner not present during delivery, changes to delivery plan, unborn baby will get COVID-19, not able to breastfeed), economic (significantly affect economic situation/finances), and missing doctor appointments.

#### COVID-19 prevention behaviors

Participants were asked to endorse seventeen behaviors they had engaged in to protect themselves from COVID-19 from a list developed for this study based on WHO recommendations and media reports. Behaviors were classified into the following categories: hygiene-related (mask-wearing, washing hands, disinfecting surfaces), physical distancing (avoiding public places, restaurants and other people, canceling personal engagements, work or school and working at home), canceling travel (for work or pleasure), stockpiling essential resources (food or water, hand sanitizer, medication), postponing medical care, and prayer.

### Mental health outcomes

#### Depression and anxiety assessment

Depression and anxiety was assessed via the Patient Health Questionnaire-4 (PHQ-4) [27, 28], a four-item inventory rated on a four-point Likert-type scale. Items are drawn from the first two items of the ’Generalized Anxiety Disorder–7 scale’ (GAD–7) and the ’Patient Health Questionnaire-8’ (PHQ-8). The overall PHQ-4 score is a sum of the four items (0 = not at all, 1 = several days, 2 = more than half the days, 3 = nearly every day). A PHQ-4 score of > = 6 is considered positive screen for a possible depressive or anxiety disorder. For the purpose of this analyses, we consider a PHQ-4 score of > = 6 to indicate “elevated” depression or anxiety disorder symptoms.

#### Posttraumatic stress symptom assessment

Posttraumatic stress symptoms were assessed via a modified version of the Impact of Events Scale—6 (IES-6). Participants were asked to report how bothered they were by each symptom from 0 (Not at all) to 4 (Extremely) over the past seven days. The symptom statements were modified to assess impact related to the COVID-19 pandemic. The scale is scored by calculating the mean of the five items used in this study, with the original IES-6 cutoff score of 1.75 yielding 0.88 sensitivity and 0.85 specificity for posttraumatic stress disorder (PTSD) diagnosis [29–31].

#### Loneliness assessment

Loneliness was evaluated using the UCLA Three-Item Loneliness Scale (UCLA-3) [32]. Participants are asked how often they experience the following: feeling that they lack companionship, feeling left out, and feeling isolated from others. Questions were framed so that participants described their feelings since the start of the COVID-19 pandemic. Responses are rated as 1 (Hardly Ever), 2 (Some of the Time), or 3 (Often), with the overall score as the sum of the three responses. A score of 3 is considered low, 4–5 medium, and > = 6 high. High loneliness has been associated with poorer mental and physical health over the life course [33].

### Sociodemographic factors and potential confounders

Standard socio-demographic measures were collected including age, education (categorized as never attended school, elementary school, some high school, high school graduate or general equivalency diploma (GED), some college/university, college diploma or university degree, master’s degree, professional degree, doctoral degree), self-identified race/ethnicity (categorized as White/Caucasian, Latina/Hispanic, Asian, South Asian, Black, Middle Eastern, Native Hawaiian or Other, Pacific Islander, American Indian or Alaska Native, Other/Multiracial), employment status (healthcare worker in a hospital or clinic, worked in a nursing home, essential/key worker (as defined by the government), none of these, don’t know), medical coverage status, marital status (married, living with partner, divorced, separated, single, widowed), weeks pregnant/postpartum (first trimester 0 to <13 weeks, second trimester 13 to <28 weeks, third trimester 28+ weeks, postpartum). Participants indicated their country of residence which was classified by region for analytic purposes.

### Statistical analyses

Descriptive statistics for socio-demographic characteristics, COVID-19 exposure variables, mental health, and loneliness were calculated by perinatal stage, classified as first, second, or third trimester, or postpartum, for all participants. A series of multivariable logistic regressions were then run to test the study hypotheses. For these analyses, PHQ-4, IES-6 and UCLA-3 outcomes were specified as binary dependent variables using recommended cutoffs. The first set of models included pregnancy stage, socio-demographic variables and COVID-19 exposure as independent variables. The second set of models examined the type and amount of information seeking about COVID-19 as independent variables. The third and fourth set of models focused on COVID-19 worries and COVID-19 behaviors as independent variables. Models for information seeking, COVID-19 worries, and COVID-19 behaviors were adjusted for age, level of education, race, survey region, marital status, and pregnancy stage.

Models were estimated using the GLM function in the R statistical program, version 3.6.2. Due to the number of models run, interpretations are focused on effects that met the threshold of p < .001. Due to low levels of missingness (667 [9%]) a complete case analysis was conducted.

## Results

### Study population

**Fig 1:**
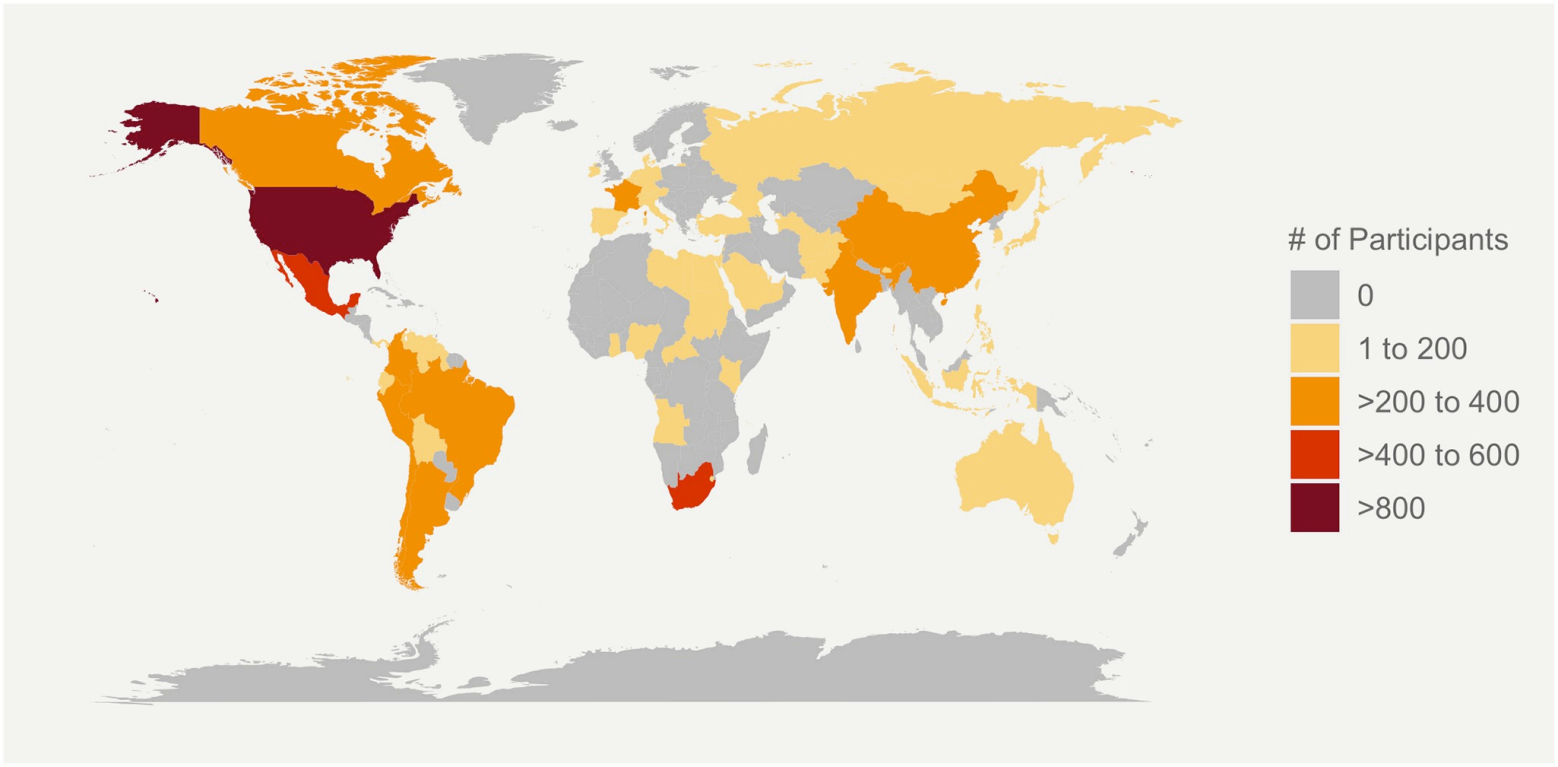
Geographic distribution of study participants by country^a^. ^a^The world map was generated using open-source data in the R statistical package “rworldmap” (https://cran.r-project.org/package=rworldmap).

The final analytic population consisted of 6,894 women residing in 64 countries ranging in age from 18 to 46 years. Due to the convenience sampling design, information on response rate is not available. Fig 1 visually displays the geographic distribution of study participants globally.

Demographic characteristics are reported in Table 1. The majority of women reported not having been tested for COVID-19 (89%), not having been in contact with an individual who has or had COVID-19 (78%), never having been diagnosed with COVID-19 (98%), and not being an essential or healthcare worker (75%).

**Table 1:** Descriptive statistics for survey respondents in the analytic sample, overall and by pregnancy status.

	Gestational weeks at participation				
	0 to <13 weeks	13 to <28 weeks	28+ weeks	Postpartum	Overall
Variable	(n = 1,276)	(n = 2,443)	(n = 1,993)	(n = 1,182)	(n = 6,894)
**Age in years**					
Mean (SD)	30.2 (4.92)	31.4 (4.71)	31.7 (4.77)	31.7 (4.94)	31.3 (4.84)
Median [Min, Max]	30.0 [18.0, 44.0]	31.0 [18.0, 44.0]	32.0 [18.0, 46.0]	32.0 [18.0, 46.0]	32.0 [18.0, 46.0]
**Region**					
Africa	137 (10.7%)	284 (11.6%)	204 (10.2%)	90 (7.6%)	715 (10.4%)
Asia & Pacific	333 (26.1%)	306 (12.5%)	206 (10.3%)	320 (27.1%)	1,165 (16.9%)
Europe	186 (14.6%)	626 (25.6%)	517 (25.9%)	304 (25.7%)	1,633 (23.7%)
Middle East	18 (1.4%)	48 (2.0%)	29 (1.5%)	16 (1.4%)	111 (1.6%)
North America	194 (15.2%)	368 (15.1%)	405 (20.3%)	278 (23.5%)	1,245 (18.1%)
South/Latin America	408 (32.0%)	811 (33.2%)	632 (31.7%)	174 (14.7%)	2,025 (29.4%)
**Race/ethnicity**					
White	464 (36.4%)	1,134 (46.4%)	995 (49.9%)	582 (49.2%)	3,175 (46.1%)
Latin/Hispanic	285 (22.3%)	565 (23.1%)	438 (22.0%)	131 (11.1%)	1,419 (20.6%)
Asian	261 (20.5%)	223 (9.1%)	152 (7.6%)	262 (22.2%)	898 (13.0%)
Black	123 (9.6%)	215 (8.8%)	169 (8.5%)	74 (6.3%)	581 (8.4%)
South Asian	28 (2.2%)	42 (1.7%)	37 (1.9%)	21 (1.8%)	128 (1.9%)
Middle Eastern	11 (0.9%)	34 (1.4%)	27 (1.4%)	17 (1.4%)	89 (1.3%)
Native/Indigenous	7 (0.5%)	7 (0.3%)	6 (0.3%)	4 (0.3%)	24 (0.3%)
More than 1 race/ethnicity	45 (3.5%)	108 (4.4%)	95 (4.8%)	45 (3.8%)	293 (4.3%)
Other	43 (3.4%)	83 (3.4%)	58 (2.9%)	33 (2.8%)	217 (3.1%)
Not reported	9 (0.7%)	32 (1.3%)	16 (0.8%)	13 (1.1%)	70 (1.0%)
**Marital status**					
Married	811 (63.6%)	1,518 (62.1%)	1,292 (64.8%)	852 (72.1%)	4,473 (64.9%)
Living with partner	355 (27.8%)	725 (29.7%)	507 (25.4%)	249 (21.1%)	1,836 (26.6%)
Other	110 (8.6%)	200 (8.2%)	194 (9.7%)	81 (6.9%)	585 (8.5%)
**Level of education**					
High school graduate or less	164 (12.9%)	335 (13.7%)	281 (14.1%)	142 (12.0%)	922 (13.4%)
Some college	205 (16.1%)	363 (14.9%)	280 (14.0%)	122 (10.3%)	970 (14.1%)
College graduate	462 (36.2%)	871 (35.7%)	720 (36.1%)	511 (43.2%)	2,564 (37.2%)
Graduate school or more	445 (34.9%)	874 (35.8%)	712 (35.7%)	407 (34.4%)	2,438 (35.4%)
**Employment status**					
Worked in healthcare or nursing home	144 (11.3%)	301 (12.3%)	225 (11.3%)	120 (10.2%)	790 (11.5%)
Essential worker	189 (14.8%)	378 (15.5%)	273 (13.7%)	113 (9.6%)	953 (13.8%)
Other	943 (73.9%)	1,764 (72.2%)	1,495 (75.0%)	949 (80.3%)	5,151 (74.7%)
**Medical coverage status**					
Yes	885 (69.4%)	1,699 (69.5%)	1,442 (72.4%)	873 (73.9%)	4,899 (71.1%)
**Tested for COVID-19**					
Negative, I did not have the virus	130 (10.2%)	139 (5.7%)	134 (6.7%)	238 (20.1%)	641 (9.3%)
No, I have not been tested	1,114 (87.3%)	2,260 (92.5%)	1,824 (91.5%)	913 (77.2%)	6,111 (88.6%)
Positive, I had the virus	14 (1.1%)	23 (0.9%)	17 (0.9%)	8 (0.7%)	62 (0.9%)
Yes, but I do not know the result yet or the result was inconclusive	18 (1.4%)	21 (0.9%)	18 (0.9%)	23 (1.9%)	80 (1.2%)
**In contact with an individual who has COVID-19**					
No	972 (76.2%)	1,887 (77.2%)	1,564 (78.5%)	943 (79.8%)	5,366 (77.8%)
Maybe	198 (15.5%)	371 (15.2%)	287 (14.4%)	162 (13.7%)	1,018 (14.8%)
Yes	106 (8.3%)	185 (7.6%)	142 (7.1%)	77 (6.5%)	510 (7.4%)
**Diagnosed with COVID-19**					
No	1,246 (97.6%)	2,394 (98.0%)	1,971 (98.9%)	1,166 (98.6%)	6,777 (98.3%)
Yes, and I still have it	12 (0.9%)	12 (0.5%)	5 (0.3%)	3 (0.3%)	32 (0.5%)
Yes, but I recovered	18 (1.4%)	37 (1.5%)	17 (0.9%)	13 (1.1%)	85 (1.2%)
**IES-6 score**					
<1.75	751 (58.9%)	1,367 (56.0%)	1,149 (57.7%)	648 (54.8%)	3,915 (56.8%)
> = 1.75	525 (41.1%)	1,076 (44.0%)	844 (42.3%)	534 (45.2%)	2,979 (43.2%)
**PHQ4 score**					
<6	850 (66.6%)	1,702 (69.7%)	1,385 (69.5%)	819 (69.3%)	4,756 (69.0%)
> = 6	426 (33.4%)	741 (30.3%)	608 (30.5%)	363 (30.7%)	2,138 (31.0%)
**UCLA-3 score**					
<6	646 (50.6%)	1,146 (46.9%)	888 (44.6%)	523 (44.2%)	3,203 (46.5%)
> = 6	630 (49.4%)	1,297 (53.1%)	1,105 (55.4%)	659 (55.8%)	3,691 (53.5%)

Abbreviations: SD, standard deviation.

### Mental health and loneliness

**Fig 2:**
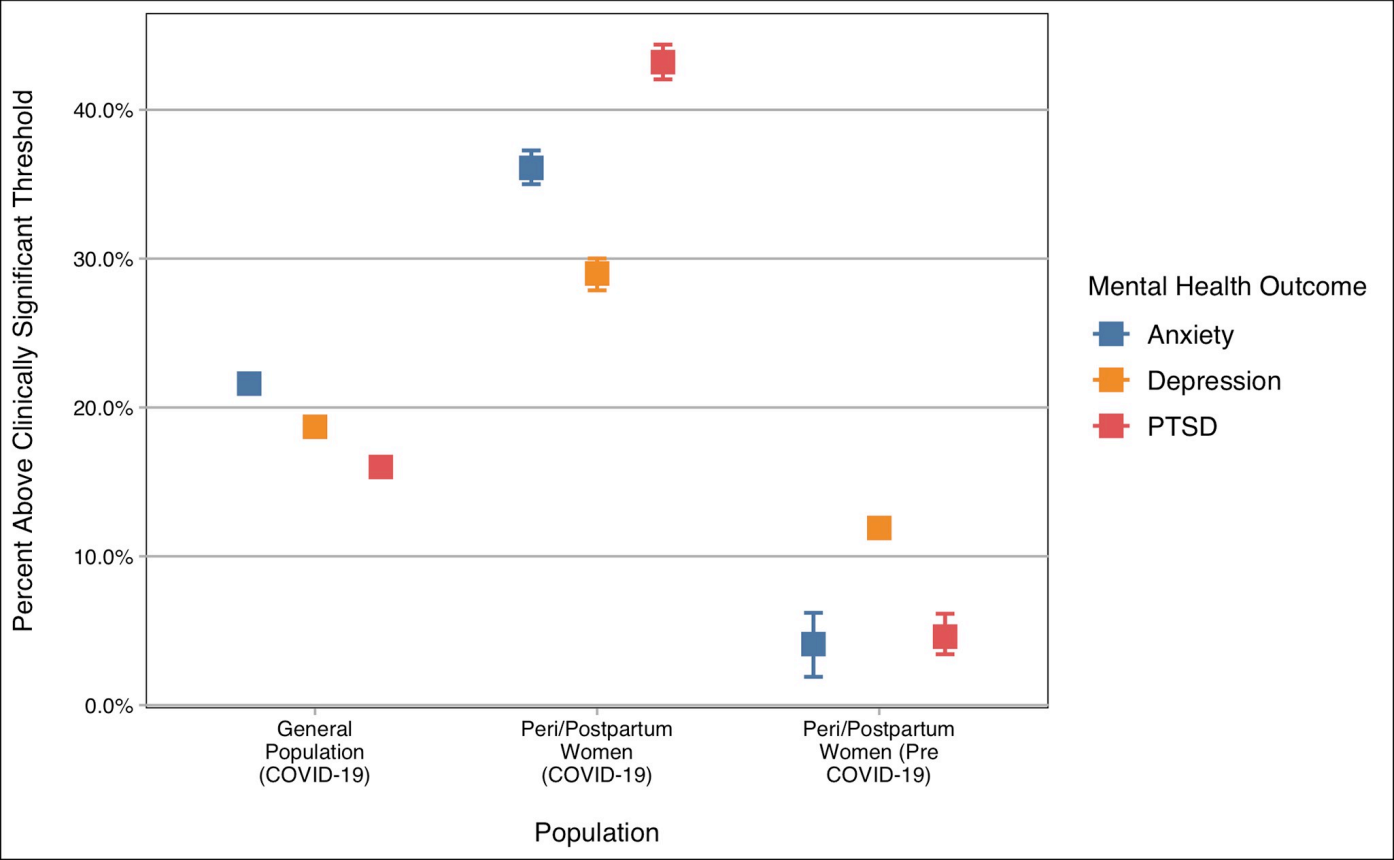
Comparison of anxiety, depression and posttraumatic stress prevalence between the general population and peri/postpartum women during the COVID-19 pandemic, and peri/postpartum women prior to the pandemic.

Substantial proportions of women scored at or above the risk cut-offs for IES-6 (2,979 [43%]), PHQ-4 (2,138 [31%]), and UCLA-3 (3,691 [54%]). Fig 2 presents the proportions of women in our sample with elevated PTSD, anxiety and depression symptoms compared with previously published studies of perinatal depression [18], anxiety [19], and PTSD [34] prior to the COVID-19 pandemic and U.S. general population studies of mental health during COVID-19 [35, 36].

Results for multivariable models of socio-demographic and COVID-19 exposure variables in relation to IES-6, PHQ4 and UCLA-3 are presented in S1A Table. In fully adjusted models, only age was consistently associated with reduced risk of significantly elevated outcomes across measures at p < .001. Only “maybe” having been in contact with someone with COVID-19 compared to “never” was consistently associated with increased risk of mental health symptoms across measures at p < .001.

### Information seeking and mental health

Table 2 presents the results of multivariable logistic regression models for specific types of information seeking in relation to IES-6, PHQ4 and UCLA-3.

**Table 2:** Model results for elevated symptoms of PTSD, depression/anxiety, and loneliness in relation to frequency of different types of information seeking^a^.

	N (% of total pop.)	PTSD (IES-6)	Depression/Anxiety (PHQ4)	Loneliness (UCLA-3)
		*OR (95% CI)*	*OR (95% CI)*	*OR (95% CI)*
**Check news**				
<1 time a day	2459 (35.7%)	*Reference*	*Reference*	*Reference*
1 time a day	2254 (32.7%)	1.16 (1.03–1.30)	1.13 (0.99–1.29)	1.02 (0.91–1.14)
2–4 times a day	1643 (23.8%)	1.54 (1.35–1.76)*	1.72 (1.50–1.98)*	1.11 (0.97–1.26)
5+ times a day	538 (7.8%)	2.15 (1.77–2.61)*	2.55 (2.09–3.11)*	1.43 (1.18–1.74)*
**Check social media**				
<1 time a day	2843 (41.2%)	Reference	Reference	Reference
1 time a day	1571 (22.8%)	1.18 (1.04–1.34)	1.20 (1.04–1.39)	1.09 (0.96–1.23)
2–4 times a day	1651 (23.9%)	1.48 (1.30–1.68)*	1.75 (1.53–2.01)*	1.35 (1.19–1.53)*
5+ times a day	829 (12.0%)	2.25 (1.92–2.65)*	2.83 (2.39–3.34)*	1.38 (1.17–1.62)*
**Discuss mass communications**				
<1 time a day	4664 (67.7%)	Reference	Reference	Reference
1 time a day	1048 (15.2%)	1.21 (1.05–1.38)	1.30 (1.12–1.51)*	1.23 (1.07–1.42)
2–4 times a day	844 (12.2%)	1.60 (1.38–1.86)*	1.86 (1.59–2.18)*	1.06 (0.91–1.23)
5+ times a day	338 (4.9%)	2.19 (1.74–2.77)*	2.73 (2.16–3.44)*	1.48 (1.17–1.86)*
**Discuss with others**				
<1 time a day	2706 (39.3%)	Reference	Reference	Reference
1 time a day	1787 (25.9%)	1.29 (1.14–1.46)*	1.16 (1.01–1.33)	1.07 (0.95–1.21)
2–4 times a day	1825 (26.5%)	1.66 (1.46–1.88)*	1.64 (1.43–1.88)*	1.24 (1.09–1.40)*
5+ times a day	576 (8.4%)	2.35 (1.95–2.84)*	2.65 (2.19–3.22)*	1.26 (1.04–1.52)
**Number of types of information seeking 5+ times a day**	mean (SD) 0.33 (0.79)	1.37 (1.28–1.46)*	1.47 (1.38–1.57)*	1.13 (1.06–1.20)*

*p<0.001.

^a^ All models were adjusted for age, education, race/ethnicity, medical coverage status, survey region, marital status, weeks pregnant/postpartum.

Abbreviations: OR, odds ratios; pop., population; SD, standard deviation.

For all types of information seeking, there was a dose-response relation between information seeking frequency and meeting the threshold for both the IES-6 and PHQ-4, in fully adjusted models. In fact, participants engaging in any type of information seeking five or more times per day had more than twice the odds of meeting the elevated threshold for the IES- 6 and PHQ-4 (Table 2) compared to those who did not engage in that type of information seeking. There was also a dose-response relation between number of types of information seeking 5+ times a day and meeting the elevated threshold on the IES-6 and PHQ-4, with individuals seeking information via multiple sources showing more than twice the odds of distress.

Information seeking was less strongly and consistently related to meeting the risk threshold on the UCLA-3. Checking news, social media, and mass communications increased the odds of loneliness between 38 to 48% (Table 2) for women doing so 5+ times a day compared to women who engaged in information seeking less than once a day. Discussing with others was only associated with increased loneliness when done 2-4x per day.

### Worries and mental health

**Fig 3:**
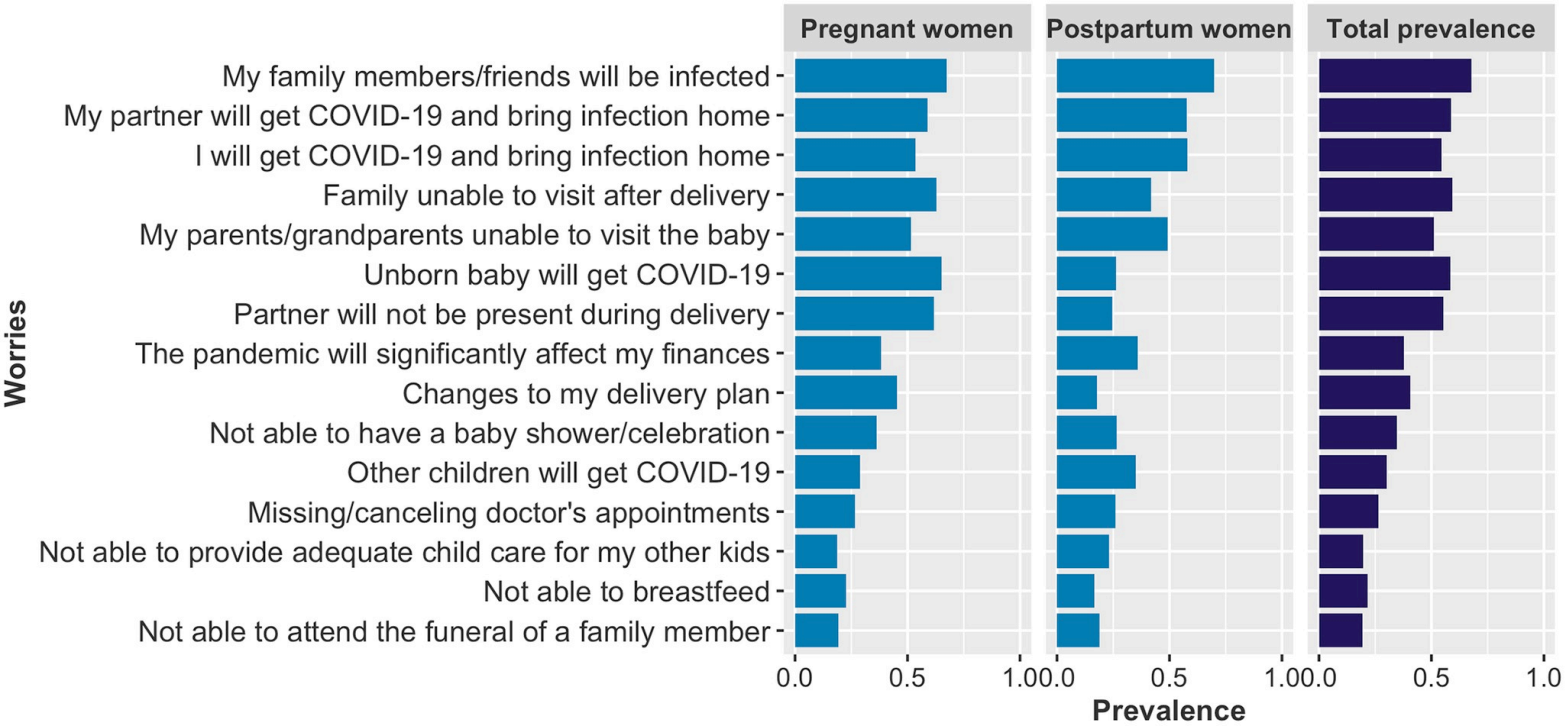
Prevalence of COVID-19 related worries for pregnant and postpartum women.

The prevalence of reported COVID-19 related worries is presented in Fig 3 and S1B Table. A high percentage of respondents (86%) reported being somewhat or very worried about COVID-19, with many of the most commonly reported worries related to pregnancy and delivery including: family being unable to visit after delivery (59%), the baby contracting COVID-19 (59%), lack of a support person during delivery (55%), and COVID-19 causing changes to the delivery plan (41%). Being very worried about COVID-19, compared to not having any worries, was strongly and significantly associated with meeting the cut-off both on the IES-6 (OR = 4.75, 95% CI: 3.34, 6.87) and the PHQ-4 (OR = 1.51, 95% CI: 1.09, 2.13) but was not associated with the UCLA-3 (OR = 1.25, 95% CI: 0.90, 1.72), in fully adjusted models.

**Fig 4:**
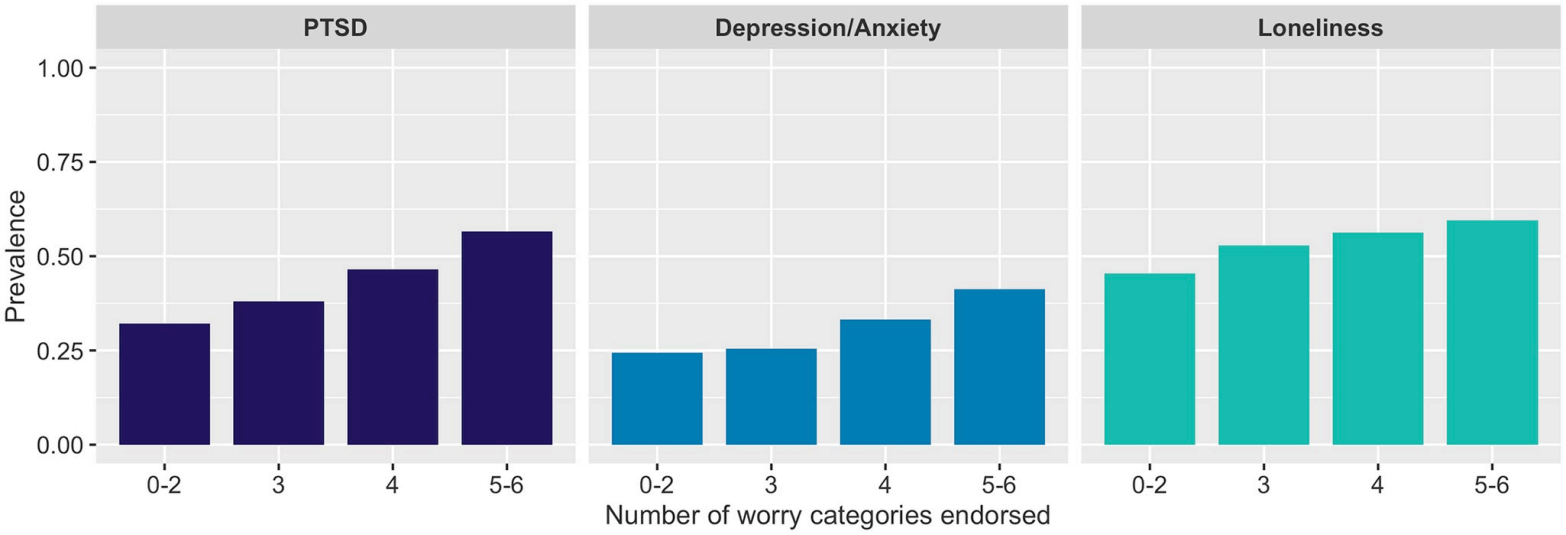
Prevalence of elevated symptoms of PTSD, depression/anxiety, and loneliness by the number of COVID-19-related worry categories endorsed by pregnant and postpartum women.

Using domains obtained from the factor analysis of worries (S2 Table), Table 3 presents the association between mental health outcomes and the six COVID-19 worry categories. Child-related worries and medical care worries were each associated with significantly higher odds of exceeding the threshold on the IES-6, PHQ4 and UCLA-3. Social worries were only associated with higher odds of loneliness. Economic worries were associated with increased odds of meeting the threshold for IES-6 and PHQ4; infection and delivery worries were only significantly associated with increased odds of meeting the threshold on the IES-6. The prevalence of elevated PTSD symptoms in relation to COVID-19, depression/anxiety, and loneliness consistently increased for every additional worry category endorsed (Fig 4).

**Table 3:** Results of multivariable logistic regression models for elevated symptoms of PTSD, depression/anxiety, and loneliness in relation to the six COVID-19 worry factors^a^.

	N (% of total pop.)	PTSD (IES-6)	Depression/Anxiety (PHQ4)	Loneliness (UCLA-3)
*Worry factors*		*OR (95% CI)*	*OR (95% CI)*	*OR (95% CI)*
Child	3132 (45.4%)	1.52 (1.37–1.69)*	1.54 (1.38–1.73)*	1.25 (1.12–1.38)*
Social	5043 (73.2%)	1.03 (0.91–1.16)	1.03 (0.91–1.18)	1.24 (1.10–1.39)*
Infection	5705 (82.8%)	1.48 (1.29–1.71)*	1.04 (0.89–1.21)	0.96 (0.84–1.10)
Delivery	5324 (77.2%)	1.40 (1.21–1.61)*	1.02 (0.88–1.19)	1.17 (1.02–1.34)
Economic	2612 (37.9%)	1.26 (1.13–1.39)*	1.36 (1.22–1.52)*	1.10 (0.99–1.21)
Medical care	1820 (26.4%)	1.43 (1.28–1.60)*	1.31 (1.17–1.48)*	1.26 (1.12–1.41)*

*p<0.001.

^a^ All models were adjusted for age, education, race/ethnicity, medical coverage status, survey region, marital status, weeks pregnant/postpartum.

Abbreviations: OR, odds ratios; pop., population.

### Behaviors and mental health

**Fig 5:**
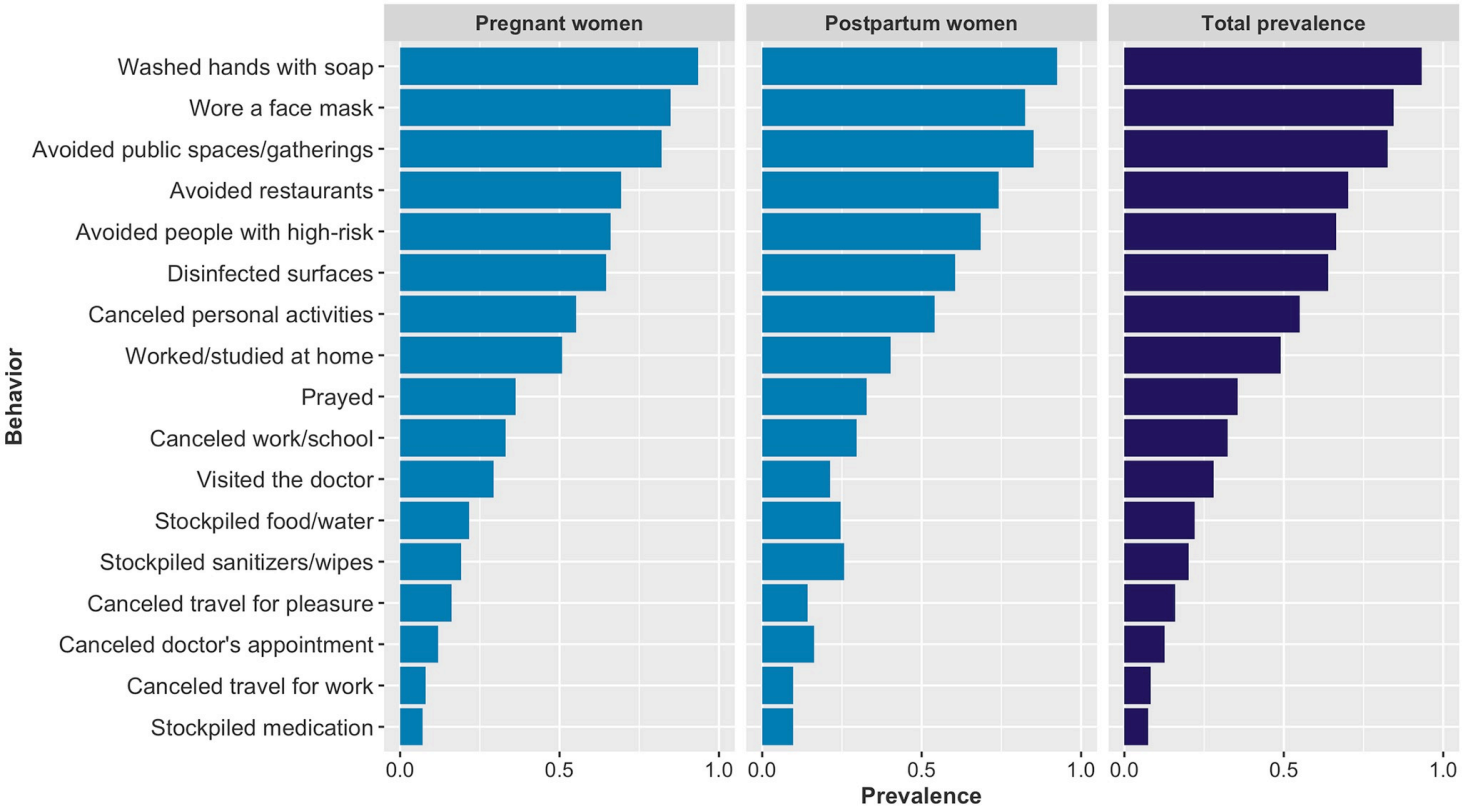
Prevalence of COVID-19 prevention related behaviors for pregnant and postpartum women.

The prevalence of reported engagement in COVID-19 prevention related behaviors is presented in Fig 5 and further details are provided in S1C Table. The majority of women engaged in COVID-19 prevention behaviors recommended by public health experts: 93% washed/sanitized hands several times per day, 85% wore a face mask, 83% avoided crowds, 70% avoided eating in restaurants, 67% avoided contact with high-risk people, 64% disinfected surfaces, and 55% canceled or postponed activities. This pattern of engagement was consistent across pregnancy stage. Results of logistic regression models for IES-6, PHQ-4 and UCLA-3 in relation to types of COVID-19 prevention behaviors are presented in Table 4.

**Table 4:** Results of logistic regression models for elevated symptoms of PTSD, depression/anxiety, and loneliness in relation to the six COVID-19 behavior groups^a^.

	N (% of total pop.)	PTSD (IES-6)	Depression/Anxiety (PHQ4)	Loneliness (UCLA-3)
*Behaviors*		*OR (95% CI)*	*OR (95% CI)*	*OR (95% CI)*
Distancing	6508 (94.4%)	1.5 (1.19, 1.89)*	1.22 (0.96, 1.56)	1.25 (1.01, 1.55)
Travel	1254 (18.2%)	1.03 (0.91, 1.18)	1.04 (0.90, 1.19)	0.99 (0.87, 1.13)
Hygiene	6687 (97.0%)	1.41 (1.04, 1.93)	0.77 (0.57, 1.04)	0.75 (0.56, 1.01)
Stockpiling	2297 (33.3%)	1.28 (1.14, 1.42)*	1.12 (0.99, 1.25)	1.03 (0.92, 1.15)
Medical care	2531 (36.7%)	1.25 (1.12, 1.38)*	1.19 (1.06, 1.33)	1.23 (1.10, 1.36)*
Prayer	2455 (35.6%)	1.31 (1.17, 1.46)*	1.06 (0.94, 1.19)	1.08 (0.97, 1.21)

*p<0.001.

^a^ All models were adjusted for age, education, race/ethnicity, medical coverage status, survey region, marital status, weeks pregnant/postpartum.

Abbreviations: OR, odds ratios; pop., population.

Hygiene and travel behaviors were not related to elevated IES-6, PHQ-4, or UCLA-3. However, distancing and stockpiling behaviors as well as canceling doctor’s appointments and prayer were associated with increased odds of meeting the threshold on the IES-6. None of the COVID-19 related prevention behaviors were associated with increased odds of meeting the PHQ-4 threshold. Only canceling doctor’s appointments was associated with increased loneliness.

## Discussion

In this large-scale anonymous, online, international cross-sectional survey of pregnant and postpartum women from 64 countries during the COVID-19 pandemic, the prevalence of elevated mental health symptoms was high, with 43%, 31%, and 54% of women exceeding elevated thresholds for PTSD symptoms in relation to COVID-19, depression/anxiety, and loneliness, respectively. Excessive pandemic-related information seeking was the strongest correlate of adverse mental health outcomes. Across any modality or type of media, information seeking five or more times per day, was associated with a more than two-fold increased risk of a positive screen for elevated posttraumatic stress and depression/anxiety symptoms. Moreover, there was no evidence that discussion about COVID-19, whether with another person or in social media, reduced loneliness. These findings are consistent with prior studies, including during the pandemic, that suggest that excessive information seeking might serve to exacerbate negative emotionality and eventually contribute to mental health problems [37–40]. However, it is also possible that excessive pandemic-related information-seeking may be a manifestation of mental health problems rather than a cause of them. For instance, women who are anxious may surveil media sources for ongoing risks [41] and/ or to regain a sense of control [42].

The vast majority of women in our study reported feeling somewhat to very worried about COVID-19, with large proportions reporting pregnancy or birth specific worries such as lack of social support around delivery. Child-related worries (inadequate childcare, other children will get COVID-19) and worries about missing doctor appointments were independently associated with increased odds of elevated symptoms in all outcomes. Most women reported following public health science supported COVID-19 hygiene behaviors (e.g., face mask wearing, washing hands). Such behaviors were not associated with mental health symptoms or loneliness.

Prevalence rates of psychiatric distress in our study exceed non-pandemic perinatal studies of prenatal and postpartum depression and anxiety [18, 19] and most general population estimates during pandemics [43]. Findings are especially notable as a very small proportion of women report COVID-19 infection and most women (> 75%) also reported that they were not exposed to anyone with a known infection. Recent commentaries highlight that women may be especially vulnerable to the impact of the pandemic’s effects and public health measures taken to reduce its effects, e.g., may be more likely to lose employment due to childcare demands given school closures [44]. These considerations may be particularly salient for pregnant and postpartum women, for whom pandemic associated worries and the physical distancing required for infection control may exacerbate mental health problems and feelings of loneliness. Given the potential implications for maternal and child health, acknowledging the pandemic’s unique impact on pregnant and postpartum women is central to tailoring and scaling public and mental health intervention efforts to address their particular needs.

Our findings on information seeking are consistent with others that have found high levels of pandemic-related information-seeking, whether social media or traditional news sources, is associated with increased prevalence of anxiety and depression [24, 25]. They are also consistent with recommendations from international policy relevant organizations (e.g., WHO) for healthy information seeking to mitigate the mental health impact of the pandemic [45, 46]. For example, recommendations include limiting the number of times one seeks the latest news updates and reducing total time spent on news that feels upsetting. Our results extend these findings to perinatal women and expand the modalities examined. Strikingly, we found that even discussing COVID-19 *with another person* five or more times a day was associated with a two-fold increase in posttraumatic stress and depression/anxiety. Discussing COVID-19, whether with another person or in mass communications (e.g., Facebook, Twitter) was not associated with reduced loneliness. In fact, there was evidence of higher loneliness in persons who engaged in such discussions frequently, suggesting that interpersonal engagement with others, while generally beneficial for mental health, may not be protective if strongly focused on the pandemic itself.

Degree of worry about COVID-19 was associated with elevated symptoms of posttraumatic stress and depression/anxiety but not loneliness. With regard to specific worries, child-related worries and missing medical appointments were consistently associated elevations in posttraumatic stress, depression/anxiety and loneliness. At the time of this study, we identified only one other study of pregnant or postpartum women that reported on specific worries [47]. Our findings, based on a data-driven approach to identify worry domains, were consistent with those reported by Corbett et al., who found that a majority of women had worries about the health of their relatives, children, and the unborn baby. Additionally, our results show that substantial proportions of women also have worries related to their delivery plan, with specific concerns about not being allowed a support person during delivery. Providing women with up-to-date, accurate information on how to prevent infection of themselves and their children, helping women understand the steps healthcare systems are taking to prevent infections of patients, especially newborns, and providing consistent prenatal and postpartum care may help support pregnant women’s mental health during this time.

With respect to behaviors to safeguard against the pandemic, most women reported engaging in prevention efforts recommended by public health experts i.e., hand hygiene, wearing a face mask, avoiding crowds and public places such as restaurants, avoiding contact with high-risk individuals, and disinfecting surfaces. Most notably, hygiene-promoting behaviors were not associated with elevated mental disorder symptoms or loneliness. While hygiene promotion behaviors are central to containing the viral spread and mitigating risk, engaging in such behaviors (or not) maybe unrelated to mental health. This has important implications for clinical research and intervention efforts as it suggests that different interventions may be needed to address the infectious disease aspects of the pandemic versus the mental health and emotional well-being aspects. Associations between other pandemic-related prevention behaviors and mental health and loneliness were inconsistent.

Our study findings should be interpreted in light of several limitations. The cross-sectional nature of the study prevents any causal attributions between the factors we examined. For instance, distancing behaviors and postponing medical care to prevent the contraction of COVID-19 (but not changes to travel plans and hygiene behaviors) were associated with risk for elevated symptoms of posttraumatic stress. Women who experience elevated posttraumatic stress symptoms may also be more likely to isolate themselves; social avoidance is part of the syndrome. Social distancing efforts may also perpetuate or exacerbate posttraumatic stress [48].

Additionally, our study findings are based on a convenience sample and thus not-representative of any country or region. For instance, relative to population-based studies of perinatal and postpartum women our sample included women who were more educated and older. Women in our study may also have been more active on social media platforms, which was our primary modality of recruitment. Our study also did not include women under the age of 18. Accordingly, our study likely also does not represent a comprehensive range of concerns and mental health needs of pregnant or postpartum women, especially those who may be disproportionately affected by the pandemic such as those with limited internet access or pregnant teens. Additionally, our study used U.S. centric race/ ethnic classifications, which may not correspond to classifications in other regions or countries in the world limiting our ability to examine such classifications cross-nationally. Finally, our study presents findings from a cross-section of responses between the end of May and early June 2020. The COVID-19 pandemic is on-going with case rates changing over time, and a variable course in different regions and countries. Accordingly, the mental health impact may also have changed since the study was conducted, and more recent assessments may yield different estimates of mental health symptoms. However, given the large sample size, the availability of the survey in multiple languages, and the paucity of perinatal mental health data for pregnant and postpartum women during the COVID-19 pandemic, our study provides initial information for future research and intervention efforts.

## Conclusion

More pregnant and postpartum women are self-reporting elevated symptoms of depression, anxiety, and posttraumatic stress than most available general population estimates of psychiatric distress during the pandemic. Such high levels of distress may have potential implications for women, and fetal and child health and development. Thus, efforts to screen, monitor, and target a range of mental health symptoms, and other aspects of emotional well-being, such as feelings of loneliness and worries, and coping behaviors (e.g., appropriate information seeking) should be considered. Given the pandemic is ongoing and the likely bi-directional nature of mental health symptoms and excessive information seeking behaviors, both broader public education campaigns and those focused on pregnant and postpartum women should address both elements. Our data further suggest that a population mental health approach to pregnant and postpartum women’s mental health needs to extend beyond medical and mental health needs and also address the unique challenges posed by the pandemic with respect to specific concerns related to child well-being and access to medical care. Thus, public health and medical system interventions need to consider the broad range of pandemic-related stressors as prevention of viral exposure itself, while critical, may not mitigate the pandemic’s mental health impact.

## Supporting information

S1 Fig A. Informed consent.
(PDF)

S2 Fig A. Tetrachoric correlation matrix of the COVID-19 worries questionnaire. B. Scree plot and parallel analysis of the COVID-19 worries questionnaire.
(PDF)

S1 Table A. Results of logistic regression models for elevated symptoms of PTSD, depression/anxiety, and loneliness in relation to socio-demographic characteristics and COVID-19 exposure. B. Prevalence of specific worries for the overall sample and by pregnancy stage. C. Prevalence of COVID-19 prevention behaviors for the overall sample and by pregnancy stage.
(PDF)

S2 Table A. Factor loadingsa based on a tetrachoric factor analysis with oblimin rotation of the COVID-19 worries questionnaire.
(PDF)

S1 Text Pregistry survey. (PDF)
