# Supplementary material for: A cross-national study of factors associated with women’s perinatal mental health and wellbeing during the COVID-19 pandemic
Source: PLoS One. 2021 Apr 21;16(4):e0249780. doi: 10.1371/journal.pone.0249780 (PMC8059819; doi:10.1371/journal.pone.0249780)
Supplement: S1 Fig — A. Informed consent. (PDF) [file pone.0249780.s001.pdf]

S1 Fig

S1 Fig A. Informed Consent.

Permission to Take Part in a Human Research Study

You are being asked to take part in a research study.

This research is being conducted to learn about the responses of pregnant and recently pregnant women to COVID-19. Specifically, we are interested in learning about the wellbeing and mental health of women during the COVID-19 pandemic.

You are being asked to participate in this research because you are 18 years of age or older, self-identify as pregnant or recently pregnant (within the last 6 months), are fluent in the English language, and have access to a computer, tablet, or smartphone with an Internet connection.

If you take part in this study, you will be asked to complete a brief (20-minute) online survey. Specifically, you will be asked a number of questions about your experience with COVID-19 and your feelings about it. This study is anonymous, and information about your identity will not be collected.

The possible risks of participating in this study include coming across questions or answer choices that you find unpleasant or upsetting. For instance, a few of the questions may cause you to think about COVID-19 concerns or negative emotional states.

We cannot promise any benefits to you or others from your taking part in this research. However, possible benefits include importance of knowledge to be gained for other pregnant women like you.

You can decline to participate in any part of this study for any reason and can end your participation at any time.

If you have any questions about this study, you can contact Karestan Koenen, PhD and Sonia Hernandez-Diaz, MD, MPH, DrPh, Department of Epidemiology, Harvard T. H. Chan School of Public Health and Diego Wyszynski, MD, MHS, PhD, Pregistry, at IRCEP@pregistry.com.

Thank you again for your time and participation.

☐ By clicking here I agree to the above Permission to Take Part in a Human Research Study
