# Supplementary material for: A cross-national study of factors associated with women’s perinatal mental health and wellbeing during the COVID-19 pandemic
Source: PLoS One. 2021 Apr 21;16(4):e0249780. doi: 10.1371/journal.pone.0249780 (PMC8059819; doi:10.1371/journal.pone.0249780)

S2 Fig A. Tetrachoric correlation matrix of the COVID-19 worries questionnaire.

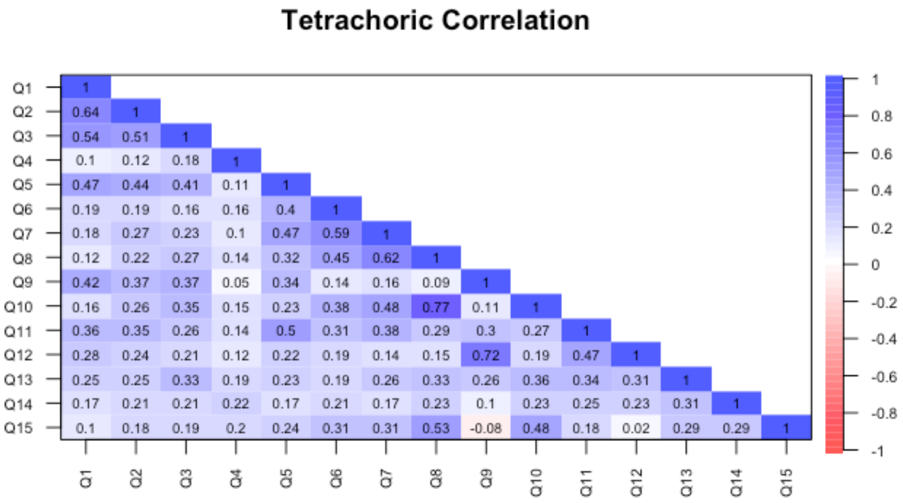

Q1 – participant will bring infection home; Q2 – partner will bring infection home; Q3 – family/friends will be infected with COVID-19; Q4 – COVID-19 will significantly affect economic situation/finances; Q5 – unborn baby will get COVID-19; Q6 – COVID-19 will cause changes to delivery plan; Q7 – partner will not be present during delivery because of COVID-19; Q8 – family unable to visit; Q9 – other children will get COVID-19; Q10 – parents/grandparents unable to visit; Q11 – not able to breastfeed because of COVID-19; Q12 – not able to provide adequate childcare for other kids; Q13 – not able to attend the funeral of a family member; Q14 – missing doctor appointments; Q15 – not able to have a baby shower/other baby celebration with family and friends

S2 Fig B. Scree plot and parallel analysis of the COVID-19 worries questionnaire.

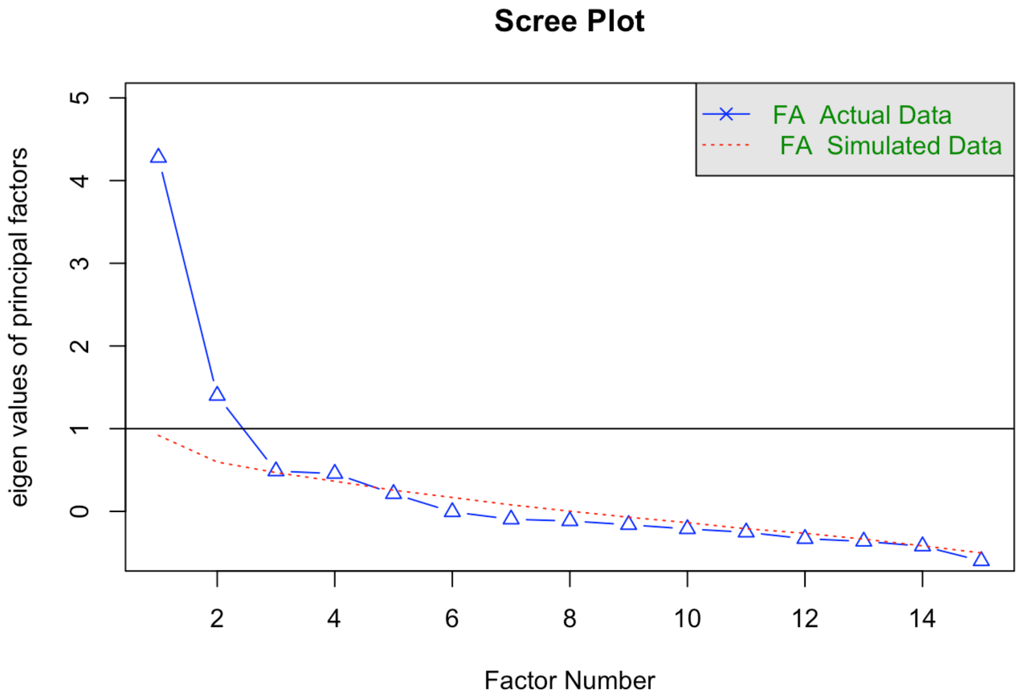

Supplement: S2 Fig — A. Tetrachoric correlation matrix of the COVID-19 worries questionnaire. B. Scree plot and parallel analysis of the COVID-19 worries questionnaire. (PDF) [file pone.0249780.s002.pdf]
