# Supplementary material for: A cross-national study of factors associated with women’s perinatal mental health and wellbeing during the COVID-19 pandemic
Source: PLoS One. 2021 Apr 21;16(4):e0249780. doi: 10.1371/journal.pone.0249780 (PMC8059819; doi:10.1371/journal.pone.0249780)
Supplement: S1 Table — A. Results of logistic regression models for elevated symptoms of PTSD, depression/anxiety, and loneliness in relation to socio-demographic characteristics and COVID-19 exposure. B. Prevalence of specific worries for the overall sample and by pregnancy stage. C. Prevalence of COVID-19 prevention behaviors for the overall sample and by pregnancy stage. (PDF) [file pone.0249780.s003.pdf]

S1 Table A. Results of logistic regression models for elevated symptoms of PTSD, depression/anxiety, and loneliness in relation to socio-demographic characteristics and COVID-19 exposure.

	**PTSD (IES-6)**		**Depression/Anxiety (PHQ4)**		**Loneliness (UCLA-3)**	
	OR (95% CI)	*P value*	*OR (95% CI)*	*P value*	*OR (95% CI)*	*P value*
(Intercept)	0.72 (0.47 – 1.09)	0.124	1.43 (0.91 – 2.23)	0.121	1.64 (1.09 – 2.49)	0.019
Age in years	0.98 (0.97 – 0.99)	<0.001	0.96 (0.95 – 0.97)	<0.001	0.98 (0.97 – 0.99)	0.001
Education						
High school graduate or less	*Reference*		*Reference*		*Reference*	
Some college	1.10 (0.91 – 1.33)	0.307	0.97 (0.80 – 1.18)	0.779	0.92 (0.76 – 1.11)	0.386
College graduate	1.19 (1.02 – 1.40)	0.032	0.87 (0.73 – 1.02)	0.091	1.00 (0.85 – 1.17)	0.992
Graduate school or more	1.15 (0.97 – 1.35)	0.101	0.80 (0.68 – 0.95)	0.012	0.91 (0.78 – 1.07)	0.272
Race/ethnicity						
White	*Reference*		*Reference*		*Reference*	
Latin/Hispanic	1.09 (0.90 – 1.32)	0.363	0.82 (0.67 – 1.00)	0.047	0.74 (0.61 – 0.89)	0.002
Asian	1.74 (1.36 – 2.24)	<0.001	1.27 (0.98 – 1.67)	0.076	0.84 (0.66 – 1.06)	0.144
Black	1.34 (1.07 – 1.68)	0.012	1.03 (0.81 – 1.31)	0.798	0.79 (0.63 – 0.99)	0.040
South Asian	1.72 (1.14 – 2.57)	0.009	0.90 (0.56 – 1.41)	0.649	1.02 (0.69 – 1.53)	0.917
Middle Eastern	1.14 (0.68 – 1.87)	0.620	1.00 (0.58 – 1.69)	0.990	0.45 (0.27 – 0.73)	0.002
Native/Indigenous	1.53 (0.67 – 3.50)	0.312	0.97 (0.40 – 2.25)	0.947	0.35 (0.14 – 0.81)	0.017
More than 1	1.04 (0.80 – 1.33)	0.783	0.94 (0.71 – 1.22)	0.629	0.78 (0.61 – 1.00)	0.054
Other	1.00 (0.74 – 1.35)	0.991	0.78 (0.57 – 1.08)	0.138	0.67 (0.50 – 0.90)	0.008
Missing indicator	1.78 (1.07 – 3.00)	0.027	0.97 (0.56 – 1.65)	0.918	1.16 (0.70 – 1.96)	0.560
Medical coverage status						
No	*Reference*		*Reference*		*Reference*	
Yes	0.92 (0.82 – 1.03)	0.149	0.70 (0.63 – 0.79)	<0.001	0.94 (0.84 – 1.05)	0.258
Region						

Asia & Pacific	*Reference*		*Reference*		*Reference*	
Africa	1.91 (1.43 – 2.56)	<0.001	1.54 (1.13 – 2.10)	0.006	1.36 (1.03 – 1.81)	0.031
Europe	1.52 (1.18 – 1.95)	0.001	0.92 (0.70 – 1.21)	0.566	1.32 (1.04 – 1.68)	0.023
Middle East	3.65 (2.20 – 6.15)	<0.001	2.15 (1.29 – 3.59)	0.003	1.45 (0.89 – 2.38)	0.139
North America	1.60 (1.25 – 2.07)	<0.001	1.31 (1.00 – 1.72)	0.055	1.66 (1.31 – 2.12)	<0.001
South/Latin America	1.45 (1.10 – 1.90)	0.008	1.81 (1.36 – 2.42)	<0.001	1.12 (0.86 – 1.45)	0.413
Marital status						
Married	*Reference*		*Reference*		*Reference*	
Living with partner	0.93 (0.83 – 1.05)	0.226	1.04 (0.91 – 1.18)	0.575	1.01 (0.90 – 1.14)	0.839
Other	1.16 (0.97 – 1.40)	0.109	1.59 (1.31 – 1.91)	<0.001	1.80 (1.49 – 2.18)	<0.001
Weeks pregnant/postpartum						
0 to <13 weeks	*Reference*		*Reference*		*Reference*	
13 to <28 weeks	1.16 (1.01 – 1.33)	0.041	0.93 (0.80 – 1.08)	0.348	1.12 (0.98 – 1.29)	0.106
28+ weeks	1.10 (0.95 – 1.27)	0.215	0.97 (0.83 – 1.13)	0.669	1.21 (1.04 – 1.40)	0.011
Postpartum	1.27 (1.08 – 1.50)	0.004	1.06 (0.89 – 1.26)	0.539	1.28 (1.09 – 1.51)	0.003
Tested for COVID-19						
No, I have not been tested	*Reference*		*Reference*		*Reference*	
Negative, I did not have the virus	0.78 (0.65 – 0.93)	0.005	1.02 (0.85 – 1.23)	0.814	0.81 (0.68 – 0.96)	0.014
Positive, I had the virus	0.72 (0.40 – 1.27)	0.259	1.39 (0.77 – 2.50)	0.265	0.92 (0.52 – 1.64)	0.776
Yes, but I do not know the results yet or the result was inconclusive	1.33 (0.84 – 2.13)	0.221	0.98 (0.59 – 1.59)	0.934	0.70 (0.44 – 1.10)	0.123
In contact with someone who has/had COVID-19						
No	*Reference*		*Reference*		*Reference*	
Maybe	1.41 (1.23 – 1.62)	<0.001	1.44 (1.25 – 1.67)	<0.001	1.33 (1.16 – 1.53)	<0.001
Yes	1.20 (0.99 – 1.46)	0.063	1.46 (1.18 – 1.79)	<0.001	1.09 (0.90 – 1.33)	0.372
Diagnosed with COVID-19						
No	*Reference*		*Reference*		*Reference*	

Yes, and I still have it	1.36 (0.64 – 2.90)	0.415	1.84 (0.85 – 4.01)	0.120	1.45 (0.69 – 3.15)	0.333
Yes, but I recovered	0.88 (0.55 – 1.41)	0.605	0.98 (0.59 – 1.60)	0.938	0.87 (0.55 – 1.40)	0.566

S1 Table B. Prevalence of specific worries for the overall sample and by pregnancy stage.

Worries	Pregnant	Postpartum	Overall
That my family members/friends will be infected with COVID-19	67.4%	69.8%	67.8%
That my family will not be able to visit me and the baby after delivery because of measures to prevent COVID-19 spread	62.8%	41.7%	59.2%
That my partner will get COVID-19 and bring the infection home	59.0%	57.6%	58.8%
That my unborn baby will get COVID-19	65.2%	26.3%	58.5%
That my partner/support person will not be able to be with me during delivery because of COVID-19	61.8%	24.5%	55.4%
That I will get COVID-19 and bring the infection home	53.6%	58.0%	54.4%
That my parents/grandparents will not be able to visit the baby because of measures to stop COVID-19	51.4%	49.2%	51.0%
That COVID-19 will mean changes to my delivery plan	45.4%	17.9%	40.6%
That the COVID-19 pandemic will significantly affect my economic situation/finances (for example, lose my job)	38.3%	36.0%	37.9%
That I will not be able to have a baby shower or other baby celebration with family or friends	36.2%	26.6%	34.5%
That my other children will get COVID-19	29.0%	35.0%	30.0%
Missing/canceling doctor's appointments	26.5%	25.9%	26.4%
That I will not be able to breastfeed because of COVID-19	22.7%	16.7%	21.7%
That I will not be able to provide adequate childcare for my other kids	18.8%	23.1%	19.6%
That I will not be able to attend the funeral of a family member	19.3%	18.9%	19.2%

S1 Table C. Prevalence of COVID-19 prevention behaviors for the overall sample and by pregnancy stage.

COVID-19 Behavior	Pregnant	Postpartum	Total
Washed your hands with soap or used hand sanitizer several times per day	93.4%	92.5%	93.3%
Wore a face mask	84.9%	82.5%	84.5%
Avoided public spaces, gatherings, or crowds	82.1%	85.2%	82.6%
Avoided eating at restaurants	69.3%	74.2%	70.1%
Avoided contact with people who could be high-risk	66.1%	68.4%	66.5%
Disinfected surfaces around you	64.6%	60.6%	63.9%
Canceled or postponed personal or social activities	55.2%	54.1%	55.0%
Worked or studied at home	50.8%	40.3%	49.0%
Prayed	36.2%	32.7%	35.6%
Canceled or postponed work or school activities	33.1%	29.6%	32.5%
Visited a doctor	29.3%	21.3%	27.9%
Stockpiled food or water	21.6%	24.5%	22.1%
Stockpiled hand sanitizer or disinfectant wipes	19.1%	25.7%	20.2%
Canceled or postponed air travel for pleasure	16.2%	14.2%	15.9%
Canceled a doctor's appointment	11.9%	16.2%	12.7%
Canceled or postponed air travel for work	8.0%	9.6%	8.3%
Stockpiled medication	7.1%	9.6%	7.5%
