# Supplementary material for: A cross-national study of factors associated with women’s perinatal mental health and wellbeing during the COVID-19 pandemic
Source: PLoS One. 2021 Apr 21;16(4):e0249780. doi: 10.1371/journal.pone.0249780 (PMC8059819; doi:10.1371/journal.pone.0249780)
Supplement: S2 Table — A. Factor loadingsa based on a tetrachoric factor analysis with oblimin rotation of the COVID-19 worries questionnaire. (PDF) [file pone.0249780.s004.pdf]

S2 Table. A. Factor loadings[a] based on a tetrachoric factor analysis with oblimin rotation of the COVID-19 worries questionnaire.

Worried that	Factor 1: Social	Factor 2: Infection	Factor 3: Child	Factor 4: Delivery
Parents/grandparents unable to visit	0.88			
Family unable to visit	0.78			
Not able to have a baby shower/other baby celebration with family and friends	0.53			
Not able to attend the funeral of a family member	0.33			
Missing doctor appointments				
COVID-19 will significantly affect economic situation/finances				
Participant brings infection home		0.80		
Partner will get COVID-19 and bring the infection home		0.71		
Family/friends will be infected with COVID-19		0.68		
Not able to provide adequate childcare for other kids			1.02	
Other children will get COVID-19			0.66	
Partner will not be present during delivery because of COVID-19				0.72
COVID-19 will cause changes to delivery plan				0.62
Unborn baby will get COVID-19		0.41		0.56
Not able to breastfeed because of COVID-19			0.35	0.37

[a]All loadings >.30 appear in the table
