# Supplementary material for: A cross-national study of factors associated with women’s perinatal mental health and wellbeing during the COVID-19 pandemic
Source: PLoS One. 2021 Apr 21;16(4):e0249780. doi: 10.1371/journal.pone.0249780 (PMC8059819; doi:10.1371/journal.pone.0249780)
Supplement: S1 Text — (PDF) [file pone.0249780.s005.pdf]

S1 Text. Pregistry survey

English Pregistry survey

In which country do you live?
***list of countries
In which state/province do you live?

In which city do you live?

Are you pregnant?
 Yes
 No

How many weeks pregnant are you?
***list 5 to 43

How old are you?
***list 18 to 50

Which race(s) do you most identify with? (check all that apply)
 White/Caucasian
 Latin/Hispanic
 Asian
 South Asian
 Black
 Middle Eastern
 Native Hawaiian or Other Pacific Islander
 American Indian or Alaska Native
 Other/Multirace

What is your marital status?
 Single

Married
Living with partner
Separated
Divorced
Widowed

How many people live in your household (including yourself)?

1
2
3
4
5
6
7
8
9
10
More than 10

What is the highest level of education you attained?

Never attended school
Elementary school
Some high school
High school graduate or general equivalency diploma (GED)
Some college/university
College diploma or university degree
Masters degree
Professional degree
Doctoral degree

Please indicate if during the COVID-19 pandemic you:

Were a healthcare worker in a hospital or clinic
Worked in a nursing home

Were an essential/key worker (as defined by the government)
None of these
Don’t know

Do you have medical insurance through your government, your employer, or a family member?
Yes
No

Have you been tested for SARS-CoV-2 (the virus that causes COVID-19)? If so, what was the result?
Positive, I had the virus
Negative, I did not have the virus
Yes, but I do not know the result yet or the result was inconclusive
No, I have not been tested

Has a health care professional (e.g., doctor, nurse) diagnosed you as having COVID-19 based on your symptoms?
No
Yes, and I still have it
Yes, but I recovered

Have you been in contact with an individual who has or had COVID-19?
Yes
No
Maybe

Which of the following have you done in the last 7 days to keep yourself and others safe from COVID-19? (Select all that apply)
Wore a face mask
Washed your hands with soap or used hand sanitizer several times per day
Disinfected surfaces around you
Stockpiled hand sanitizer or disinfectant wipes
Stockpiled food or water
Canceled or postponed air travel for work
Canceled or postponed air travel for pleasure
Canceled or postponed work or school activities

Canceled or postponed personal or social activities
Avoided contact with people who could be high-risk
Avoided public spaces, gatherings, or crowds
Avoided eating at restaurants
Worked or studied at home
Visited a doctor
Canceled a doctor's appointment
Stockpiled medication
Prayed

How often do you check the news about COVID-19?
Never
<1 x/day
1 x/day
2-4 x/day
5-8 x/day
9-16 x/day
>16 x/day

How often do you check social media about COVID-19? (e.g. WhatsApp, Facebook)
Never
<1 x/day
1 x/day
2-4 x/day
5-8 x/day
9-16 x/day
>16 x/day

How often do you discuss COVID-19 in mass communications? (e.g. WhatsApp group, Twitter)
Never
<1 x/day
1 x/day
2-4 x/day

5-8 x/day
9-16 x/day
>16 x/day

How often do you discuss COVID-19 with another person?
Never
<1 x/day
1 x/day
2-4 x/day
5-8 x/day
9-16 x/day
>16 x/day

How worried are you about COVID-19?
Very worried
Somewhat worried
Not very worried
Not worried at all

What about COVID-19 makes you most worried? Select all that apply:
That I will get COVID-19 and bring the infection home
That my partner will get COVID-19 and bring the infection home
That my family members/friends will be infected with COVID-19
That the COVID-19 pandemic will significantly affect my economic situation/finances (for example, lose my job)
That my unborn baby will get COVID-19
That COVID-19 will mean changes to my delivery plan
That my partner/support person will not be able to be with me during delivery because of COVID-19
That my family will not be able to visit me and the baby after delivery because of measures to prevent COVID-19 spread
That my other children will get COVID-19
That my parents/grandparents will not be able to visit the baby because of measures to stop COVID-19
That I will not be able to breastfeed because of COVID-19
That I will not be able to provide adequate child care for my other kids
That I will not be able to attend the funeral of a family member

Missing/canceling doctor's appointments
That I will not be able to have a baby shower or other baby celebration with family or friends
Other

To what extent has COVID-19 negatively impacted the following areas of your life?
Sleep
Not at all
A little bit
Moderately
A lot
Diet
Not at all
A little bit
Moderately
A lot
Fitness
Not at all
A little bit
Moderately
A lot
Work
Not at all
A little bit
Moderately
A lot
Finances
Not at all
A little bit
Moderately
A lot
Family
Not at all
A little bit

Moderately
A lot
Relationships
Not at all
A little bit
Moderately
A lot
Religious observance
Not at all
A little bit
Moderately
A lot
Character (e.g., patience, trust)
Not at all
A little bit
Moderately
A lot

Since the COVID-19 pandemic started, how often have any of the following things happened to you?
You were treated with less courtesy than other people are
Almost every day
At least once a week
A few times a month
Never
You received poorer service than other people at restaurants or stores
Almost every day
At least once a week
A few times a month
Never
People acted as if they are afraid of you
Almost every day
At least once a week
A few times a month

Never

You were called names or insulted

Almost every day

At least once a week

A few times a month

Never

You were threatened or harassed

Almost every day

At least once a week

A few times a month

Never

You were physically assaulted

Almost every day

At least once a week

A few times a month

Never

What do you think is the main reason for these experiences? (Choose all that apply)

Your ancestry or national origins

Your gender

Your race or ethnicity

Your age

Your religion

Your height

Your weight

Your pregnancy

Your sexual orientation

Your education or income level

Other

Please answer the following questions based on the last 2 weeks.

How often have you felt that you were unable to control the important things in your life?

Never

Almost Never
Sometimes
Fairly Often
Very Often
How often have you felt confident about your ability to handle your personal problems?
Never
Almost Never
Sometimes
Fairly Often
Very Often
How often have you felt that things were going your way?
Never
Almost Never
Sometimes
Fairly Often
Very Often
How often have you felt difficulties were piling up so high that you could not overcome them?
Never
Almost Never
Sometimes
Fairly Often
Very Often

How often have you been bothered by the following problems during the last 2 weeks?
Feeling nervous, anxious or on edge
Not at all
Several Days
More than half the days
Nearly every day
Not being able to stop or control worrying
Not at all
Several Days
More than half the days

Nearly every day
Feeling down, depressed or hopeless
Not at all
Several Days
More than half the days
Nearly every day
Little interest or pleasure in doing things
Not at all
Several Days
More than half the days
Nearly every day

Since the COVID-19 crisis began, how often do you eat...
Because you’re depressed or sad
Almost never or never
Rarely
Sometimes
Often
Almost always or always
As a way to help you cope
Almost never or never
Rarely
Sometimes
Often
Almost always or always
As a way to comfort yourself
Almost never or never
Rarely
Sometimes
Often
Almost always or always
Because you feel worthless or inadequate
Almost never or never

Rarely
Sometimes
Often
Almost always or always
As a way to avoid thinking about something unpleasant or to distract yourself
Almost never or never
Rarely
Sometimes
Often
Almost always or always

Indicate how much you agree with the following statements
I have so much in life to be thankful for
Strongly disagree
Disagree
Somewhat disagree
Neither agree nor disagree
Somewhat agree
Agree
Strongly agree
If I had to list everything that I felt grateful for, it would be a very long list
Strongly disagree
Disagree
Somewhat disagree
Neither agree nor disagree
Somewhat agree
Agree
Strongly agree
I am grateful to a wide variety of people
Strongly disagree
Disagree
Somewhat disagree
Neither agree nor disagree

Somewhat agree
Agree
Strongly agree

Please describe how the COVID-19 crisis has impacted you the most:

Since the COVID-19 crisis began, how supportive are the following people?
Husband or significant other
Not at all
A little bit
Moderately
Quite a bit
Extremely
N/A (there is no such person)
Parents or legal guardians
Not at all
A little bit
Moderately
Quite a bit
Extremely
N/A (there is no such person)
Children
Not at all
A little bit
Moderately
Quite a bit
Extremely
N/A (there is no such person)
Siblings
Not at all
A little bit
Moderately
Quite a bit

Extremely
N/A (there is no such person)
Friends
Not at all
A little bit
Moderately
Quite a bit
Extremely
N/A (there is no such person)
Co-workers
Not at all
A little bit
Moderately
Quite a bit
Extremely
N/A (there is no such person)

Since the COVID-19 crisis began, how stressed do you feel by the following people?
Husband or significant other
Not at all
A little bit
Moderately
Quite a bit
Extremely
N/A (there is no such person)
Parents or legal guardians
Not at all
A little bit
Moderately
Quite a bit
Extremely
N/A (there is no such person)
Children

Not at all
A little bit
Moderately
Quite a bit
Extremely
N/A (there is no such person)
Siblings
Not at all
A little bit
Moderately
Quite a bit
Extremely
N/A (there is no such person)
Friends
Not at all
A little bit
Moderately
Quite a bit
Extremely
N/A (there is no such person)
Co-workers
Not at all
A little bit
Moderately
Quite a bit
Extremely
N/A (there is no such person)

Since the COVID-19 crisis began…
How often do you feel that you lack companionship?
Hardly ever
Some of the time
Often

How often do you feel left out?
Hardly ever
Some of the time
Often
How often do you feel isolated from others?
Hardly ever
Some of the time
Often

Thinking about your experience IN THE PAST 7 DAYS, please indicate how strongly you agree or disagree with each of the following statements
I don’t feel I belong to anything I’d call a community
Strongly agree
Agree
Somewhat agree
Neither agree nor disagree
Somewhat disagree
Disagree
Strongly disagree
I have something valuable to give the world
Strongly agree
Agree
Somewhat agree
Neither agree nor disagree
Somewhat disagree
Disagree
Strongly disagree
I feel close to other people in my community
Strongly agree
Agree
Somewhat agree
Neither agree nor disagree
Somewhat disagree

Disagree
Strongly disagree
I cannot make sense of what's going on in the world
Strongly agree
Agree
Somewhat agree
Neither agree nor disagree
Somewhat disagree
Disagree
Strongly disagree
My community is a source of comfort
Strongly agree
Agree
Somewhat agree
Neither agree nor disagree
Somewhat disagree
Disagree
Strongly disagree
I believe that people are kind
Strongly agree
Agree
Somewhat agree
Neither agree nor disagree
Somewhat disagree
Disagree
Strongly disagree

Please answer the following questions based on the last 7 days
I feel watchful or on-guard
Not at all
A little bit
Moderately
Quite a bit

Extremely
Other things keep making me think about COVID-19
Not at all
A little bit
Moderately
Quite a bit
Extremely
I am aware that I have a lot of feelings about COVID-19, but I don’t deal with them
Not at all
A little bit
Moderately
Quite a bit
Extremely
I try not to think about COVID-19
Not at all
A little bit
Moderately
Quite a bit
Extremely
I have trouble concentrating
Not at all
A little bit
Moderately
Quite a bit
Extremely

Please provide any feedback about this survey:

How did you hear about us?
Google
Facebook ad
Pregistry Facebook page
Harvard website

LinkedIn
A post from a friend on social media
Word of mouth
Others

German Pregistry survey

In welchem Land leben Sie?
(***list of all countries)

In welchem Bundesstaat/welcher Provinz leben Sie?

In welcher Stadt leben Sie?

Bist du schwanger?
Ja
Nein

In der wievielten Schwangerschaftswoche (SSW) sind Sie?
(***list from 5 to 43)

Wie lange ist es her, dass du geboren hast?
(***list not transferred)

Wie alt sind Sie?
(***list from 18 to 50)

Mit welchen Gruppen identifizieren Sie sich am meisten? (Zutreffendes bitte ankreuzen)
Weiß/Kaukasier
Latina/ Hispanisch
Asiatisch
Süd-asiatisch
Schwarz
MITTLERER OSTEN
Einheimische Hawaiianer oder andere pazifische Insulaner
Indianer oder Alaska-Ureinwohner
Andere/Multirace

Wie ist Ihr Familienstand?
Single
Verheiratet
Mit Partner lebend
Getrennt
Geschieden
Verwitwet

Wie viele Menschen leben in Ihrem Haushalt (einschließlich Sie selbst)?
1
2
3
4
5
6
7
8
9
10
Mehr als 10

Was ist das höchste Bildungsniveau, das Sie erreicht haben?
Nie zur Schule gegangen
Grundschule
Eine High School
Abitur oder allgemeines Äquivalenzdiplom (GED)
Ein College / eine Universität
Hochschuldiplom oder Universitätsabschluss
Master-Studium
Berufsabschluss
Doktortitel

Bitte geben Sie an, ob Sie während der COVID-19-Pandemie:

als Angestellte im Gesundheitswesen in einem Krankenhaus oder einer Klinik arbeiteten
in einem Pflegeheim arbeiteten
ein wesentlicher/ systemrelevanter Arbeitnehmer waren
Keine von diesen
Weiß nicht

Haben Sie eine Krankenversicherung durch Ihre Regierung, Ihren Arbeitgeber oder ein Familienmitglied?
Ja
Nein

Wurden Sie auf SARS-CoV-2 (den Virus, der COVID-19 verursacht) getestet? Wenn ja, wie war das Ergebnis?
Positiv, ich hatte das Virus
Negativ, ich hatte das Virus nicht
Ja, aber ich kenne das Ergebnis noch nicht oder das Ergebnis war nicht schlüssig
Nein, ich wurde nicht getestet

Hat jemand vom Gesundheitspersonal (z. B. ein Arzt oder eine Krankenschwester) bei Ihnen nur aufgrund Ihrer Symptome COVID-19 diagnostiziert?
Nein
Ja, und ich habe es immer noch
Ja, aber ich habe mich erholt

Hatten Sie Kontakt zu einer Person, die COVID-19 hat oder hatte?
(***list not transferred)

Welche der folgenden Maßnahmen haben Sie in den letzten 7 Tagen ergriffen, um sich und andere vor COVID-19 zu schützen?
(Wählen Sie alle zutreffenden)
Sie haben einen Mundschutz getragen
Sie haben Ihre Hände mehrmals täglich mit Seife gewachsen oder ein Händedesinfektionsmittel verwendet
Sie haben die Sie umgebenden Oberflächen desinfiziert
Einen Vorrat an Händedesinfektionsmitteln oder Desinfektionstüchern angelegt
Einen Vorrat an Lebensmitteln oder Wasser angelegt
Arbeitsflüge abgesagt oder verschoben

Flugreisen zum Vergnügen abgesagt oder verschoben
Arbeits- oder Schulaktivitäten abgesagt oder verschoben
Persönliche oder soziale Aktivitäten angesagt oder verschoben
Den Kontakt mit Personen vermieden, die ein hohes Risiko darstellen könnten
Öffentliche Plätze, Versammlungen und Menschenmassen gemieden
Es vermieden, in Restaurants zu essen
Zu Hause gearbeitet oder studiert
Zum Arzt gegangen
Arzttermin abgesagt
Einen Vorrat an Medikamenten angelegt
Gebetet

Wie oft überprüfen Sie die Nachrichten über COVID-19?
Noch nie
<1 x / Tag
1 x / Tag
2-4 x / Tag
5-8 x / Tag
9-16 x / Tag
> 16 x / Tag
Wie oft überprüfen Sie soziale Medien auf COVID-19? (z.B. WhatsApp, Facebook)
Noch nie
<1 x / Tag
1 x / Tag
2-4 x / Tag
5-8 x / Tag
9-16 x / Tag
> 16 x / Tag
Wie oft diskutieren Sie COVID-19 in der Massenkommunikation? (z.B. WhatsApp-Gruppe, Twitter)
Noch nie
<1 x / Tag
1 x / Tag
2-4 x / Tag

5-8 x / Tag
9-16 x / Tag
> 16 x / Tag
Wie oft besprechen Sie COVID-19 mit einer anderen Person?
Noch nie
<1 x / Tag
1 x / Tag
2-4 x / Tag
5-8 x / Tag
9-16 x / Tag
> 16 x / Tag

Wie besorgt sind Sie über COVID-19?
(***list not transferred)

Was macht Ihnen bei Covid-19 am meisten Sorgen? Wählen Sie alle zutreffenden Antworten:
Dass ich COVID-19 bekomme und die Infektion nach Hause bringe
Dass mein Partner COVID-19 bekommt und die Infektion nach Hause bringt
Dass meine Familienmitglieder / Freunde mit COVID-19 infiziert werden.
Dass die COVID-19 Pandemie meine wirtschaftliche Situation / Finanzen erheblich beeinflusst (zum Beispiel, dass ich meinen Job verliere)
Dass mein ungeborenes Baby COVID-19 bekommt
Dass COVID-19 Änderungen in meinem Geburtsplan verursacht
Dass mein Partner/ meine Hilfsperson aufgrund von COVID-19 während der Geburt nicht bei mir sein kann
Dass meine Familie mich und das Baby nach der Entbindung aufgrund von Maßnahmen zur Verhinderung der Ausbreitung von COVID-19 nicht besuchen kann
Dass meine anderen Kinder COVID-19 bekommen
Dass meine Eltern / Großeltern das Baby wegen Maßnahmen zur Beendigung von COVID-19 nicht besuchen können.
Dass ich wegen COVID-19 nicht stillen kann
Dass ich meinen anderen Kindern keine angemessene Kinderbetreuung bieten kann
Dass ich nicht an der Beerdigung eines Familienmitglieds teilnehmen kann
Arzttermine verpassen / absagen
Dass ich keine Babyparty oder andere Babyfeier mit Familie oder Freunden haben kann

Andere

Inwieweit hat sich COVID-19 negativ auf die folgenden Bereiche Ihres Lebens ausgewirkt?
Schlaf
Ganz und gar nicht
Ein wenig
Mäßig
Viel
Ernährung
Ganz und gar nicht
Ein wenig
Mäßig
Viel
Fitness
Ganz und gar nicht
Ein wenig
Mäßig
Viel
Arbeit
Ganz und gar nicht
Ein wenig
Mäßig
Viel
Finanzen
Ganz und gar nicht
Ein wenig
Mäßig
Viel
Familie
Ganz und gar nicht
Ein wenig
Mäßig
Viel

Beziehungen
Ganz und gar nicht
Ein wenig
Mäßig
Viel
Religiöse Gebote
Ganz und gar nicht
Ein wenig
Mäßig
Viel
Charakter (z.B. Geduld, Vertrauen)
Ganz und gar nicht
Ein wenig
Mäßig
Viel

Wie oft ist Ihnen seit Beginn der COVID-19 -Pandemie eines der folgenden Dinge passiert?
Sie wurden weniger höflich behandelt als andere Menschen
Fast jeden Tag
Mindestens einmal die Woche
Ein paar Mal im Monat
Noch nie
Sie haben in Restaurants und Geschäften einen schlechteren Service erhalten als andere Leute
Fast jeden Tag
Mindestens einmal die Woche
Ein paar Mal im Monat
Noch nie
Leute haben sich so verhalten, als hätten sie Angst vor Ihnen
Fast jeden Tag
Mindestens einmal die Woche
Ein paar Mal im Monat
Noch nie
Sie wurden beschimpft oder beleidigt

Fast jeden Tag
Mindestens einmal die Woche
Ein paar Mal im Monat
Noch nie
Sie wurden bedroht oder belästigt
Fast jeden Tag
Mindestens einmal die Woche
Ein paar Mal im Monat
Noch nie
Sie wurden körperlich angegriffen
Fast jeden Tag
Mindestens einmal die Woche
Ein paar Mal im Monat
Noch nie

Was ist Ihrer Meinung nach der Hauptgrund für diese Erfahrungen? (Wählen Sie alle, die zutreffen)
Ihre Abstammung oder nationale Herkunft
Ihr Geschlecht
Ihre Rasse oder ethnische Zugehörigkeit
Ihr Alter
Ihre Religion
Ihre Körpergröße
Ihr Gewicht
Ihre Schwangerschaft
Ihre sexuelle Orientierung
Ihr Bildungs- oder Einkommensniveau
Andere

Bitte beantworten Sie die folgenden Fragen basierend auf den letzten 2 Wochen.
Wie oft hatten Sie das Gefühl, dass Sie die wichtigen Dinge in Ihrem Leben nicht kontrollieren konnten?
Noch nie
Fast nie
Manchmal

Ziemlich oft
Sehr oft
Wie oft waren Sie davon überzeugt, dass Sie mit Ihren persönlichen Problemen fertig werden?
Noch nie
Fast nie
Manchmal
Ziemlich oft
Sehr oft
Wie oft hatten Sie das Gefühl, dass alles in die richtige Richtung geht?
Noch nie
Fast nie
Manchmal
Ziemlich oft
Sehr oft
Wie oft hatten Sie das Gefühl, dass sich die Schwierigkeiten so stark häuften, dass Sie sie nicht überwinden konnten?
Noch nie
Fast nie
Manchmal
Ziemlich oft
Sehr oft

Wie oft wurden Sie in den letzten 2 Wochen von den folgenden Problemen heimgesucht?
Sie fühlten sich nervös, ängstlich oder gereizt
Ganz und gar nicht
Einige Tage
Mehr als die Hälfte der Tage
Fast jeden Tag
Sie waren nicht in der Lage, Ihre Sorgen zu stoppen oder zu kontrollieren
Ganz und gar nicht
Einige Tage
Mehr als die Hälfte der Tage
Fast jeden Tag
Sie fühlten sich niedergeschlagen, depressiv oder hoffnungslos

Ganz und gar nicht
Einige Tage
Mehr als die Hälfte der Tage
Fast jeden Tag
Sei hatten wenig Interesse oder Freude daran, Dinge zu tun
Ganz und gar nicht
Einige Tage
Mehr als die Hälfte der Tage
Fast jeden Tag

Wie oft essen Sie seit Beginn der COVID-19 -Krise ...
Weil Sie depressiv oder traurig sind
Fast nie oder nie
Selten
Manchmal
Häufig
Fast immer oder immer
Um die Situation zu bewältigen
Fast nie oder nie
Selten
Manchmal
Häufig
Fast immer oder immer
Um sich zu trösten
Fast nie oder nie
Selten
Manchmal
Häufig
Fast immer oder immer
Weil Sie sich wertlos oder unzulänglich fühlen
Fast nie oder nie
Selten
Manchmal

Häufig
Fast immer oder immer
Um zu vermeiden, an etwas Unangenehmes zu denken oder sich abzulenken
Fast nie oder nie
Selten
Manchmal
Häufig
Fast immer oder immer

Geben Sie an, inwieweit Sie den folgenden Aussagen zustimmen
Ich habe so viel im Leben, wofür ich dankbar sein kann
Widerspreche entschieden
Stimme nicht zu
Stimme nicht ganz zu
Stimme weder zu noch widerspreche
Stimme etwas zu
Stimme zu
Stimme voll und ganz zu
Wenn ich alles auflisten müsste, wofür ich dankbar war, wäre es eine sehr lange Liste
Widerspreche entschieden
Stimme nicht zu
Stimme nicht ganz zu
Stimme weder zu noch widerspreche
Stimme etwas zu
Stimme zu
Stimme voll und ganz zu
Ich bin einer Vielzahl von Menschen dankbar
Widerspreche entschieden
Stimme nicht zu
Stimme nicht ganz zu
Stimme weder zu noch widerspreche
Stimme etwas zu
Stimme zu

Stimme voll und ganz zu

Bitte beschreiben Sie, wie sich die COVID-19 -Krise am meisten auf Sie ausgewirkt hat:

Wie unterstützend sind die folgenden Personen seit Beginn der COVID-19 -Krise?
Ehemann oder Lebensgefährte
Ganz und gar nicht
Ein kleines bisschen
Mäßig
Ziemlich
Sehr
N/A
Eltern oder Erziehungsberechtigte
Ganz und gar nicht
Ein kleines bisschen
Mäßig
Ziemlich
Sehr
N/A
Kinder
Ganz und gar nicht
Ein kleines bisschen
Mäßig
Ziemlich
Sehr
N/A
Geschwister
Ganz und gar nicht
Ein kleines bisschen
Mäßig
Ziemlich
Sehr
N/A

FREUNDE
Ganz und gar nicht
Ein kleines bisschen
Mäßig
Ziemlich
Sehr
N/A
Mitarbeiter
Ganz und gar nicht
Ein kleines bisschen
Mäßig
Ziemlich
Sehr
N/A

Wie gestresst fühlen Sie sich seit Beginn der COVID-19 -Krise von den folgenden Personen?
Ehemann oder Lebensgefährte
Ganz und gar nicht
Ein kleines bisschen
Mäßig
Ziemlich
Sehr
N/A
Eltern oder Erziehungsberechtigte
Ganz und gar nicht
Ein kleines bisschen
Mäßig
Ziemlich
Sehr
N/A
Kinder
Ganz und gar nicht
Ein kleines bisschen

Mäßig
Ziemlich
Sehr
N/A
Geschwister
Ganz und gar nicht
Ein kleines bisschen
Mäßig
Ziemlich
Sehr
N/A
FREUNDE
Ganz und gar nicht
Ein kleines bisschen
Mäßig
Ziemlich
Sehr
N/A
Mitarbeiter
Ganz und gar nicht
Ein kleines bisschen
Mäßig
Ziemlich
Sehr
N/A

Seit Beginn der COVID-19-Krise…
Wie oft haben Sie das Gefühl, dass Ihnen die Gesellschaft fehlt?
Fast nie
Einen Teil der Zeit
Häufig
Wie oft fühlen Sie sich ausgeschlossen?
Fast nie

Einen Teil der Zeit
Häufig
Wie oft fühlen Sie sich von anderen isoliert?
Fast nie
Einen Teil der Zeit
Häufig

Wenn Sie über Ihre Erfahrungen in den vergangenen 7 Tagen nachdenken, geben Sie bitte an, wie stark Sie den folgenden Aussagen zustimmen oder nicht zustimmen
Ich fühle mich nicht zu etwas gehörig, das ich eine Gemeinschaft nennen würde
Stimme voll und ganz zu
Stimme zu
Stimme etwas zu
Stimme weder zu noch widerspreche
Stimme nicht ganz zu
Stimme nicht zu
Widerspreche entschieden
Ich habe der Welt etwas Wertvolles zu geben
Stimme voll und ganz zu
Stimme zu
Stimme etwas zu
Stimme weder zu noch widerspreche
Stimme nicht ganz zu
Stimme nicht zu
Widerspreche entschieden
Ich fühle mich anderen Menschen in meiner Gemeinschaft nahe
Stimme voll und ganz zu
Stimme zu
Stimme etwas zu
Stimme weder zu noch widerspreche
Stimme nicht ganz zu
Stimme nicht zu
Widerspreche entschieden

Ich kann nicht verstehen, was in der Welt vor sich geht
Stimme voll und ganz zu
Stimme zu
Stimme etwas zu
Stimme weder zu noch widerspreche
Stimme nicht ganz zu
Stimme nicht zu
Widerspreche entschieden
Meine Gemeinschaft ist eine Quelle des Trostes
Stimme voll und ganz zu
Stimme zu
Stimme etwas zu
Stimme weder zu noch widerspreche
Stimme nicht ganz zu
Stimme nicht zu
Widerspreche entschieden
Ich glaube, dass die Leute nett sind
Stimme voll und ganz zu
Stimme zu
Stimme etwas zu
Stimme weder zu noch widerspreche
Stimme nicht ganz zu
Stimme nicht zu
Widerspreche entschieden

Bitte beantworten Sie die folgenden Fragen basierend auf den letzten 7 Tagen
Ich fühle mich aufmerksam oder wachsam
Ganz und gar nicht
Ein kleines bisschen
Mäßig
Ziemlich
Sehr
Andere Dinge lassen mich immer wieder über COVID- 19 nachdenken.

Ganz und gar nicht
Ein kleines bisschen
Mäßig
Ziemlich
Sehr
Ich bin mir bewusst, dass ich viele Gefühle in Zusammenhang mit COVID-19 habe, aber ich beschäftige mich nicht damit
Ganz und gar nicht
Ein kleines bisschen
Mäßig
Ziemlich
Sehr
Ich versuche nicht an COVID-19 zu denken
Ganz und gar nicht
Ein kleines bisschen
Mäßig
Ziemlich
Sehr
Ich habe Probleme mich zu konzentrieren
Ganz und gar nicht
Ein kleines bisschen
Mäßig
Ziemlich
Sehr

Bitte geben Sie uns Feedback zu dieser Umfrage:

Wie haben Sie von uns erfahren?
Google
Facebook-Werbung
Preregistry Facebook-Site
Harvard Webseite
LinkedIn
Ein Post eines Freundes in den sozialen Medien

Mundpropaganda

Spanish Pregistry survey

¿En qué país vive?
(***list of all countries)

¿En qué estado/provincia vive?

¿En qué ciudad vive?

¿Está embarazada?
Sí
No

¿Cuántas semanas de embarazo tiene?
(***list from 5 to 43)

¿Hace cuánto tiempo dio a luz?
Hace menos de un mes
Hace dos meses
Hece tres meses
Hece cuatro meses
Hece cinco meses

¿Cuantos años tiene?
(***list from 18 to 50)

¿Con qué raza(s) se identifica? (marque todo lo que corresponda)
Blanca/Caucásica
Latina/Hispana
Asiática
Del sur de Asia
Negra
De Oriente Medio

Nativa de Hawai u otra isla del Pacífico
India Americana o Nativa de Alaska
Otra/Multirracial

¿Cuál es su estado civil?
Soltera
Casada
Viviendo con una pareja
Separada
Divorciada
Viuda

¿Cuántas personas viven en su hogar (incluida usted)?
(***list not transferred)

¿Cuál es el nivel más alto de educación que alcanzó?
Nunca asistí a la escuela
Escuela primaria
Algo de escuela secundaria
Diploma de escuela secundaria o diploma de equivalencia general (GED)
Algo de Universidad
Diploma Universitario
Maestría
Titulo professional
Doctorado

Indique si durante la pandemia de COVID-19 usted:
Fueron trabajadores de la salud en un hospital o clínica
Trabajó en un hogar de ancianos
Eran un trabajador esencial / clave (según lo definido por el gobierno)
Ninguno de esos
Ninguno de esos

¿Tiene seguro médico a través de su gobierno, su empleador o un miembro de su familia?
(***list not transferred)

¿Le han realizado pruebas de SARS-CoV-2 (el virus que causa COVID-19)? Si es así, ¿cuál fue el resultado?
Positivo, tuve el virus
Negativo, no tenía el virus
Sí, pero todavía no sé el resultado o el resultado no fue concluyente
No, no me han hecho la prueba

¿Le ha diagnosticado un profesional de la salud (por ejemplo, un médico, una enfermera) que tiene COVID-19 basándose solo en sus síntomas?
(***list not transferred)

¿Qué ha hecho en los últimos 7 días para mantenerse usted y otros a salvo del COVID-19? (Seleccione todas las que correspondan)
Usé una mascara
Me lavé las manos con jabón o desinfectante de manos varias veces por día
Desinfecté superficies a mi alrededor
Almacené toallitas desinfectantes o desinfectantes para manos
Almacené alimentos o agua
Cancelé o pospuse viajes aéreos para el trabajo
Cancelé o pospuse viajes aéreos de placer
Cancelé o pospuse actividades laborales o escolares
Cancelé o pospuse actividades personales o sociales
Evité contacto con personas que pueden ser de alto riesgo
Evité espacios públicos, reuniones o multitudes
Evité comer en restaurantes
Trabajé o estudié en casa
Visité un medico
Cancelé una cita con el medico
Almacené medicamentos
Recé

¿Con qué frecuencia revisa las noticias sobre COVID-19?

Nunca
<1 x / día
1 x / día
2-4 x / día
5-8 x / día
9-16 x / día
> 16 x / día

¿Con qué frecuencia revisa redes sociales sobre COVID-19? (por ejemplo, WhatsApp, Facebook)

Nunca
<1 x / día
1 x / día
2-4 x / día
5-8 x / día
9-16 x / día
> 16 x / día

¿Con qué frecuencia discute COVID-19 en comunicaciones masivas? (por ejemplo, en un grupo de WhatsApp, Twitter)

Nunca
<1 x / día
1 x / día
2-4 x / día
5-8 x / día
9-16 x / día
> 16 x / día

¿Con qué frecuencia discute COVID-19 con otra persona?

Nunca
<1 x / día
1 x / día
2-4 x / día
5-8 x / día
9-16 x / día
> 16 x / día

¿Qué tan preocupada está por el COVID-19?

Muy preocupada
Algo preocupada
No muy preocupada
Nada preocupada

¿Qué aspecto del COVID-19 le preocupa más? Seleccione todas las que correspondan:
Que obtendré COVID-19 y traeré la infección a casa
Que mi pareja recibirá COVID-19 y traerá la infección a casa
Que los miembros de mi familia / amigos se infectarán con COVID-19
Que la pandemia COVID-19 afectará significativamente mi situación económica / financiera (por ejemplo, perder mi trabajo)
Que mi bebé nonato tendrá COVID-19
Ese COVID-19 significará cambios en mi plan de entrega
Que mi pareja / persona de apoyo no podrá estar conmigo durante el parto debido a COVID-19
Que mi familia no podrá visitarme a mí y al bebé después del parto debido a medidas para prevenir la propagación de COVID-19
Que mis otros hijos tendrán COVID-19
Que mis padres / abuelos no podrán visitar al bebé debido a medidas para detener COVID-19
Que no podré amamantar debido a COVID-19
Que no podré proporcionar cuidado infantil adecuado para mis otros hijos
Que no podré asistir al funeral de un familiar
Falta / cancelación de citas médicas
Que no podré tener un baby shower u otra celebración de bebé con familiares o amigos
Otro

¿En qué medida el COVID-19 ha impactado negativamente las siguientes áreas de su vida?
Dormir
De ningún modo
Un poco
Moderadamente
Mucho
Dieta
De ningún modo
Un poco
Moderadamente

1291 Mucho
1292 Actividad física
1293 De ningún modo
1294 Un poco
1295 Moderadamente
1296 Mucho
1297 Trabajo
1298 De ningún modo
1299 Un poco
1300 Moderadamente
1301 Mucho
1302 Finanzas
1303 De ningún modo
1304 Un poco
1305 Moderadamente
1306 Mucho
1307 Familia
1308 De ningún modo
1309 Un poco
1310 Moderadamente
1311 Mucho
1312 Relaciones
1313 De ningún modo
1314 Un poco
1315 Moderadamente
1316 Mucho
1317 Observancia religiosa
1318 De ningún modo
1319 Un poco
1320 Moderadamente
1321 Mucho
1322 Carácter (por ejemplo, paciencia, confianza)
1323 De ningún modo

Un poco
Moderadamente
Mucho

Desde que comenzó la pandemia del COVID-19, ¿con qué frecuencia le ha sucedido alguna de las siguientes situaciones?
Fue tratada con menos cortesía que a otras personas
Casi todos los días
Al menos una vez por semana
Algunas veces al mes
Nunca
Recibió peor servicio que otras personas en restaurantes o tiendas
Casi todos los días
Al menos una vez por semana
Algunas veces al mes
Nunca
La gente actúa como si le tuviera miedo
Casi todos los días
Al menos una vez por semana
Algunas veces al mes
Nunca
La insultaron
Casi todos los días
Al menos una vez por semana
Algunas veces al mes
Nunca
La amenazaron o acosaron
Casi todos los días
Al menos una vez por semana
Algunas veces al mes
Nunca
La agredieron físicamente
Casi todos los días
Al menos una vez por semana

Algunas veces al mes
Nunca

¿Cuál cree que es la razón principal de estas experiencias? (Elija todo lo que corresponda)
Tu ascendencia u origen nacional
Tu género
Su raza o etnia
Tu edad
Tu religion
Tu altura
Tu peso
Su embarazo
Tu orientación sexual
Tu nivel educativo o de ingresos
Otro

Responda las siguientes preguntas con respecto a las últimas 2 semanas.
¿Con qué frecuencia se ha sentido incapaz de controlar cosas importantes en su vida?
Nunca
Casi nunca
A veces
Con bastante frecuencia
Muy a menudo
¿Con qué frecuencia se ha sentido segura de su capacidad para manejar sus problemas personales?
Nunca
Casi nunca
A veces
Con bastante frecuencia
Muy a menudo
¿Con qué frecuencia ha sentido que las cosas iban a su manera?
Nunca
Casi nunca
A veces

Con bastante frecuencia
Muy a menudo
¿Con qué frecuencia ha sentido que las dificultades se acumulaban tanto que no podía superarlas?
Nunca
Casi nunca
A veces
Con bastante frecuencia
Muy a menudo

¿Con qué frecuencia la han molestado los siguientes problemas durante las últimas 2 semanas?
Me siento nerviosa o ansiosa
Nunca
Varios días
Más de la mitad de los días
Casi todos los días
No puedo dejar de preocuparme
Nunca
Varios días
Más de la mitad de los días
Casi todos los días
Estoy deprimida o desesperada
Nunca
Varios días
Más de la mitad de los días
Casi todos los días
Tengo poco interés o placer en hacer cosas
Nunca
Varios días
Más de la mitad de los días
Casi todos los días

Desde que comenzó la crisis por el COVID-19, ¿con qué frecuencia come ...
Porque está deprimida o triste

Casi nunca o nunca
Raramente
A veces
A menudo
Casi siempre o siempre
Como una forma de ayudarla a sobrellevar
Casi nunca o nunca
Raramente
A veces
A menudo
Casi siempre o siempre
Como una forma de consolarse
Casi nunca o nunca
Raramente
A veces
A menudo
Casi siempre o siempre
Porque se siente inútil o inadecuada
Casi nunca o nunca
Raramente
A veces
A menudo
Casi siempre o siempre
Como una forma de evitar pensar en algo desagradable o para distraerse
Casi nunca o nunca
Raramente
A veces
A menudo
Casi siempre o siempre

Indique cuánto está de acuerdo con las siguientes declaraciones
Tengo mucho en la vida que agradecer
Muy en desacuerdo

En descauerdo
Algo en desacuerdo
Ni de acuerdo ni en desacuerdo
Parcialmente de acuerdo
De acuerdo
Totalmente de acuerdo
Si tuviera que enumerar todo por lo que estoy agradecida, sería una lista muy larga
Muy en desacuerdo
En descauerdo
Algo en desacuerdo
Ni de acuerdo ni en desacuerdo
Parcialmente de acuerdo
De acuerdo
Totalmente de acuerdo
Estoy agradecida con muchas personas
Muy en desacuerdo
En descauerdo
Algo en desacuerdo
Ni de acuerdo ni en desacuerdo
Parcialmente de acuerdo
De acuerdo
Totalmente de acuerdo

Describa cómo la crisis por el COVID-19 la ha impactado más:

Desde que comenzó la crisis por el COVID-19, ¿qué tan solidarias han sido las siguientes personas?
Marido o pareja
Nada
Un poco
Moderadamente
Bastante
Extremadamente
N / A (no hay tal persona)

1489 Padres o tutores legales
1490 Nada
1491 Un poco
1492 Moderadamente
1493 Bastante
1494 Extremadamente
1495 N / A (no hay tal persona)
1496 Niños/as
1497 Nada
1498 Un poco
1499 Moderadamente
1500 Bastante
1501 Extremadamente
1502 N / A (no hay tal persona)
1503 Hermanos/as
1504 Nada
1505 Un poco
1506 Moderadamente
1507 Bastante
1508 Extremadamente
1509 N / A (no hay tal persona)
1510 Amigos/as
1511 Nada
1512 Un poco
1513 Moderadamente
1514 Bastante
1515 Extremadamente
1516 N / A (no hay tal persona)
1517 Compañeros/as de trabajo
1518 Nada
1519 Un poco
1520 Moderadamente
1521 Bastante

Extremadamente
N / A (no hay tal persona)

Desde que comenzó la crisis por el COVID-19, ¿qué tan estresada se siente por las siguientes personas?
Marido o pareja
Nada
Un poco
Moderadamente
Bastante
Extremadamente
N / A (no hay tal persona)
Padres o tutores legales
Nada
Un poco
Moderadamente
Bastante
Extremadamente
N / A (no hay tal persona)
Niños/as
Nada
Un poco
Moderadamente
Bastante
Extremadamente
N / A (no hay tal persona)
Hermanos/as
Nada
Un poco
Moderadamente
Bastante
Extremadamente
N / A (no hay tal persona)
Amigos/as

Nada
Un poco
Moderadamente
Bastante
Extremadamente
N / A (no hay tal persona)
Compañeros/as de trabajo
Nada
Un poco
Moderadamente
Bastante
Extremadamente
N / A (no hay tal persona)

Desde que comenzó la crisis por el COVID-19...
¿Con qué frecuencia siente que le falta compañía?
Casi nunca
A veces
A menudo
¿Con qué frecuencia se siente excluída?
Casi nunca
A veces
A menudo
¿Con qué frecuencia se siente aislada de los demás?
Casi nunca
A veces
A menudo

Pensando en su experiencia EN LOS ÚLTIMOS 7 DÍAS, indique cuán firmemente está de acuerdo o en desacuerdo con cada una de las siguientes declaraciones
Siento que no pertenezco a nada que llamaría una comunidad
Totalmente de acuerdo
De acuerdo

Parcialmente de acuerdo
Ni de acuerdo ni en desacuerdo
Algo en desacuerdo
Discrepo
Muy en desacuerdo
Tengo algo valioso para darle al mundo
Totalmente de acuerdo
De acuerdo
Parcialmente de acuerdo
Ni de acuerdo ni en desacuerdo
Algo en desacuerdo
Discrepo
Muy en desacuerdo
Me siento cercana a otras personas en mi comunidad.
Totalmente de acuerdo
De acuerdo
Parcialmente de acuerdo
Ni de acuerdo ni en desacuerdo
Algo en desacuerdo
Discrepo
Muy en desacuerdo
No puedo entender lo que está pasando en el mundo
Totalmente de acuerdo
De acuerdo
Parcialmente de acuerdo
Ni de acuerdo ni en desacuerdo
Algo en desacuerdo
Discrepo
Muy en desacuerdo
Mi comunidad es una fuente de Consuelo
Totalmente de acuerdo
De acuerdo
Parcialmente de acuerdo

Ni de acuerdo ni en desacuerdo
Algo en desacuerdo
Discrepo
Muy en desacuerdo
Creo que la gente es amable
Totalmente de acuerdo
De acuerdo
Parcialmente de acuerdo
Ni de acuerdo ni en desacuerdo
Algo en desacuerdo
Discrepo
Muy en desacuerdo

Responda las siguientes preguntas según los últimos 7 días
Me siento vigilante o en guardia
De ningún modo
Un poco
Moderadamente
Bastante
Extremadamente
Otras cosas me siguen haciendo pensar en el COVID-19
De ningún modo
Un poco
Moderadamente
Bastante
Extremadamente
Soy consciente de que tengo muchos sentimientos acerca del COVID-19, pero no los enfrento
De ningún modo
Un poco
Moderadamente
Bastante
Extremadamente
Intento no pensar en el COVID-19

De ningún modo
Un poco
Moderadamente
Bastante
Extremadamente
Tengo problemas para concentrarme
De ningún modo
Un poco
Moderadamente
Bastante
Extremadamente

Por favor, déjenos su comentario acerca de esta encuesta:

¿Cómo se enteró de nosotros?
(***list not transferred)

French Pregistry survey

Dans quel pays vivez-vous ?
(***list of all countries)
Dans quel état/province habitez-vous?

Dans quelle ville habitez-vous ?

Es-tu enceinte?
Oui
Non

De combien de semaines êtes-vous enceinte?
(***list from 5 to 43)

Depuis combien de temps avez-vous accouché?
Il’y a moins d’un mois
Il ya un mois
Il y a deux mois
Il ya trois mois
Il y a quatre mois
Il y a cinq mois

Quel âge avez-vous?
(***list from 18 to 50)

À quelle(s) race(s) vous identifiez-vous le plus ? (Cochez toutes les réponses qui s'appliquent)
Blanc/Caucasien
Latin/Hispanique
Asiatique
Sud-asiatique
Noir
Moyen-Orient

Hawaïen natif ou autre insulaire du Pacifique
Amérindien ou originaire de l’Alaska
Autre/Multirace

Quel est votre état civil?
Célibataire
Mariée
En union libre
Séparée
Divorcée
Veuve

Combien de personnes vivent dans votre ménage (vous y compris)?
1
2
3
4
5
6
7
8
9
10
Plus de 10

Quel est le niveau de scolarité le plus élevé que vous ayez atteint?
N'a jamais fréquenté l'école
Primaire
Secondaire
Diplôme d'études secondaires ou diplôme d'équivalence générale (GED)
Collège/université
Diplôme d'études collégiales ou universitaires
Maîtrise

Diplôme professionnel
Doctorat

Veuillez indiquer si, pendant la pandémie de COVID-19, vous:
Si vous étiez une professionnelle de la santé dans un hôpital ou une Clinique
si vous avez travaillé dans une maison de repos
Si vous étiez une travailleuse essentielle/clé (telle que définie par le gouvernement)
Aucun d'entre eux
Je ne sais pas

Avez-vous une assurance médicale auprès de votre gouvernement, de votre employeur ou d\'un membre de votre famille?
Oui
Non

Avez-vous été testée pour le SRAS-CoV-2 (le virus qui cause COVID-19)? Si oui, quel a été le résultat?
Positif, j'avais le virus
Négatif, je n'avais pas le virus
Oui, mais je ne connais pas encore le résultat ou le résultat n'a pas été concluant
Non, je n'ai pas été testée

Un professionnel de la santé (p. Ex. Un médecin, une infirmière) vous a-t-il diagnostiqué un COVID- 19 uniquement en fonction de vos symptômes?
(***list not transferred)

Avez-vous été en contact avec une personne qui a ou a eu le COVID-19?
(***list not transferred)

Lesquelles des mesures suivantes avez-vous prises au cours des 7 derniers jours pour vous protéger ainsi que les autres contre le COVID-19? (Sélectionnez toutes les réponses correspondantes)
Porter un masque facial
Se laver les mains avec du savon ou un désinfectant pour les mains plusieurs fois par jour
Surfaces désinfectées autour de vous
Lingettes désinfectantes pour les mains

Stock de nourriture ou d'eau
Voyage aérien annulé ou reporté pour le travail
Voyage aérien annulé ou reporté pour le Plaisir
Travail ou activités scolaires annulés ou reportés
Activités personnelles ou sociales annulées ou reportées
Éviter tout contact avec des personnes à haut risqué
Espaces publics, rassemblements ou foules évités
Eviter de manger dans les restaurants
Travailler ou étudier à domicile
Visiter un médecin
Annuler un rendez-vous chez le médecin
Médicaments stockés
Prier

À quelle fréquence consultez-vous les actualités concernant COVID-19?
Jamais
<1 x / jour
1 x / jour
2-4 x / jour
5-8 x / jour
9-16 x / jour
> 16 x / jour
À quelle fréquence consultez-vous les médias sociaux à propos de COVID-19? (par exemple WhatsApp, Facebook)
Jamais
<1 x / jour
1 x / jour
2-4 x / jour
5-8 x / jour
9-16 x / jour
> 16 x / jour
À quelle fréquence discutez-vous de COVID-19 dans les communications de masse? (par exemple, groupe WhatsApp, Twitter)
Jamais
<1 x / jour

1 x / jour
2-4 x / jour
5-8 x / jour
9-16 x / jour
> 16 x / jour
À quelle fréquence discutez-vous de COVID-19 avec une autre personne?
Jamais
<1 x / jour
1 x / jour
2-4 x / jour
5-8 x / jour
9-16 x / jour
> 16 x / jour

Êtes-vous inquiète à propos de COVID-19?
(***list not transferred)

Qu'est ce qui vous inquiète le plus à propos de COVID-19 ? Sélectionnez toutes les réponses correspondantes.
Que je sois infectée par COVID-19 et ramener l'infection à la maison
Que mon partenaire soit infecté par COVID-19 et ramènera l'infection à la maison
Que les membres de ma famille / amis soient infectés par COVID-19
Que la pandémie COVID-19 affecte de manière significative ma situation économique / financière (par exemple, perdre mon emploi)
Que mon bébé à naître soit infecté par COVID-19
Que COVID-19 signifie des changements à mon accouchement programmé
Que mon partenaire / personne de soutien ne puisse pas être avec moi pendant l'accouchement en raison de COVID-19
Que ma famille ne puisse pas me rendre visite à moi et au bébé après l'accouchement en raison de mesures de prévention de la propagation du COVID-19
Que mes autres enfants soient infectés par COVID-19
Que mes parents / grands-parents ne puissent pas rendre visite au bébé en raison de mesures de prévention de la propagation du COVID-19
Que je ne sois pas en mesure d'allaiter à cause du COVID-19
Que je ne sois pas en mesure de fournir des services de garde adéquats à mes autres enfants
Que je ne puisse pas assister aux funérailles d'un membre de la famille

Que je doive manquer/annuler un rendez-vous chez le médecin
Que je ne puisse pas organiser de baby shower ou autre fête de bébé en famille ou entre amis
Autre

Dans quelle mesure COVID-19 a-t-il eu un impact négatif sur les domaines suivants de votre vie?
Dormir
Pas du tout
Un petit peu
Modérément
Beaucoup
Régime
Pas du tout
Un petit peu
Modérément
Beaucoup
Aptitude
Pas du tout
Un petit peu
Modérément
Beaucoup
Travail
Pas du tout
Un petit peu
Modérément
Beaucoup
Finances
Pas du tout
Un petit peu
Modérément
Beaucoup
Famille
Pas du tout
Un petit peu

Modérément
Beaucoup
Relationnelle
Pas du tout
Un petit peu
Modérément
Beaucoup
Observance religieuse
Pas du tout
Un petit peu
Modérément
Beaucoup
Caractère (par exemple, patience, confiance)
Pas du tout
Un petit peu
Modérément
Beaucoup

Depuis le début de la pandémie COVID-19, à quelle fréquence vous est-il arrivé l'une des choses suivantes?
Vous avez été traitée avec moins de courtoisie que les autres
Presque tous les jours
Au moins une fois par semaine
Quelques fois par mois
Jamais
Vous avez reçu un service moins bon que d'autres personnes dans des restaurants ou des magasins
Presque tous les jours
Au moins une fois par semaine
Quelques fois par mois
Jamais
Les gens ont agi comme s'ils avaient peur de vous
Presque tous les jours
Au moins une fois par semaine
Quelques fois par mois

Jamais
Vous avez été interpellée ou insultée
Presque tous les jours
Au moins une fois par semaine
Quelques fois par mois
Jamais
Vous avez été menacée ou harcelée
Presque tous les jours
Au moins une fois par semaine
Quelques fois par mois
Jamais
Vous avez été agressée physiquement
Presque tous les jours
Au moins une fois par semaine
Quelques fois par mois
Jamais

Selon vous, quelle est la principale raison de ces expériences? (Choisissez tout ce qui correspond)
Votre ascendance ou vos origines nationales
Votre sexe
Votre race ou origine ethnique
Votre âge
Votre religion
Ton poids
Votre poids
Votre grossesse
Votre orientation sexuelle
Votre niveau d'éducation ou de revenue
Autre

Veuillez répondre aux questions suivantes en fonction des 2 dernières semaines.
Combien de fois avez-vous ressenti que vous étiez incapable de contrôler les choses importantes de votre vie ?
Jamais

Presque jamais
Parfois
Assez souvent
Très souvent
À quelle fréquence vous êtes-vous senti confiante quant à votre capacité à gérer vos problèmes personnels ?
Jamais
Presque jamais
Parfois
Assez souvent
Très souvent
Combien de fois avez-vous ressenti que les choses allaient dans votre sens ?
Jamais
Presque jamais
Parfois
Assez souvent
Très souvent
Combien de fois avez-vous ressenti des difficultés qui s'accumulent au point de ne pas pouvoir être surmontées ?
Jamais
Presque jamais
Parfois
Assez souvent
Très souvent

À quelle fréquence avez-vous été gênée par les problèmes suivants au cours des 2 dernières semaines?
Se sentir nerveuse ou anxieuse
Pas du tout
Plusieurs jours
Plus de la moitié des jours
Presque tous les jours
Ne pas pouvoir arrêter ou contrôler l'inquiétude
Pas du tout
Plusieurs jours
Plus de la moitié des jours

Presque tous les jours
Se sentir déprimée ou désespérée
Pas du tout
Plusieurs jours
Plus de la moitié des jours
Presque tous les jours
Peu d'intérêt ou de plaisir à faire les choses
Pas du tout
Plusieurs jours
Plus de la moitié des jours
Presque tous les jours

Depuis le début de la crise du COVID-19, à quelle fréquence mangez-vous ...
Parce que vous êtes déprimée ou triste
Presque jamais ou jamais
Rarement
PARFOIS
Souvent
Presque toujours ou toujours
Pour vous aider à faire face
Presque jamais ou jamais
Rarement
PARFOIS
Souvent
Presque toujours ou toujours
Pour vous réconforter
Presque jamais ou jamais
Rarement
PARFOIS
Souvent
Presque toujours ou toujours
Parce que vous vous sentez inutile ou insuffisante
Presque jamais ou jamais

Rarement
PARFOIS
Souvent
Presque toujours ou toujours
Pour éviter de penser à quelque chose de désagréable ou pour vous distraire
Presque jamais ou jamais
Rarement
PARFOIS
Souvent
Presque toujours ou toujours

Indiquez dans quelle mesure vous êtes d'accord avec les affirmations suivantes
J'ai tellement de choses dans la vie pour lesquelles je suis reconnaissante
Pas du tout d'accord
Vraiment en désaccord
Plutôt en désaccord
Ni d'accord ni en désaccord
Plutôt d'accord
Vraiment d'accord
Tout à fait d'accord
Si je devais énumérer tout ce dont je me sentais reconnaissante, ce serait une très longue liste
Pas du tout d'accord
Vraiment en désaccord
Plutôt en désaccord
Ni d'accord ni en désaccord
Plutôt d'accord
Vraiment d'accord
Tout à fait d'accord
Je suis reconnaissante envers une grande variété de personnes
Pas du tout d'accord
Vraiment en désaccord
Plutôt en désaccord
Ni d'accord ni en désaccord

Plutôt d'accord
Vraiment d'accord
Tout à fait d'accord

Veuillez décrire comment la crise COVID-19 vous a le plus affecté:

Depuis le début de la crise COVID-19, dans quelle mesure les personnes suivantes sont-elles favorables?
Époux ou compagnon
Pas du tout
Un petit peu
Modérément
Un peu
Extrêmement
N / A (Il n'y a pas de telle personne)
Parents ou tuteurs légaux
Pas du tout
Un petit peu
Modérément
Un peu
Extrêmement
N / A (Il n'y a pas de telle personne)
Enfants
Pas du tout
Un petit peu
Modérément
Un peu
Extrêmement
N / A (Il n'y a pas de telle personne)
Fratrie
Pas du tout
Un petit peu
Modérément
Un peu

Extrêmement
N / A (Il n'y a pas de telle personne)
Copains
Pas du tout
Un petit peu
Modérément
Un peu
Extrêmement
N / A (Il n'y a pas de telle personne)
Collègues de travail
Pas du tout
Un petit peu
Modérément
Un peu
Extrêmement
N / A (Il n'y a pas de telle personne)

Depuis le début de la crise COVID-19, à quel point vous sentez-vous stressée par les personnes suivantes?
Époux ou compagnon
Pas du tout
Un petit peu
Modérément
Un peu
Extrêmement
N / A (Il n'y a pas de telle personne)
Parents ou tuteurs légaux
Pas du tout
Un petit peu
Modérément
Un peu
Extrêmement
N / A (Il n'y a pas de telle personne)
Enfants

Pas du tout
Un petit peu
Modérément
Un peu
Extrêmement
N / A (Il n'y a pas de telle personne)
Fratrie
Pas du tout
Un petit peu
Modérément
Un peu
Extrêmement
N / A (Il n'y a pas de telle personne)
Copains
Pas du tout
Un petit peu
Modérément
Un peu
Extrêmement
N / A (Il n'y a pas de telle personne)
Collègues de travail
Pas du tout
Un petit peu
Modérément
Un peu
Extrêmement
N / A (Il n'y a pas de telle personne)

Depuis le début de la crise COVID-19…
À quelle fréquence sentez-vous que vous manquez de compagnie?
Presque jamais
Une partie du temps
Souvent

À quelle fréquence vous sentez-vous exclue ?
Presque jamais
Une partie du temps
Souvent
À quelle fréquence vous sentez-vous isolée des autres?
Presque jamais
Une partie du temps
Souvent

En pensant à votre expérience AU COURS DES 7 DERNIERS JOURS, veuillez indiquer dans quelle mesure vous êtes d'accord ou en désaccord avec chacune des affirmations suivantes
Je n'ai pas l'impression d'appartenir à quelque chose que j'appellerais une communauté
Tout à fait d'accord
Vraiment d'accord
Plutôt d'accord
Ni d'accord ni en désaccord
Plutôt en désaccord
Être en désaccord
Pas du tout d'accord
J'ai quelque chose de précieux à donner au monde
Tout à fait d'accord
Vraiment d'accord
Plutôt d'accord
Ni d'accord ni en désaccord
Plutôt en désaccord
Être en désaccord
Pas du tout d'accord
Je me sens proche des autres personnes de ma communauté
Tout à fait d'accord
Vraiment d'accord
Plutôt d'accord
Ni d'accord ni en désaccord
Plutôt en désaccord

Être en désaccord
Pas du tout d'accord
Je ne peux pas comprendre ce qui se passe dans le monde
Tout à fait d'accord
Vraiment d'accord
Plutôt d'accord
Ni d'accord ni en désaccord
Plutôt en désaccord
Être en désaccord
Pas du tout d'accord
Ma communauté est une source de réconfort
Tout à fait d'accord
Vraiment d'accord
Plutôt d'accord
Ni d'accord ni en désaccord
Plutôt en désaccord
Être en désaccord
Pas du tout d'accord
Je crois que les gens sont gentils
Tout à fait d'accord
Vraiment d'accord
Plutôt d'accord
Ni d'accord ni en désaccord
Plutôt en désaccord
Être en désaccord
Pas du tout d'accord

Veuillez répondre aux questions suivantes en fonction des 7 derniers jours
Je me sens vigilante
Pas du tout
Un petit peu
Modérément
Un peu

Extrêmement
D'autres choses me font penser à COVID-19
Pas du tout
Un petit peu
Modérément
Un peu
Extrêmement
Je suis consciente que j'ai beaucoup de préoccupations concernant le COVID-19, mais je ne m'en occupe pas
Pas du tout
Un petit peu
Modérément
Un peu
Extrêmement
J'essaie de ne pas penser à COVID-19
Pas du tout
Un petit peu
Modérément
Un peu
Extrêmement
J'ai du mal à me concentrer
Pas du tout
Un petit peu
Modérément
Un peu
Extrêmement

Veuillez fournir vos commentaires sur cette enquête:

Comment avez-vous entendu parler de nous?
(***list not transferred)

Italian Pregistry survey

In quale nazione vivi?
(***list of all countries)

In quale regione/provincia vivi?

In quale città vivi?

Sei incinta?
Sì
No

Di quante settimane sei incinta?
(***list from 5 to 43)

Quanti anni hai?
(***list from 18 to 50)

Con quale razza ti identifichi di più? (controlla tutte le opzioni)
Bianca/ Caucasica
Latina / Ispanica
Asiatica
Sud asiatica
Nera
Medio orientale
Nativa Hawaiiana o di un'altra isola del Pacifico
Indiano americana o nativa dell’Alaska
Altra/multirazziale

Qual è il tuo Stato civile?
Single
Sposata

Convivente
Separata
Divorziata
Vedova

Quante persone vivono nella tua famiglia (incluso te stesso)?
(***list not transferred)

Qual è il più alto livello di istruzione che hai raggiunto?
Mai frequentato la scuola
Scuola elementare
Scuola media inferior
Scuola media superior
Università
Diploma universitario o titolo Universitario
Master
Titolo professionale
Dottorato

Ti invitiamo ad indicare se durante la pandemia COVID-19:
Sei stata un operatore sanitario in un ospedale o in una clinica
Hai lavorato in una casa di cura
Sei stata lavoratore essenziale / chiave (come definito dal governo)
Nessuna di queste
Non lo so

Avete un\'assicurazione medica attraverso il vostro governo, il vostro datore di lavoro o un familiare?
(***list not transferred)

Sei stata testata per SARS-CoV-2 (il virus che causa COVID-19)? In tal caso, qual è stato il risultato?
Positivo, ho avuto il virus
Negativo, non avevo il virus
Sì, ma non conosco ancora il risultato o il risultato è stato inconcludente

No, non sono stata testata

Un professionista sanitario (ad es. Medico, infermiere) ti ha diagnosticato COVID- 19 solo in base ai tuoi sintomi?
(***list not transferred)

Sei stata a contatto con una persona che ha o ha avuto COVID-19?
(***list not transferred)

Quale delle seguenti operazioni hai attuato negli ultimi 7 giorni per proteggere te stessa e gli altri da COVID- 19? (Seleziona tutte le risposte pertinenti)
Ho indossato una Maschera
Mi sono lavata le mani con sapone o disinfettante usato più volte al giorno
Ho disinfettato le superfici intorno a me
Ho utilizzato salviette disinfettanti per le mani o disinfettanti
Ho fatto scorta di cibo o acqua
Viaggio aereo annullato o posticipato per lavoro
Viaggio aereo annullato o posticipato per piacere
Attività lavorative o scolastiche annullate o posticipate
Attività personali o sociali annullate o posticipate
Ho evitato il contatto con persone che potrebbero essere ad alto rischio
Ho evitato spazi pubblici, raduni o folle
Ho evitato di mangiare nei ristorante
Ha lavorato o studiato a casa
Ho visitato un dottore
Ho annullato un appuntamento dal medico
Ho fatto scorta di farmaci
Ho pregato

Con quale frequenza controlli le notizie sul COVID-19
Mai
<1 al giorno
1 al giorno
2-4 al giorno

5-8 al giorno
9-16 al giorno
> 16 al giorno
Con quale frequenza controlli i social media sul COVID-19? (ad es. WhatsApp, Facebook)
Mai
<1 al giorno
1 al giorno
2-4 al giorno
5-8 al giorno
9-16 al giorno
> 16 al giorno
Quanto spesso parli di COVID-19 nelle comunicazioni di massa? (ad es. gruppo WhatsApp, Twitter)
Mai
<1 al giorno
1 al giorno
2-4 al giorno
5-8 al giorno
9-16 al giorno
> 16 al giorno
Con quale frequenza parli del COVID-19 con un'altra persona?
Mai
<1 al giorno
1 al giorno
2-4 al giorno
5-8 al giorno
9-16 al giorno
> 16 al giorno

Quanto sei preoccupata per il COVID-19?
Molto preoccupata
Un po ‘preoccupata
Non molto preoccupata
Per niente preoccupata

Cosa ti preoccupa di più del COVID-19? Seleziona tutto ciò che si applica:
Che prenderò il COVID-19 e lo trasmetterò a quelli che vivono con me
Che il mio partner prenderà il COVID-19 e lo porterà in casa
Che i miei familiari / amici saranno infettati da COVID-19
Che la pandemia COVID-19 influenzerà in modo significativo la mia situazione economica / le mie finanze (ad esempio, perdere il lavoro)
Che trasmetterò il COVID-19 al mio bambino non ancora nato
Che il COVID-19 comporterà modifiche ai miei pani per il parto
Che il mio partner / persona di supporto non sarà in grado di stare con me durante il parto a causa del COVID-19
Che la mia famiglia non sarà in grado di visitare me e il bambino dopo il parto a causa delle misure per prevenire la diffusione del COVID-19
Che i miei altri figli prenderanno il COVID-19
Che i miei genitori / nonni non saranno in grado di visitare il bambino a causa delle misure per fermare la diffusione del COVID-19
Che non sarò in grado di allattare a causa di COVID-19
Che non sarò in grado di fornire un'adeguata assistenza agli altri miei figli
Che non potrò partecipare al funerale di un membro della famiglia
Mancare / annullare gli appuntamenti del medico
Che non sarò in grado di fare una festa prémaman o altre feste per il bambino con la famiglia o gli amici
Altro

In che misura il COVID-19 ha avuto un impatto negativo sulle seguenti aree della tua vita?
Sonno
Affatto
Un po’
Moderatamente
Molto
Dieta
Affatto
Un po’
Moderatamente
Molto
Fitness

Affatto
Un po’
Moderatamente
Molto
Lavoro
Affatto
Un po’
Moderatamente
Molto
Finanza
Affatto
Un po’
Moderatamente
Molto
Famiglia
Affatto
Un po’
Moderatamente
Molto
Relazione
Affatto
Un po’
Moderatamente
Molto
Osservanza religiosa
Affatto
Un po’
Moderatamente
Molto
Carattere (ad es. pazienza, fiducia)
Affatto
Un po’
Moderatamente

Molto

Dall'inizio della pandemia COVID-19, con quale frequenza ti è successa una delle seguenti cose?
Sei stata trattata con meno cortesia delle altre persone
Quasi ogni giorno
Almeno una volta a settimana
Alcune volte al mese
Mai
Hai ricevuto un servizio peggiore rispetto ad altre persone in ristoranti o negozi
Quasi ogni giorno
Almeno una volta a settimana
Alcune volte al mese
Mai
Le persone si sono comportate come se avessero paura di te
Quasi ogni giorno
Almeno una volta a settimana
Alcune volte al mese
Mai
Sei stata insultata
Quasi ogni giorno
Almeno una volta a settimana
Alcune volte al mese
Mai
Sei stata minacciata o molestata
Quasi ogni giorno
Almeno una volta a settimana
Alcune volte al mese
Mai
Sei stata aggredita fisicamente
Quasi ogni giorno
Almeno una volta a settimana
Alcune volte al mese
Mai

Quale pensi sia la ragione principale di queste esperienze? (Scegliere tutte le opzioni pertinenti)
La tua origine o origini nazionali
Il tuo genere
La tua razza o etnia
Della tua età
La tua religione
La vostra altezza
Il tuo peso
La tua gravidanza
Il tuo orientamento sessuale
La tua istruzione o livello di reddito
Altro

Rispondi alle seguenti domande in base alle ultime 2 settimane.
Quante volte hai sentito di non essere in grado di controllare le cose importanti della tua vita?
Mai
Quasi mai
A volte
Abbastanza spesso
Molto spesso
Con quale frequenza ti sei sentita sicura della tua capacità di gestire i tuoi problemi personali?
Mai
Quasi mai
A volte
Abbastanza spesso
Molto spesso
Quante volte hai sentito che le cose stavano andando per il verso giusto?
Mai
Quasi mai
A volte
Abbastanza spesso
Molto spesso

Quante volte hai sentito che le difficoltà erano così tante da non poterle sormontare?
Mai
Quasi mai
A volte
Abbastanza spesso
Molto spesso

Con quale frequenza hai affrontato i seguenti problemi nelle ultime 2 settimane?
Sensazione di nervosismo, ansia o tensione
Affatto
Diversi giorni
Più della metà dei giorni
Quasi ogni giorno
Non essere in grado di eliminare o controllare la preoccupazione
Affatto
Diversi giorni
Più della metà dei giorni
Quasi ogni giorno
Sentirsi giù, depressa o senza Speranza
Affatto
Diversi giorni
Più della metà dei giorni
Quasi ogni giorno
Poco interesse o piacere nel fare le cose
Affatto
Diversi giorni
Più della metà dei giorni
Quasi ogni giorno

Dall'inizio della crisi COVID-19, quanto spesso mangi ...
Perché sei depressa o triste
Quasi mai o mai
Raramente

A volte
Spesso
Quasi sempre o sempre
per affrontare la preoccupazione
Quasi mai o mai
Raramente
A volte
Spesso
Quasi sempre o sempre
Per confortarti
Quasi mai o mai
Raramente
A volte
Spesso
Quasi sempre o sempre
Perché ti senti inutile o inadeguata
Quasi mai o mai
Raramente
A volte
Spesso
Quasi sempre o sempre
Come un modo per evitare di pensare a qualcosa di spiacevole o di distrarti
Quasi mai o mai
Raramente
A volte
Spesso
Quasi sempre o sempre

Indica quanto sei d'accordo con le seguenti dichiarazioni
Ho così tanto nella vita di cui essere grata
In forte disaccordo
Disaccordo
In qualche modo in disaccordo

Né d'accordo né in disaccordo
Abbastanza d'accordo
D’accordo
Assolutamente d’accordo
Se dovessi elencare tutto ciò di cui sono grata, sarebbe un elenco molto lungo
In forte disaccordo
Disaccordo
In qualche modo in disaccordo
Né d'accordo né in disaccordo
Abbastanza d'accordo
D’accordo
Assolutamente d’accordo
Sono grata a moltissime persone
In forte disaccordo
Disaccordo
In qualche modo in disaccordo
Né d'accordo né in disaccordo
Abbastanza d'accordo
D’accordo
Assolutamente d’accordo

Descrivi in che modo la crisi COVID-19 ti ha colpito di più:

Dall'inizio della crisi COVID-19, quanto sono di supporto le seguenti persone?
Marito o altro significativo
Affatto
Un po
Moderatamente
Un bel po
Estremamente
N / A (non esiste una persona simile)
Genitori o tutori legali
Affatto

Un po
Moderatamente
Un bel po
Estremamente
N / A (non esiste una persona simile)
Bambini
Affatto
Un po
Moderatamente
Un bel po
Estremamente
N / A (non esiste una persona simile)
Fratelli
Affatto
Un po
Moderatamente
Un bel po
Estremamente
N / A (non esiste una persona simile)
Amici
Affatto
Un po
Moderatamente
Un bel po
Estremamente
N / A (non esiste una persona simile)
Collaboratori
Affatto
Un po
Moderatamente
Un bel po
Estremamente
N / A (non esiste una persona simile)

Dall'inizio della crisi COVID-19, quanto ti senti stressata dalle seguenti persone?
Marito o altro significativo
Affatto
Un po
Moderatamente
Un bel po
Estremamente
N / A (non esiste una persona simile)
Genitori o tutori legali
Affatto
Un po
Moderatamente
Un bel po
Estremamente
N / A (non esiste una persona simile)
Bambini
Affatto
Un po
Moderatamente
Un bel po
Estremamente
N / A (non esiste una persona simile)
Fratelli
Affatto
Un po
Moderatamente
Un bel po
Estremamente
N / A (non esiste una persona simile)
Amici
Affatto
Un po

Moderatamente
Un bel po
Estremamente
N / A (non esiste una persona simile)
Collaboratori
Affatto
Un po
Moderatamente
Un bel po
Estremamente
N / A (non esiste una persona simile)

Dall'inizio della crisi COVID-19...
Quanto spesso senti di non avere compagnia?
Quasi mai
Qualche volta
Spesso
Quanto spesso ti senti esclusa?
Quasi mai
Qualche volta
Spesso
Quanto spesso ti senti isolata dagli altri?
Quasi mai
Qualche volta
Spesso

Pensando alla tua esperienza NEGLI ULTIMI 7 GIORNI, indica in che misura sei d'accordo o in disaccordo con ciascuna delle seguenti affermazioni
Non mi sento di appartenere ad una comunità
Sono assolutamente d'accordo
Sono d'accordo
Abbastanza d'accordo
Né d'accordo né in disaccordo

2691 In qualche modo in disaccordo
2692 In disaccordo
2693 In forte disaccordo
2694 Ho qualcosa di prezioso da dare al mondo
2695 Sono assolutamente d'accordo
2696 Sono d'accordo
2697 Abbastanza d'accordo
2698 Né d'accordo né in disaccordo
2699 In qualche modo in disaccordo
2700 In disaccordo
2701 In forte disaccordo
2702 Mi sento vicino alle altre persone nella mia comunità
2703 Sono assolutamente d'accordo
2704 Sono d'accordo
2705 Abbastanza d'accordo
2706 Né d'accordo né in disaccordo
2707 In qualche modo in disaccordo
2708 In disaccordo
2709 In forte disaccordo
2710 Non riesco a dare un senso a quello che sta succedendo nel mondo
2711 Sono assolutamente d'accordo
2712 Sono d'accordo
2713 Abbastanza d'accordo
2714 Né d'accordo né in disaccordo
2715 In qualche modo in disaccordo
2716 In disaccordo
2717 In forte disaccordo
2718 La mia comunità è fonte di conforto
2719 Sono assolutamente d'accordo
2720 Sono d'accordo
2721 Abbastanza d'accordo
2722 Né d'accordo né in disaccordo
2723 In qualche modo in disaccordo

In disaccordo
In forte disaccordo
Credo che le persone siano gentili
Sono assolutamente d'accordo
Sono d'accordo
Abbastanza d'accordo
Né d'accordo né in disaccordo
In qualche modo in disaccordo
In disaccordo
In forte disaccordo

Rispondi alle seguenti domande in base agli ultimi 7 giorni
Mi sento vigile o in guardia
Affatto
Un po’
Moderatamente
Molto
Estremamente
Altre cose continuano a farmi pensare al COVID-19
Affatto
Un po’
Moderatamente
Molto
Estremamente
Sono consapevole di avere molti sentimenti riguardo al COVID-19, ma non me ne preoccupo
Affatto
Un po’
Moderatamente
Molto
Estremamente
Cerco di non pensare al COVID-19
Affatto
Un po’

Moderatamente
Molto
Estremamente
Ho difficoltà a concentrarmi
Affatto
Un po’
Moderatamente
Molto
Estremamente

Ti invitiamo a lasciare un commento su questo sondaggio:

Come hai saputo di noi?
Google
Annuncio su Facebook
Pagina Facebook sulla gravidanza
Sito web di Harvard
LinkedIn
Il post di un amico su un social
Passaparola
Altro

Korean Pregistry Survey

거주 중인 국가를 선택해주십시오.
(***list of all countries)

어느 도에 거주하고 계십니까?

어느 도시에 거주하고 계십니까?

당신이 임신?
예
아니

임신 몇 주차이십니까?
(***list 5 to 43)

출산한지 얼마나 됬습니까?
(***list not transferred)

당신은 몇 살입니까?
(***list 18 to 50)

자신의 인종으로 가장 적합한 것을 무엇입니까? (해당되는 모든 것을 체크하세요)
백인/코카시안
라틴/남미
아시아

남아시아
흑인
중동
하와이 원주민 또는 기타 태평양 섬 주민
아메리칸 인디언 또는 알래스카 원주민
기타/다인종

결혼 여부를 말씀해주십시오?
미혼
기혼
동거 중
별거 중
이혼
사별

(자신 포함) 가구 구성원은 몇 명입니까?
(***list not transferred)

교육 수준을 선택해주십시오?
미취학
초등학교
기타 고등학교
고등학교 졸업 또는 검정고시

기타 대학
대학 졸업장 또는 대학 학위
석사 학위
전문 학위
박사 학위

코로나 19 유행 발생 시
병원 및 의원의 의료 종사자였다
요양원에서 근무했다
정부 관계자였다
해당없음
잘 모르겠다

정부, 직장 또는 가족을 통해 의료 보험 혜택을 받고 있습니까?
(***list not transferred)

SARS-CoV- 2(코로나 19 의 원인이 되는 바이러스)에 대한 검사를 받았습니까? 검사를 받았다면 결과는 어떠했습니까?
양성반응으로 바이러스가 검출되었다.
음성반응으로 바이러스가 검출되지 않았다.
검사는 하였으나 결과가 나오기 전이거나 확실하지 않다
검사를 하지 않았다

건강 관리 전문가 (예 : 의사, 간호사)가 증상만을 근거로 COVID- 19 을 진단 한 적이 있습니까?
(***list not transferred)

COVID-19 에 감염되었거나 감염력이 있는 사람과 접촉한 적이 있습니까?
(***list not transferred)

COVID- 19 으로부터 자신과 다른 사람들을 안전하게 지키기 위해 지난 7 일 동안 다음 중 무엇을 했습니까? (복수 문항 체크)
마스크를 착용함
비누로 손을 씻거나 손 소독제로 하루에 여러 번 세척
내 주변을 소독함
손 소독제나 소독용 물티슈를 비축함
음식이나 물을 비축함
출장을 위한 항공편을 취소 또는 연기함
여가를 위한 항공편을 취소 또는 연기함
직장 또는 학교 활동 취소 또는 연기
개인 또는 사교 활동 취소 또는 연기
고위험군의 사람들과 접촉을 삼가함
공공 장소, 모임 또는 군중 피하기
외식을 자제함
재택근무를 함
의사를 방문
병원 외래 예약을 취소함
약을 비축함
기도함

2874 매일 코로나 19 에 관한 뉴스를 얼마나 자주 확인하십니까?
2875 전혀 안 함
2876 1 회 미만
2877 1 회
2878 2~4 회
2879 5~8 회
2880 9~16 회
2881 16 회 이상
2882 매일 소셜 미디어에서 코로나 19 에 관한 정보를 얼마나 자주 확인합니까? (카카오톡, 페이스북)
2883 전혀 안 함
2884 1 회 미만
2885 1 회
2886 2~4 회
2887 5~8 회
2888 9~16 회
2889 16 회 이상
2890 매일 메신저 및 소셜 미디어에서 코로나 19 에 관해 얼마나 자주 논의합니까? (예: WhatsApp, Twitter)
2891 전혀 안 함
2892 1 회 미만
2893 1 회
2894 2~4 회
2895 5~8 회

9~16 회
16 회 이상
매일 다른 사람과 코로나 19 에 관해 얼마나 자주 논의합니까?
전혀 안 함
1 회 미만
1 회
2~4 회
5~8 회
9~16 회
16 회 이상

COVID- 19 에 대해 얼마나 걱정하십니까?
(***list not transferred)

COVID-19 에 대해 가장 걱정하는 것이 무엇입니까? 해당되는 모든 것들을 고르세요:
내가 COVID-19 에 감염되어 가족들을 감염시킬 것 같다.
배우자가 COVID-19 에 감염되어 가족들을 감염시킬 것 같다.
가족 및 친구가 감염될 것 같다
COVID19 가 가계에 큰 영향을 줄 것 같다(예: 정리 해고)
출산 전 태아가 COVID19 에 감염될 것 같다
COVID19 가 출산 계획에 영향을 줄 것 같다
COVID19 로 인해 배우자나 출산 도우미가 출산 중 내 옆을 지켜주지 못할 것 같다
COVID19 확산 방지 조치로 인해 출산 후 가족들이 나와 아기를 보러 오지 못할 것이다.

내 다른 자녀들이 COVID19 에 감염될 것 같다
COVID19 확산 방지 조치로 인해 부모/ 조부모가 아기를 방문 할 수 없을 것 같다
COVID19 로 인해 모유 수유를 할 수 없을 것 같다
다른 자녀들에게 육아를 제대로 할 수 없을 것 같다
가족의 장례식에 참석할 수 없을 걸 같다
예약한 병원 방문을 못가거나 취소해야할 것 같다
친구나 친지들과 베이비 샤워를 할 수 없을 것 같다
기타

코로나 19 는 인생에서 어떤 부분에 부정적인 영향을 미쳤습니까?
수면
전혀 없음
조금
적당히
많이
다이어트
전혀 없음
조금
적당히
많이
피트니스
전혀 없음

2941 조금
2942 적당히
2943 많이
2944 작업
2945 전혀 없음
2946 조금
2947 적당히
2948 많이
2949 재정
2950 전혀 없음
2951 조금
2952 적당히
2953 많이
2954 가족
2955 전혀 없음
2956 조금
2957 적당히
2958 많이
2959 관계
2960 전혀 없음
2961 조금
2962 적당히

많이
종교적 준수
전혀 없음
조금
적당히
많이
인격 (예 : 인내, 신뢰)
전혀 없음
조금
적당히
많이

COVID- 19 대유행이 시작된 후 다음 중 어떤 일이 자주 발생 했습니까?
다른 사람들보다 예의 바른 대우를받지 못했습니다
거의 매일
적어도 일주일에 한번
한 달에 몇 번
전혀 없음
식당이나 상점에있는 다른 사람들보다 더 나쁜 서비스를 받았습니다
거의 매일
적어도 일주일에 한번
한 달에 몇 번

전혀 없음
사람들이 당신을 조심스럽게 대했다
거의 매일
적어도 일주일에 한번
한 달에 몇 번
전혀 없음
사람들로부터 모욕적인 말을 들었다
거의 매일
적어도 일주일에 한번
한 달에 몇 번
전혀 없음
당신은 위협 또는 괴롭힘
거의 매일
적어도 일주일에 한번
한 달에 몇 번
전혀 없음
당신은 신체적으로 폭행당했습니다
거의 매일
적어도 일주일에 한번
한 달에 몇 번
전혀 없음

이러한 경험의 주된 이유는 무엇이라고 생각하십니까? (해당 사항을 모두 선택하십시오)

조상 또는 국적

당신의 성별

인종 또는 민족

나이

당신의 종교

너의 키

당신의 체중

임신

성적 취향

귀하의 교육 또는 소득 수준

다른

지난 2 주를 기준으로 다음 질문에 답하십시오.

인생에서 중요한 것들을 통제 할 수 없다고 얼마나 자주 느꼈습니까?

전혀 없음

거의 없다

때때로

상당히 자주

매우 자주

개인 문제를 처리 할 수있는 능력에 대해 얼마나 자주 확신하십니까?

전혀 없음

거의 없다
때때로
상당히 자주
매우 자주
일이 잘 진행되고 있다고 얼마나 자주 느꼈습니까?
전혀 없음
거의 없다
때때로
상당히 자주
매우 자주
어려움이 너무 많아서 극복 할 수 없을 정도로 얼마나 자주 느꼈습니까?
전혀 없음
거의 없다
때때로
상당히 자주
매우 자주

지난 2 주 동안 다음 문항이 당신을 얼마나 귀찮게 했습니까?
긴장, 불안 또는 가장자리 느낌
전혀 없음
며칠
반일 이상

거의 매일
걱정을 멈추거나 통제 할 수 없음
전혀 없음
며칠
반일 이상
거의 매일
우울함, 우울함 또는 절망감
전혀 없음
며칠
반일 이상
거의 매일
일에 관심이 없거나 즐거움이 적다
전혀 없음
며칠
반일 이상
거의 매일

COVID-19 이후로, 아래와 같은 이유로 얼마나 자주 먹습니까?
우울하거나 울적해져서
거의 또는 전혀 없음
드물게
때때로

3073 자주
3074 거의 항상 또는 항상
3075 문제해결을 위한 힘을 얻기 위해
3076 거의 또는 전혀 없음
3077 드물게
3078 때때로
3079 자주
3080 거의 항상 또는 항상
3081 위안을 얻기 위해
3082 거의 또는 전혀 없음
3083 드물게
3084 때때로
3085 자주
3086 거의 항상 또는 항상
3087 스스로가 부적절하거나 쓸모 없다고 느껴져서
3088 거의 또는 전혀 없음
3089 드물게
3090 때때로
3091 자주
3092 거의 항상 또는 항상
3093 불쾌한 생각으로부터 벗어나기 위해
3094 거의 또는 전혀 없음

드물게
때때로
자주
거의 항상 또는 항상

다음 문장에 얼마나 동의하십니까?
인생에는 감사해야 할 것들이 많다
강하게 동의
동의하지 않는다
다소 동의하지 않음
동의하거나 동의하지 않음
일정 동의하다
동의하다
매우 동의
감사할 것들을 나열하다도면 꽤 길어질 것이다
강하게 동의
동의하지 않는다
다소 동의하지 않음
동의하거나 동의하지 않음
일정 동의하다
동의하다
매우 동의

감사해야 할 사람들이 많다
강하게 동의
동의하지 않는다
다소 동의하지 않음
동의하거나 동의하지 않음
일정 동의하다
동의하다
매우 동의

COVID-19 위기가 나에게 어떻게 영향을 미쳤는지 기술하시오.

COVID- 19 위기가 시작된 후 다음 사람들은 얼마나 지지해 주는가?
남편 또는 배우자
전혀
조금
적당히
상당히
매우
해당 없음
부모 또는 법적 보호자
전혀
조금
적당히

3140 상당히
3141 매우
3142 해당 없음
3143 어린이
3144 전혀
3145 조금
3146 적당히
3147 상당히
3148 매우
3149 해당 없음
3150 형제 자매
3151 전혀
3152 조금
3153 적당히
3154 상당히
3155 매우
3156 해당 없음
3157 친구
3158 전혀
3159 조금
3160 적당히
3161 상당히

매우
해당 없음
동료
전혀
조금
적당히
상당히
매우
해당 없음

COVID- 19 위기가 시작된 후, 다음 사람들은 얼마나 스트레스를 느낍니까?
남편 또는 배우자
전혀
조금
적당히
상당히
매우
해당 없음
부모 또는 법적 보호자
전혀
조금
적당히

3184 상당히
3185 매우
3186 해당 없음
3187 어린이
3188 전혀
3189 조금
3190 적당히
3191 상당히
3192 매우
3193 해당 없음
3194 형제 자매
3195 전혀
3196 조금
3197 적당히
3198 상당히
3199 매우
3200 해당 없음
3201 친구
3202 전혀
3203 조금
3204 적당히
3205 상당히

매우
해당 없음
동료
전혀
조금
적당히
상당히
매우
해당 없음

COVID-19 위기가 시작된 이후...
얼마나 자주 교제하지 못한다고 느끼십니까?
거의
일부 시간
자주
얼마나 자주 소외받고 있다고 느끼십니까?
거의
일부 시간
자주
얼마나 자주 고립되어 있다고 느끼십니까?
거의
일부 시간

자주

지난 7 일 동안의 경험에 대해 생각할 때, 다음 각 진술에 얼마나 강력하게 동의하는지 동의하십시오.
커뮤니티라고 부르는 것에 속해 있다고 생각하지 않습니다.
매우 동의
동의한다
어느정도 동의한다
동의하지도 부정하지도 않음
다소 동의하지 않음
동의하지 않는다
강하게 동의
나는 세상에 가치 있는 것을 내줄 수 있다
매우 동의
동의한다
어느정도 동의한다
동의하지도 부정하지도 않음
다소 동의하지 않음
동의하지 않는다
강하게 동의
나는 지역 사회의 다른 사람들과 가깝게 느낀다
매우 동의
동의한다

3250 어느정도 동의한다
3251 동의하지도 부정하지도 않음
3252 다소 동의하지 않음
3253 동의하지 않는다
3254 강하게 동의
3255 나는 세상에서 무슨 일이 일어나고 있는지 이해할 수 없다
3256 매우 동의
3257 동의한다
3258 어느정도 동의한다
3259 동의하지도 부정하지도 않음
3260 다소 동의하지 않음
3261 동의하지 않는다
3262 강하게 동의
3263 나의 공동체는 내 위로의 기반이다
3264 매우 동의
3265 동의한다
3266 어느정도 동의한다
3267 동의하지도 부정하지도 않음
3268 다소 동의하지 않음
3269 동의하지 않는다
3270 강하게 동의
3271 나는 사람들이 친절하다고 생각한다

매우 동의
동의한다
어느정도 동의한다
동의하지도 부정하지도 않음
다소 동의하지 않음
동의하지 않는다
강하게 동의

지난 7 일을 기준으로 다음 질문에 답하십시오.
조심스럽고 긴장된다
전혀
조금
적당히
상당히
매우
다른 일들로 인해 코로나 19 에 관해 계속 생각하게 된다
전혀
조금
적당히
상당히
매우
COVID19 에 대해 여러 감정이 있지만 제대로 대처하지 못하고 있다.

전혀
조금
적당히
상당히
매우
코로나 19 에 관해 생각하지 않으려고 노력 중이다
전혀
조금
적당히
상당히
매우
집중하는 데 어려움이 있다
전혀
조금
적당히
상당히
매우

이 설문 조사에 대한 의견을 보내주십시오.

우리에 대해 어떻게 알게 되셨습니까?
(***list not transferred)

Portuguese Pregistry survey

Em que país você vive?
(***list of countries)

Em que estado/província você mora?

Em qual cidade você vive?

Você está grávida?
Sim
Não

Com quantas semanas de gravidez você está?
(***list 5 to 43)

Quantos anos você tem?
(***list 18 to 50)

Com quais raças você mais se identifica? (marque todas as opções aplicáveis)
Branca/Caucasiana
Latina/Hispânica
Asiática
Sul da Ásia
Preto/Pardo
Oriente Médio
Nativo do Havaí ou de outras ilhas do Pacífico
Índio americano ou nativo do Alasca
Outro/Multiraça

Qual é seu estado civil?
Solteira
Casada

Vivendo com parceiro
Separada
Divorciada
Viúva

Quantas pessoas vivem em sua casa (incluindo você)?
1
2
3
4
5
6
7
8
9
10
Mais de 10

Qual o nível mais alto de educação que você alcançou?
Nunca frequentou a escola
Ensino Fundamental
Ensino médio incomplete
Ensino médio complete
Ensino superior incomplete
Ensino superior complete
Mestrado
Grau Profissional
Doutorado

Indique se durante a pandemia do COVID-19 você:
Era um profissional de saúde em um hospital ou clínica
Trabalhou em uma casa de repouso
Era um trabalhador essencial / chave (conforme definido pelo governo)

Nenhum desses
Não sabe

Você tem seguro médico através de seu governo, empregador ou membro da família?
(***list not transferred)

Você foi testado para SARS-CoV-2 (o vírus que causa o COVID-19)? Se sim, qual foi o resultado?
Positivo, eu tive o virus
Negativo, eu não tinha o virus
Sim, mas ainda não sei o resultado ou o resultado foi inconclusive
Não, eu não fui testado

Um profissional de saúde (por exemplo, médico, enfermeiro) diagnosticou você como tendo COVID- 19 apenas com base nos seus sintomas?
(***list not transferred)

Você já entrou em contato com uma pessoa que possui ou teve COVID-19?
(***list not transferred)

Qual das seguintes medidas você tomou nos últimos 7 dias para manter você e outras pessoas a salvo da COVID-19? (Selecione tudo que se aplica)
Usou uma máscara facial
Lavou as mãos com sabão, desinfetante para as mãos ou álcool em gel várias vezes por dia
Desinfetou superfícies ao seu redor
Estocou álcool em gel ou desinfetante para as mãos
Estocou alimentos ou água
Cancelou ou adiou viagens aéreas a trabalho
Cancelou ou adiou viagens aéreas de lazer
Cancelou ou adiou atividades de trabaho ou escolares
Cancelou ou adiou atividades pessoais ou sociais
Evitou contato com pessoas que podem ser de alto risco
Evitou espaços públicos, reuniões familiares ou de amigos ou multidões
Evitou comer em restaurantes

Trabalhou ou estudou em casa
Visitou um medico
Cancelou uma consulta médica
Estocou alguma medicação
Rezou

Com que frequência você verifica as notícias sobre COVID-19?

Nunca
<1 x / dia
1 x / dia
2-4 x / dia
5-8 x / dia
9-16 x / dia
> 16 x / dia

Com que frequência você checa as mídias sociais sobre a COVID-19? (por exemplo, WhatsApp, Facebook)
Nunca
<1 x / dia
1 x / dia
2-4 x / dia
5-8 x / dia
9-16 x / dia
> 16 x / dia

Com que frequência você discute a COVID-19 nas comunicações de massa? (por exemplo, grupo WhatsApp, Twitter)
Nunca
<1 x / dia
1 x / dia
2-4 x / dia
5-8 x / dia
9-16 x / dia
> 16 x / dia

Com que frequência você discute a COVID-19 com outra pessoa?
Nunca
<1 x / dia
1 x / dia
2-4 x / dia
5-8 x / dia
9-16 x / dia
> 16 x / dia

Você está preocupada com a COVID-19?
(***list not transferred)

E, relacionado à COVID-19, o que deixa você mais preocupada? Selecione tudo que se aplica:
Que eu contraia COVID-19 e traga a infecção para casa
Que meu(minha) parceiro(a) contraia COVID-19 e traga a infecção para casa
Que meus familiares / amigos sejam infectados com COVID-19
Que a pandemia de COVID-19 afete significativamente minha situação econômica / financeira (por exemplo, perder meu emprego)
Que meu bebê do qual estou grávida contraia COVID-19
Que a COVID-19 acarrete em alterações no meu plano de parto
Que meu(minha) parceiro(a) / acompanhante não possa ficar comigo durante o parto por causa da COVID-19
Que minha família não possa visitar a mim e ao bebê após o parto devido a medidas para evitar a disseminação da COVID-19
Que meu(s/minha/minhas) outro(s/a/as) filho(s/a/as) contraiam COVID-19
Que meus pais / avós não possam visitar o bebê por causa de medidas para prevenir a COVID-19
Que não possa amamentar por causa da COVID-19
Que eu não possa prestar cuidados adequados ao(s/à/às) meu(s/minha/minhas) outro(s/a/as) filho(s/a/as)
Que eu não possa comparecer ao funeral de um membro da família
Que minhas consultas médicas sejam canceladas ou eu não possa comparecer
Que eu não possa fazer um chá de bebê ou outra comemoração para o bebê com a família ou amigos
Outros

Até que ponto a COVID-19 impactou negativamente as seguintes áreas da sua vida?
Sono

3481 De modo nenhum
3482 Um pouco
3483 Moderadamente
3484 Muito
3485 Dieta/Alimentação
3486 De modo nenhum
3487 Um pouco
3488 Moderadamente
3489 Muito
3490 Atividades físicas
3491 De modo nenhum
3492 Um pouco
3493 Moderadamente
3494 Muito
3495 Trabalho
3496 De modo nenhum
3497 Um pouco
3498 Moderadamente
3499 Muito
3500 Finanças
3501 De modo nenhum
3502 Um pouco
3503 Moderadamente
3504 Muito
3505 Família
3506 De modo nenhum
3507 Um pouco
3508 Moderadamente
3509 Muito
3510 Relacionamentos
3511 De modo nenhum
3512 Um pouco
3513 Moderadamente

Muito
Práticas religiosas
De modo nenhum
Um pouco
Moderadamente
Muito
Temperamento (por exemplo paciência, confiança)
De modo nenhum
Um pouco
Moderadamente
Muito

Desde que a pandemia de COVID-19 começou, com que frequência alguma das seguintes coisas aconteceu com você?
Você foi tratada com menos cortesia do que as outras pessoas
Quase todos os dias
Pelo menos uma vez por semana
Algumas vezes por mês
Nunca
Você recebeu um serviço pior do que outras pessoas em restaurantes ou lojas
Quase todos os dias
Pelo menos uma vez por semana
Algumas vezes por mês
Nunca
As pessoas agiram como se tivessem medo de você
Quase todos os dias
Pelo menos uma vez por semana
Algumas vezes por mês
Nunca
Você foi xingada ou insultada
Quase todos os dias
Pelo menos uma vez por semana
Algumas vezes por mês
Nunca

Você foi ameaçada ou assediada
Quase todos os dias
Pelo menos uma vez por semana
Algumas vezes por mês
Nunca
Você foi agredida fisicamente
Quase todos os dias
Pelo menos uma vez por semana
Algumas vezes por mês
Nunca

Na sua opinião, qual é o principal motivo dessas experiências? (Escolha todas as opções aplicáveis)
Sua ascendência ou origem nacional
Seu gênero
Sua raça ou etnia
Sua idade
Sua religião
Tua altura
Seu peso
Sua gravidez
Sua orientação sexual
Sua escolaridade ou nível de renda
Outros

Responda às seguintes perguntas com base nas últimas 2 semanas.
Com que frequência você sentiu que era incapaz de controlar as coisas importantes da sua vida?
Nunca
Quase nunca
As vezes
Bastante Frequentemente
Muitas vezes
Com que frequência você se sentiu confiante em sua capacidade de lidar com seus problemas pessoais?
Nunca

Quase nunca
As vezes
Bastante Frequentemente
Muitas vezes
Com que frequência você sentiu que as coisas estavam indo como deveriam?
Nunca
Quase nunca
As vezes
Bastante Frequentemente
Muitas vezes
Com que frequência você sentiu que dificuldades estavam se acumulando tanto que você não conseguiria superá-las?
Nunca
Quase nunca
As vezes
Bastante Frequentemente
Muitas vezes

Com que frequência você se incomodou com os seguintes problemas nas últimas 2 semanas?
Sentir-se nervosa, ansiosa ou tensa
De modo nenhum
Muitos dias
Mais da metade dos dias
Quase todos os dias
Não conseguir de parar de se preocupar ou controlar as preocupações
De modo nenhum
Muitos dias
Mais da metade dos dias
Quase todos os dias
Sentir-se desanimada, deprimida ou sem esperança
De modo nenhum
Muitos dias
Mais da metade dos dias
Quase todos os dias

Sentir pouco interesse ou prazer em fazer as coisas
De modo nenhum
Muitos dias
Mais da metade dos dias
Quase todos os dias

Desde que a crise da COVID-19 começou, com que frequência você come ...
Porque você está deprimida ou triste
Quase nunca ou nunca
Raramente
As vezes
Frequentemente
Quase sempre ou sempre
Como uma maneira de ajudá-la a lidar com as dificuldades
Quase nunca ou nunca
Raramente
As vezes
Frequentemente
Quase sempre ou sempre
Como uma maneira de se confortar
Quase nunca ou nunca
Raramente
As vezes
Frequentemente
Quase sempre ou sempre
Porque você se sente inútil ou inadequada
Quase nunca ou nunca
Raramente
As vezes
Frequentemente
Quase sempre ou sempre
Como forma de evitar pensar em algo desagradável ou se distrair
Quase nunca ou nunca

Raramente
As vezes
Frequentemente
Quase sempre ou sempre

Indique o quanto você concorda com as seguintes declarações
Eu tenho muitas coisas/pessoas na vida pelas quais me sinto grata
Discordo fortemente
Discordar
Discordo parcialmente
Não concordo nem discord
De certo modo concord
Aceito
Concordo plenamente
Se eu tivesse que listar tudo pelo que me sinto grata, seria uma lista muito longa
Discordo fortemente
Discordar
Discordo parcialmente
Não concordo nem discord
De certo modo concord
Aceito
Concordo plenamente
Sou grata a uma grande variedade de pessoas
Discordo fortemente
Discordar
Discordo parcialmente
Não concordo nem discord
De certo modo concord
Aceito
Concordo plenamente

Por favor, descreva como a crise da COVID-19 mais afetou você:

Desde que a crise da COVID-19 começou, o quanto as seguintes pessoas te apoiam?
Marido/Companheiro(a)
De modo nenhum
Um pouco
Moderadamente
Bastante
Extremamente
N / A (não existe essa pessoa)
Pais ou responsáveis legais
De modo nenhum
Um pouco
Moderadamente
Bastante
Extremamente
N / A (não existe essa pessoa)
Filho(s/a/as)
De modo nenhum
Um pouco
Moderadamente
Bastante
Extremamente
N / A (não existe essa pessoa)
Irmão(ã/ãs/os)
De modo nenhum
Um pouco
Moderadamente
Bastante
Extremamente
N / A (não existe essa pessoa)
Amigos
De modo nenhum
Um pouco
Moderadamente

Bastante
Extremamente
N / A (não existe essa pessoa)
Colegas de trabalho
De modo nenhum
Um pouco
Moderadamente
Bastante
Extremamente
N / A (não existe essa pessoa)

Desde que a crise do COVID-19 começou, o quanto você se sente estressada pelas seguintes pessoas?
Marido/Companheiro(a)
De modo nenhum
Um pouco
Moderadamente
Bastante
Extremamente
N / A (não existe essa pessoa)
Pais ou responsáveis legais
De modo nenhum
Um pouco
Moderadamente
Bastante
Extremamente
N / A (não existe essa pessoa)
Filho(s/a/as)
De modo nenhum
Um pouco
Moderadamente
Bastante
Extremamente
N / A (não existe essa pessoa)

Irmão(ã/ãs/os)
De modo nenhum
Um pouco
Moderadamente
Bastante
Extremamente
N / A (não existe essa pessoa)
Amigos
De modo nenhum
Um pouco
Moderadamente
Bastante
Extremamente
N / A (não existe essa pessoa)
Colegas de trabalho
De modo nenhum
Um pouco
Moderadamente
Bastante
Extremamente
N / A (não existe essa pessoa)

Desde que a crise do COVID-19 começou...
Com que frequência você sente falta de companhia?
Quase nunca
Algumas vezes
Frequentemente
Com que frequência você se sente deixado de lado?
Quase nunca
Algumas vezes
Frequentemente
Com que frequência você se sente isolado dos outros?
Quase nunca

Algumas vezes
Frequentemente

Pensando em sua experiência NOS ÚLTIMOS 7 DIAS, indique o quanto você concorda ou discorda de cada uma das seguintes afirmações
Eu não sinto que pertenço a algo que eu chamaria de comunidade
Concordo plenamente
Aceita
De certo modo concord
Não concordo nem discord
Discordo parcialmente
Discordar
Discordo fortemente
Eu tenho algo valioso para dar ao mundo
Concordo plenamente
Aceita
De certo modo concord
Não concordo nem discord
Discordo parcialmente
Discordar
Discordo fortemente
Sinto-me próximo de outras pessoas na minha comunidade
Concordo plenamente
Aceita
De certo modo concord
Não concordo nem discord
Discordo parcialmente
Discordar
Discordo fortemente
Não consigo entender o que está acontecendo no mundo
Concordo plenamente
Aceita
De certo modo concord

Não concordo nem discord
Discordo parcialmente
Discordar
Discordo fortemente
Minha comunidade é uma fonte de conforto
Concordo plenamente
Aceita
De certo modo concord
Não concordo nem discord
Discordo parcialmente
Discordar
Discordo fortemente
Eu acredito que as pessoas são gentis
Concordo plenamente
Aceita
De certo modo concord
Não concordo nem discord
Discordo parcialmente
Discordar
Discordo fortemente

Responda às seguintes perguntas com base nos últimos 7 dias
Sinto-me vigilante ou em guarda
De modo nenhum
Um pouco
Moderadamente
Bastante
Extremamente
Outras coisas ficam me fazendo pensar sobre a COVID-19
De modo nenhum
Um pouco
Moderadamente
Bastante

Extremamente
Estou ciente de que tenho muitos sentimentos em relação ao COVID-19, mas não tenho lidado com eles
De modo nenhum
Um pouco
Moderadamente
Bastante
Extremamente
Eu tento não pensar na COVID-19
De modo nenhum
Um pouco
Moderadamente
Bastante
Extremamente
Tenho dificuldade em me concentrar
De modo nenhum
Um pouco
Moderadamente
Bastante
Extremamente

Envie comentários sobre esta pesquisa:

Como você ficou sabendo sobre nós?
(***list not transferred)

Russian Pregistry survey

В какой стране вы живете?
(***list of all countries)

В каком штате / провинции вы живете?

В каком городе вы живете?

Ты беременна?
Да
Нет

Сколько недель вы беременны?
(***list 5 to 43)

Как давно ты родила?
(***list not transferred)

Сколько вам лет?
(***list 18 to 50)

С какой расой вы больше всего себя идентифицируете? (отметьте все, которые относятся к вам)
Белая / Европейская
Латинская / Испанская
Азиатская
Южноазиатская
Негроидная
Ближневосточная
Коренная гавайка или представительница других коренных народов тихоокеанских островов
Индианка или уроженка Аляски
Другая / мультираса

Укажите ваше семейное положение?
Живу одна
Замужем
Живу с партнером
Проживаем раздельно
Разведена
Вдова

Сколько людей живет в вашем доме (включая вас)?
1
2
3
4
5
6
7
8
9
10
Более 10

Какое у вас образование?
Никогда не посещала школу
Начальная школа
Неоконченное среднее образование
Оконченное среднее образование или диплом общей квалификации (GED)
Неоконченное среднее профессиональное / высшее образование
Диплом колледжа или высшее образование
Степень магистра
Профессиональная степень
Докторская степень

Пожалуйста, укажите, если во время пандемии COVID-19 вы:

Являлись медицинским работником в больнице или клинике
Работали в доме престарелых
Являлись работником жизненно важных сфер общества (по определению правительства)
Ничего из вышеназванного
Не знаю

У вас есть государственная медицинская страховка, страховка от вашего работодателя или члена семьи?
Да
Нет

Проходили ли вы тестирование на SARS-CoV- 2 (вирус, вызывающий COVID- 19)? Если так, каков был результат?
Положительный, у меня был вирус
Отрицательный, у меня не было вируса
Да, но я пока не знаю результат или результат был неокончательным
Нет, я не проходила тест

Диагностировал ли у вас медицинский работник (например, врач, медсестра) COVID-19 только на основании ваших симптомов?
(***list not transferred)

Вы общались с людьми, зараженными или переболевшими COVID-19?
(***list not transferred)

Что вы сделали за последние 7 дней, чтобы защитить себя и других от COVID- 19? (Выберите все подходящие варианты)
Носила маску для лица
Мыла руки с мылом или дезинфицирующим средством для рук несколько раз в день
Дезинфицировала окружающие меня поверхности
Запаслась средствами для дезинфекции рук или дезинфицирующими салфетками
Запаслась продуктами питания или водой
Отменила или отложила авиаперелеты по работе
Отменила или отложила авиаперелеты на отдых
Отказалась или перенесла работу и учебу
Отменила или перенесла личные или социальные дела

Избегала контакта с людьми, которые находятся в зоне высокого риска
Избегала общественных мест, собраний или скоплений народа
Избегала мест общественного питания
Работала или училась дома
Посетила врача
Отменила визит к врачу
Запаслась лекарствами
Молилась

Как часто вы смотрите новости о COVID-19?
Никогда
<1 раза в день
1 раз в день
2-4 раза в день
5-8 раз в день
9-16 раз в день
> 16 раз в день
Как часто вы смотрите новости о COVID-19 в социальных сетях? (например, WhatsApp, Facebook)
Никогда
<1 раза в день
1 раз в день
2-4 раза в день
5-8 раз в день
9-16 раз в день
> 16 раз в день
Как часто вы обсуждаете COVID-19 в средствах массовой коммуникации? (например, группы WhatsApp, Twitter)
Никогда
<1 раза в день
1 раз в день
2-4 раза в день
5-8 раз в день
9-16 раз в день
> 16 раз в день

Как часто вы обсуждаете COVID-19 с другими людьми?
Никогда
<1 раза в день
1 раз в день
2-4 раза в день
5-8 раз в день
9-16 раз в день
> 16 раз в день

Насколько вы беспокоитесь о COVID-19?
(***list not transferred)

Что больше всего беспокоит вас в связи с пандемией COVID-19? Выберите все подходящие варианты:
Что я заражусь COVID-19 и принесу инфекцию домой
Что мой партнер заразится COVID-19 и принесет инфекцию домой
Что члены моей семьи / друзья заразятся COVID-19
Что пандемия COVID-19 существенно повлияет на мое экономическое положение / финансы (например, потеря моей работы)
Что мой еще нерожденный ребенок заразится COVID-19
Что COVID-19 повлияет на срок рождения моего ребенка
Что из-за COVID-19 мой партнер / близкий человек не сможет быть со мной во время родов
Что моя семья не сможет навестить меня и ребенка после родов из-за мер по предотвращению распространения COVID-19
Что мои другие дети заразятся COVID-19
Что мои родители / бабушка и дедушка не смогут посетить ребенка из-за мер по предотвращению распространения COVID-19
Что я не смогу кормить грудью из-за COVID-19
Что я не смогу обеспечить адекватный уход за другими своими детьми
Что я не смогу присутствовать на похоронах члена моей семьи
Отсутствие / отмена приема у врача
Что я не смогу показать ребенка или устроить другое детское торжество с семьей или друзьями
Другое

В какой степени COVID-19 отрицательно повлиял на следующие сферы вашей жизни?
Сон

4033 Нисколько
4034 Немного
4035 Умеренно
4036 Много
4037 Рацион питания
4038 Нисколько
4039 Немного
4040 Умеренно
4041 Много
4042 Фитнес
4043 Нисколько
4044 Немного
4045 Умеренно
4046 Много
4047 Работа
4048 Нисколько
4049 Немного
4050 Умеренно
4051 Много
4052 Финансы
4053 Нисколько
4054 Немного
4055 Умеренно
4056 Много
4057 Семья
4058 Нисколько
4059 Немного
4060 Умеренно
4061 Много
4062 Отношения
4063 Нисколько
4064 Немного
4065 Умеренно

Много
Соблюдение религии
Нисколько
Немного
Умеренно
Много
Характер (например, терпение, доверие)
Нисколько
Немного
Умеренно
Много

С момента начала пандемии COVID-19 как часто с вами случалось что-либо из следующего?
С вами обращались вежливее, чем с другими
Почти каждый день
По крайней мере раз в неделю
Несколько раз в месяц
Никогда
Вы получили худшее обслуживание, чем другие люди в ресторанах или магазинах
Почти каждый день
По крайней мере раз в неделю
Несколько раз в месяц
Никогда
Почти каждый день
По крайней мере раз в неделю
Несколько раз в месяц
Никогда
Люди вели себя так, как будто они вас боятся
Почти каждый день
По крайней мере раз в неделю
Несколько раз в месяц
Никогда
Вас обзывали или оскорбляли

Почти каждый день
По крайней мере раз в неделю
Несколько раз в месяц
Никогда
Вам угрожали или преследовали
Почти каждый день
По крайней мере раз в неделю
Несколько раз в месяц
Никогда
Вы подвергались физическому насилию
Почти каждый день
По крайней мере раз в неделю
Несколько раз в месяц
Никогда

Как вы думаете, что является основной причиной этих переживаний? (Выберите все подходящие варианты)
Ваше происхождение или национальное происхождение
Ваш пол
Ваша раса или этническая принадлежность
Ваш возраст
Ваша религия
Ваш рост
Ваш вес
Ваша беременность
Ваша сексуальная ориентация
Ваше образование или уровень дохода
Другое

Пожалуйста, ответьте на следующие вопросы на основании последних 2 недель.
Как часто вы чувствовали, что не можете контролировать важные вещи в своей жизни?
Никогда
Почти никогда
Иногда

Довольно часто
Очень часто
Как часто вы были уверены в своей способности справляться со своими личными проблемами?
Никогда
Почти никогда
Иногда
Довольно часто
Очень часто
Как часто вы чувствовали, что все идет так, как вы планировали?
Никогда
Почти никогда
Иногда
Довольно часто
Очень часто
Как часто вы ощущали, что у вас накопилось так много трудностей, что вы не можете их преодолеть?
Никогда
Почти никогда
Иногда
Довольно часто
Очень часто

Как часто за последние две недели вас беспокоили следующие проблемы?
Вы чувствовали себя нервной, тревожной или раздраженной
Ни разу
Несколько дней
Более половины дней
Почти каждый день
Вы не могли остановить или контролировать беспокойство
Ни разу
Несколько дней
Более половины дней
Почти каждый день
Вас охватывало чувство подавленности, депрессии или безнадежности

Ни разу
Несколько дней
Более половины дней
Почти каждый день
Вы испытывали мало интереса или удовольствия от занятий
Ни разу
Несколько дней
Более половины дней
Почти каждый день

С начала кризиса COVID-19 как часто вы едите ...
Потому что вы в депрессии или грусти
Почти никогда или никогда
Редко
Иногда
Часто
Почти всегда или всегда
Чтобы помочь себе справиться с ситуацией
Почти никогда или никогда
Редко
Иногда
Часто
Почти всегда или всегда
Чтобы утешить себя
Почти никогда или никогда
Редко
Иногда
Часто
Почти всегда или всегда
Потому что вы чувствуете себя бесполезной или ненужной
Почти никогда или никогда
Редко
Иногда

Часто
Почти всегда или всегда
Чтобы не думать о чем-либо неприятном или отвлечься
Почти никогда или никогда
Редко
Иногда
Часто
Почти всегда или всегда

Укажите, насколько вы согласны со следующими утверждениями
В моей жизни много вещей, за которые я благодарна
Категорически не согласна
Не согласна
Не совсем согласна
Ни согласна, ни не согласна
Отчасти согласна
Согласна
Полностью согласна
Если бы мне пришлось перечислить все, за что я благодарна, это был бы очень длинный список
Категорически не согласна
Не согласна
Не совсем согласна
Ни согласна, ни не согласна
Отчасти согласна
Согласна
Полностью согласна
Я благодарна широкому кругу людей
Категорически не согласна
Не согласна
Не совсем согласна
Ни согласна, ни не согласна
Отчасти согласна
Согласна

Полностью согласна

Пожалуйста, опишите, каким образом кризис COVID-19 повлиял на вас больше всего:

С тех пор, как начался кризис COVID-19, насколько сильно вас поддерживают перечисленные люди?
Муж или другой близкий человек
Нисколько
Немного
Средне
Выше среднего
Сильно
Такого человека нет
Родители или законные опекуны
Нисколько
Немного
Средне
Выше среднего
Сильно
Такого человека нет
Дети
Нисколько
Немного
Средне
Выше среднего
Сильно
Такого человека нет
Братья и сестры
Нисколько
Немного
Средне
Выше среднего
Сильно
Такого человека нет

Друзья
Нисколько
Немного
Средне
Выше среднего
Сильно
Такого человека нет
Коллеги
Нисколько
Немного
Средне
Выше среднего
Сильно
Такого человека нет

С момента начала кризиса COVID-19 как сильно, по вашим ощущениям, переживают перечисленные люди?
Муж или другой близкий человек
Нисколько
Немного
Средне
Выше среднего
Сильно
Такого человека нет
Родители или законные опекуны
Нисколько
Немного
Средне
Выше среднего
Сильно
Такого человека нет
Дети
Нисколько
Немного

Средне
Выше среднего
Сильно
Такого человека нет
Братья и сестры
Нисколько
Немного
Средне
Выше среднего
Сильно
Такого человека нет
Друзья
Нисколько
Немного
Средне
Выше среднего
Сильно
Такого человека нет
Коллеги
Нисколько
Немного
Средне
Выше среднего
Сильно
Такого человека нет

С начала кризиса COVID-19…
Как часто вы чувствуете, что вам не хватает общения?
Почти никогда
Иногда
Часто
Как часто вы чувствуете себя обделенной?
Почти никогда

Иногда
Часто
Как часто вы чувствуете себя изолированной от других?
Почти никогда
Иногда
Часто

Подумайте о своем опыте за последние 7 дней и укажите, насколько сильно вы согласны или не согласны с каждым из следующих утверждений
Я не чувствую, что принадлежу к чему-либо, что можно назвать сообществом
Полностью согласна
Согласна
Отчасти согласна
Ни согласна, ни не согласна
Не совсем согласна
Не согласна
Категорически не согласна
У меня есть нечто ценное, что я могу дать миру
Полностью согласна
Согласна
Отчасти согласна
Ни согласна, ни не согласна
Не совсем согласна
Не согласна
Категорически не согласна
Я чувствую близость с другими людьми в моем сообществе
Полностью согласна
Согласна
Отчасти согласна
Ни согласна, ни не согласна
Не совсем согласна
Не согласна
Категорически не согласна

Я не могу понять, что происходит в мире
Полностью согласна
Согласна
Отчасти согласна
Ни согласна, ни не согласна
Не совсем согласна
Не согласна
Категорически не согласна
Мое сообщество является источником комфорта
Полностью согласна
Согласна
Отчасти согласна
Ни согласна, ни не согласна
Не совсем согласна
Не согласна
Категорически не согласна
Я считаю, что люди добрые
Полностью согласна
Согласна
Отчасти согласна
Ни согласна, ни не согласна
Не совсем согласна
Не согласна
Категорически не согласна

Пожалуйста, ответьте на следующие вопросы на основании последних 7 дней
Я чувствую себя бдительной или настороженной
Нисколько
Немного
Средне
Выше среднего
Сильно
Многие вещи заставляют меня думать о COVID-19

Нисколько
Немного
Средне
Выше среднего
Сильно
Я знаю, что у меня много чувств к COVID-19, но я не разбираюсь в них
Нисколько
Немного
Средне
Выше среднего
Сильно
Я стараюсь не думать о COVID-19
Нисколько
Немного
Средне
Выше среднего
Сильно
У меня проблемы с концентрацией
Нисколько
Немного
Средне
Выше среднего
Сильно

Пожалуйста, оставьте отзыв об этом опросе:
Как Вы узнали о нас?
(***list not transferred)

Turkish Pregistry survey

Hangi ülkede yaşıyorsunuz?
(***list of all countries)

Hangi eyalet / ilde yaşıyorsunuz?

Hangi şehirde yaşıyorsunuz?

Hamile misin?
Evet
YOK HAYIR

Kaç hafta hamilesiniz?
(***list 5 to 43)

Ne kadar zaman önce doğum yaptınız?
(***list not transferred)

Kaç yaşındasınız?
(***list 18 to 50)

Kendinizi aşağıdakilerden hangisi olarak tanımlarsınız? (Size uygun olanların tamamını seçiniz)
Beyaz / Kafkas
İspanyol / Latin
Asya
Güney Asyalı
Siyah
Orta Doğu
Yerli Hawaii veya Diğer Pasifik Adalı
Amerikan Kızılderili veya Alaska Yerli
Diğer / Çok ırklı

Medeni durumunuz nedir?
Tek
Evli
Eş ile yaşamak
Ayrılmış
Boşanmış
Dul

Evinizde kaç kişi yaşıyor (siz de dahil)?
1
2
3
4
5
6
7
8
9
10
10 tan daha fazla

Aldığınız en yüksek eğitim seviyesi nedir?
Hiç okula gitmedim
İlkokul
Bazı liseler
Lise mezunu veya genel denklik diploması (GED)
Bazı kolejler / üniversiteler
Üniversite diploması veya üniversite derecesi
Yüksek lisans
Profesyonel derece
Doktora derecesi

Lütfen COVID- 19 sırasında salgını yapıp yapmadığınızı belirtin:

Bir hastane veya klinikte sağlık çalışanı mıydı?
Huzurevinde çalıştı
Önemli / kilit bir çalışan mıydı (hükümet tarafından tanımlandığı gibi)
Bunlardan hiçbiri
Bilmiyorum

Devletiniz, işvereniniz veya bir aile üyesi aracılığıyla sağlık sigortanız var mı?
Evet
YOK HAYIR

SARS-CoV- 2 (COVID- 19 'a neden olan virüs) için test edildi mi? Eğer öyleyse, sonuç ne oldu?
Olumlu, virüsü aldım
Olumsuz, bende virüs yoktu
Evet, ancak sonucu henüz bilmiyorum veya sonuç sonuçsuz kaldı
Hayır, test edilmedim

Bir sağlık uzmanı (örn. Doktor, hemşire) belirtilerinize dayanarak COVID- 19 olduğunu teşhis etti mi?
(***list not transferred)

COVID- 19 olan veya olan bir kişiyle görüştünüz mü?
Evet
YOK HAYIR
Olabilir

Kendinizi ve başkalarını COVID- 19 'den korumak için son 7 gün içinde aşağıdakilerden hangisini yaptınız? (Geçerli olanların tümünü seçin)
Yüz maskesi taktım
Ellerinizi sabunla veya kullanılmış el dezenfektanıyla günde birkaç kez yıkadım
Etrafımdaki yüzeyleri dezenfektanla temizledim
Stoklanmış el dezenfektanı veya dezenfektan mendil
Stoklanmış yiyecek veya su
İş için iptal edilmiş veya ertelenmiş hava seyahati
Zevk için iptal edilmiş veya ertelenmiş hava yolculuğu

İptal edilen veya ertelenen iş veya okul etkinlikleri
İptal edilen veya ertelenen kişisel veya sosyal etkinlikler
Yüksek riskli kişilerle temastan kaçındım
Kamusal alanlardan, toplantılardan veya kalabalıklardan kaçındım
Restoranlarda yemek yemekten kaçının
Evde çalıştı ya da okudu
Bir doktora gittim
Doktor randevusu iptal edildi
Stoklanmış ilaç
Dua

COVID- 19 ile ilgili haberleri ne sıklıkla kontrol ediyorsunuz?
Asla
<1 x / gün
1 x / gün
2-4 x / gün
5-8 x / gün
9-16 x / gün
> 16 x / gün
COVID- 19 ile ilgili sosyal medyayı ne sıklıkla kontrol ediyorsunuz? (örneğin WhatsApp, Facebook)
Asla
<1 x / gün
1 x / gün
2-4 x / gün
5-8 x / gün
9-16 x / gün
> 16 x / gün
Toplu iletişimde COVID- 19'u sıklıkla tartışıyorsunuz? (örneğin WhatsApp grubu, Twitter)
Asla
<1 x / gün
1 x / gün
2-4 x / gün
5-8 x / gün

9-16 x / gün
> 16 x / gün
COVID- 19 \'i başka bir kişiyle ne sıklıkta tartışıyorsunuz?
Asla
<1 x / gün
1 x / gün
2-4 x / gün
5-8 x / gün
9-16 x / gün
> 16 x / gün

COVID- 19 konusunda ne kadar endişelisiniz?
(***list not transferred)

COVID- 19 sizi en çok endişelendiren ne olacak? Geçerli olanların tümünü seçin:
COVID- 19 alacağım ve enfeksiyonu eve getireceğim
Eşimin COVID- 19 alacağını ve enfeksiyonu eve getireceğini
Aile üyelerimin / arkadaşlarımın COVID- 19 ile enfekte olacağını
COVID- 19 salgınının ekonomik durumumu / finansmanımı önemli ölçüde etkileyeceği (örneğin, işimi kaybedeceğim)
Doğmamış bebeğimin COVID alacağı- 19
Bu COVID- 19, teslimat planımdaki değişiklikler anlamına gelecek
Eşim / destek görevlimin COVID- 19 nedeniyle teslimat sırasında benimle olamayacağı
COVID- 19 yayılmasını önleyici tedbirler nedeniyle ailemin doğumdan sonra beni ve bebeği ziyaret edemeyeceği
Diğer çocuklarım COVID- 19 alacak
Annem ve büyükannemin ve büyükbabamın COVID\'yi durdurma önlemleri nedeniyle bebeği ziyaret edemeyeceği
COVID- 19 nedeniyle emziremeyeceğim
Diğer çocuklarım için yeterli çocuk bakımı sağlayamayacağımı
Bir aile üyesinin cenaze törenine katılamayacağım
Doktor randevularını kaçırmak / iptal etmek
Aileniz veya arkadaşlarınızla bebek duşu veya başka bir bebek kutlaması yapamayacağımı
Diğer

COVID- 19 yaşamınızın aşağıdaki alanlarını ne ölçüde olumsuz etkiledi?

4588 Uyku
4589 Bir şey değil
4590 Birazcık
4591 Kısmen
4592 Çok
4593 Diyet
4594 Bir şey değil
4595 Birazcık
4596 Kısmen
4597 Çok
4598 Fitness
4599 Bir şey değil
4600 Birazcık
4601 Kısmen
4602 Çok
4603 İş
4604 Bir şey değil
4605 Birazcık
4606 Kısmen
4607 Çok
4608 Finans
4609 Bir şey değil
4610 Birazcık
4611 Kısmen
4612 Çok
4613 Aile
4614 Bir şey değil
4615 Birazcık
4616 Kısmen
4617 Çok
4618 İlişkiler
4619 Bir şey değil
4620 Birazcık

Kısmen
Çok
Dini gözlem
Bir şey değil
Birazcık
Kısmen
Çok
Karakter (örn. Sabır, güven)
Bir şey değil
Birazcık
Kısmen
Çok

COVID- 19 salgını başladığından beri, aşağıdakilerden ne kadar sıklıkta oldu?
Diğer insanlardan daha az nezaketle tedavi gördünüz
Neredeyse her gün
En az haftada bir kez
Ayda birkaç kez
Asla
Restoranlarda veya mağazalarda diğer insanlardan daha kötü hizmet aldınız
Neredeyse her gün
En az haftada bir kez
Ayda birkaç kez
Asla
İnsanlar senden korkuyor gibi davrandılar
Neredeyse her gün
En az haftada bir kez
Ayda birkaç kez
Asla
Size isimler denildi veya hakaret edildi
Neredeyse her gün
En az haftada bir kez
Ayda birkaç kez

Asla
Tehdit edildin veya taciz edildin
Neredeyse her gün
En az haftada bir kez
Ayda birkaç kez
Asla
Fiziksel olarak saldırıya uğradın
Neredeyse her gün
En az haftada bir kez
Ayda birkaç kez
Asla

Sizce bu deneyimlerin ana nedeni nedir? (Geçerli olanların tümünü seçin)
Atalarınız veya ulusal kökenleriniz
Senin cinsiyet
Irkınız veya etnik kökeniniz
Yaşınız
Dininiz
Senin boyun
Senin kilon
Hamileliğiniz
Cinsel yöneliminiz
Eğitim veya gelir seviyeniz
Diğer

Lütfen aşağıdaki soruları son 2 haftaya göre cevaplayın.
Yaşamınızdaki önemli şeyleri ne sıklıkta kontrol edemediğinizi ne sıklıkla hissettiniz?
Asla
Neredeyse hiç
Ara sıra
Oldukça sık
Çok sık
Kişisel sorunlarınızla başa çıkabilme yeteneğiniz konusunda ne sıklıkla kendinize güveniyorsunuz?

Asla
Neredeyse hiç
Ara sıra
Oldukça sık
Çok sık
İşlerin yolunda gittiğini ne sıklıkla hissettin?
Asla
Neredeyse hiç
Ara sıra
Oldukça sık
Çok sık
Ne sıklıkla zorlukların üstüste geldiği ve artık başa çıkamayacağınızı düşünüyorsunuz?
Asla
Neredeyse hiç
Ara sıra
Oldukça sık
Çok sık

Son 2 hafta boyunca aşağıdaki sorunlardan ne sıklıkta rahatsız oldunuz?
Sinir, endişeli veya sınırda hissetmek
Bir şey değil
Birkaç gün
Yarım günden fazla
Neredeyse her gün
Endişelenmeyi durduramamak veya kontrol edememek
Bir şey değil
Birkaç gün
Yarım günden fazla
Neredeyse her gün
Aşağı, depresif veya umutsuz hissetmek
Bir şey değil
Birkaç gün
Yarım günden fazla

Neredeyse her gün
Bir şeyler yapmak için çok az ilgi veya zevk
Bir şey değil
Birkaç gün
Yarım günden fazla
Neredeyse her gün

COVID- 19 krizi başladığından beri ne sıklıkta yiyorsunuz ...
Çünkü depresyonda ya da üzgün
Neredeyse asla ya da asla
Nadiren
Ara sıra
Sık sık
Neredeyse her zaman veya her zaman
Başa çıkmana yardımcı olmanın bir yolu olarak
Neredeyse asla ya da asla
Nadiren
Ara sıra
Sık sık
Neredeyse her zaman veya her zaman
Kendinizi rahatlatmanın bir yolu olarak
Neredeyse asla ya da asla
Nadiren
Ara sıra
Sık sık
Neredeyse her zaman veya her zaman
Çünkü kendinizi değersiz veya yetersiz hissediyorsunuz
Neredeyse asla ya da asla
Nadiren
Ara sıra
Sık sık
Neredeyse her zaman veya her zaman
Hoş olmayan bir şey hakkında düşünmekten kaçınmanın veya kendinizi rahatsız etmenin bir yolu olarak

Neredeyse asla ya da asla
Nadiren
Ara sıra
Sık sık
Neredeyse her zaman veya her zaman

Aşağıdaki ifadelere ne kadar katıldığınızı belirtin
Hayatta minnettar olmak için çok şeyim var
Kesinlikle katılmıyorum
Katılmıyorum
Biraz katılmıyorum
Ne katılıyorum ne katılmıyorum
Biraz katılıyorum
Katılıyorum
Kesinlikle katılıyorum
Minnettar olduğumu hissettiğim her şeyi listelesem çok uzun bir liste olurdu
Kesinlikle katılmıyorum
Katılmıyorum
Biraz katılmıyorum
Ne katılıyorum ne katılmıyorum
Biraz katılıyorum
Katılıyorum
Kesinlikle katılıyorum
Çok çeşitli insanlara minnettarım
Kesinlikle katılmıyorum
Katılmıyorum
Biraz katılmıyorum
Ne katılıyorum ne katılmıyorum
Biraz katılıyorum
Katılıyorum
Kesinlikle katılıyorum

Lütfen COVID- 19 krizinin sizi en çok nasıl etkilediğini açıklayın:

COVID- 19 krizi başladığından beri, aşağıdaki insanlar ne kadar destekleyici?
Eşler veya diğer önemli kişiler
Hiç değil
Birazcık
Kısmen
Biraz
Son derece
Yok (böyle bir kişi yok)
Ebeveynler veya yasal vasiler
Hiç değil
Birazcık
Kısmen
Biraz
Son derece
Yok (böyle bir kişi yok)
Çocuklar
Hiç değil
Birazcık
Kısmen
Biraz
Son derece
Yok (böyle bir kişi yok)
Kardeşler
Hiç değil
Birazcık
Kısmen
Biraz
Son derece
Yok (böyle bir kişi yok)
Arkadaş
Hiç değil
Birazcık

Kısmen
Biraz
Son derece
Yok (böyle bir kişi yok)
İş arkadaşları
Hiç değil
Birazcık
Kısmen
Biraz
Son derece
Yok (böyle bir kişi yok)

COVID- 19 krizi başladığından beri, aşağıdaki kişiler tarafından ne kadar stresli hissediyorsunuz?
Eşler veya diğer önemli kişiler
Hiç değil
Birazcık
Kısmen
Biraz
Son derece
Yok (böyle bir kişi yok)
Ebeveynler veya yasal vasiler
Hiç değil
Birazcık
Kısmen
Biraz
Son derece
Yok (böyle bir kişi yok)
Çocuklar
Hiç değil
Birazcık
Kısmen
Biraz
Son derece

Yok (böyle bir kişi yok)
Kardeşler
Hiç değil
Birazcık
Kısmen
Biraz
Son derece
Yok (böyle bir kişi yok)
Arkadaş
Hiç değil
Birazcık
Kısmen
Biraz
Son derece
Yok (böyle bir kişi yok)
İş arkadaşları
Hiç değil
Birazcık
Kısmen
Biraz
Son derece
Yok (böyle bir kişi yok)

COVID- 19 krizi başladığından beri-
Ne sıklıkla arkadaşlığınız olmadığını düşünüyorsunuz?
Neredeyse hiç
Bazen
Sık sık
Ne kadar sıklıkla dışlanmış hissediyorsunuz?
Neredeyse hiç
Bazen
Sık sık
Ne sıklıkla başkalarından izole olmuş hissediyorsunuz?

Neredeyse hiç
Bazen
Sık sık

GEÇMİŞ 7 GÜNDEKİ deneyiminizi düşünerek, lütfen aşağıdaki ifadelerin her birine ne kadar katılıp katılmadığınızı belirtiniz
Topluluk dediğim herhangi bir şeye ait olduğumu hissetmiyorum
Kesinlikle katılıyorum
Katılıyorum
Biraz katılıyorum
Ne katılıyorum ne katılmıyorum
Biraz katılmıyorum
Katılmıyorum
Kesinlikle katılmıyorum
Dünyaya verecek değerli bir şeyim var
Kesinlikle katılıyorum
Katılıyorum
Biraz katılıyorum
Ne katılıyorum ne katılmıyorum
Biraz katılmıyorum
Katılmıyorum
Kesinlikle katılmıyorum
Topluluğumdaki diğer insanlara yakın hissediyorum
Kesinlikle katılıyorum
Katılıyorum
Biraz katılıyorum
Ne katılıyorum ne katılmıyorum
Biraz katılmıyorum
Katılmıyorum
Kesinlikle katılmıyorum
Dünyada neler olup bittiğini anlamıyorum
Kesinlikle katılıyorum
Katılıyorum
Biraz katılıyorum

Ne katılıyorum ne katılmıyorum
Biraz katılmıyorum
Katılmıyorum
Kesinlikle katılmıyorum
Topluluğum bir rahatlık kaynağı
Kesinlikle katılıyorum
Katılıyorum
Biraz katılıyorum
Ne katılıyorum ne katılmıyorum
Biraz katılmıyorum
Katılmıyorum
Kesinlikle katılmıyorum
İnsanların kibar olduğuna inanıyorum
Kesinlikle katılıyorum
Katılıyorum
Biraz katılıyorum
Ne katılıyorum ne katılmıyorum
Biraz katılmıyorum
Katılmıyorum
Kesinlikle katılmıyorum

Lütfen aşağıdaki soruları son 7 güne göre cevaplayın
Dikkatli veya tetikte hissediyorum
Hiç değil
Birazcık
Kısmen
Biraz
Son derece
Diğer şeyler COVID- 19 hakkında beni düşündürüyor
Hiç değil
Birazcık
Kısmen
Biraz

Son derece
COVID- 19 hakkında çok fazla hislerimin olduğunun farkındayım, ancak onlarla ilgilenmiyorum
Hiç değil
Birazcık
Kısmen
Biraz
Son derece
COVID- 19 hakkında düşünmemeye çalışıyorum
Hiç değil
Birazcık
Kısmen
Biraz
Son derece
Konsantre olmakta zorlanıyorum
Hiç değil
Birazcık
Kısmen
Biraz
Son derece

Lütfen bu anketle ilgili görüşlerinizi bildiriniz:

Bizden nasıl haberiniz oldu?
(***list not transferred)

Zhongwen Pregistry survey

您住在哪个国家？
(***list of all countries)

您居住在哪个州/省？

你住在哪个城市？

你怀孕了吗？
是
没有

您怀孕几周了？
(***list 5 to 43)

您多久以前生育的？
(***list not transferred)

您多少岁？
(***list 18 to 50)

您属于哪个种族？(选择所有符合项)
白/高加索人
拉丁/西班牙文
亚洲人
南亚
黑色
中东人

夏威夷原住民或其他太平洋岛民
美洲印第安人或阿拉斯加原住民
其他/多种族

您的婚姻状况如何？
单身
已婚
与伴侣同住
分居
离异
丧偶

您家中有几口人（包括您自己）？
(***list not transferred)

您获得的最高学历是什么？
从未上过学
小学
高中肄业
高中毕业或同等学历
大学肄业
大学文凭或大学学位
硕士
专业学位
博士学位

请指出在新冠肺炎大流行期间您是否：

是医院或诊所的医护人员

在疗养院工作

是防疫中的必需人员（由政府定义）

都不是

不知道

您是否通过政府，所在企业或家庭成员获得医疗保险？

(***list not transferred)

您是否做了 SARS-CoV- 2（导致新冠肺炎的病毒）测试？如果是这样，结果是什么**？**

结果阳性，我感染了病毒

结果阴性，我没有感染

是的，但我尚不知道结果或结果尚不确定

否，**我没有做**过测试

医护人员（例如医生，护士）是否已根据您的症状将您诊断为新冠肺炎**？**

(***list not transferred)

您是否曾与感染新冠肺炎的人接触?

(***list not transferred)

在最近 7 **天内**，**您**为保护自己和他人免受新冠肺炎的侵害，执行了以下哪些操作？（选择所有符合条件的）

戴口罩

每天用肥皂或洗衣液洗手几次

对周围表面进行消毒

储备洗手液或消毒湿巾

储备食物或水

取消或推迟商务航空旅行

取消或推迟航空旅行

取消或推迟工作或学校活动

取消或推迟个人或社交活动

避免与高风险人士接触

避开公共场所，聚会或人群

避免在餐厅吃饭

在家工作或学习

去看医生

取消了医生的预约

储备药物

祈祷

您多久查看一次有关新冠肺炎的新闻？

从不

<1 x /天

1 x /天

2-4 x /天

5-8 x /天

9-16 x /天

> 16 x /天

您多久检查一次有关新冠肺炎的社交媒体？（例如，WhatsApp，Facebook）

从不

<1 x /天
1 x /天
2-4 x /天
5-8 x /天
9-16 x /天
> 16 x /天
您在大众通讯工具中多久讨论一次新冠肺炎？（**例如**，WhatsApp 组，Twitter）
从不
<1 x /天
1 x /天
2-4 x /天
5-8 x /天
9-16 x /天
> 16 x /天
您多久与他人讨论一次新冠肺炎？
从不
<1 x /天
1 x /天
2-4 x /天
5-8 x /天
9-16 x /天
> 16 x /天

您对新冠肺炎有多担心？
(***list not transferred)

那么，**新冠肺炎哪些方面**让您最担心？选择所有符合条件的：
我将感染新冠肺炎并将病毒带回家

我的伴侣将感染新冠肺炎并将病毒带回家
我的家人/朋友将被感染新冠肺炎
新冠肺炎大流行将严重影响我的经济状况/财务状况（例如，失业）
我未出生的婴儿将感染新冠肺炎
新冠肺炎将意味着更改我的分娩计划
由于新冠肺炎, 我的伴侣和亲人将无法在分娩时和我呆在一起
由于采取了防止新冠肺炎传播的措施，我的家人在分娩后将无法拜访我和婴儿
我的其他孩子将感染新冠肺炎
我的父母/祖父母由于采取了预防新冠肺炎的措施而无法看望婴儿
由于新冠肺炎，我将无法进行母乳喂养
我将无法为其他孩子提供足够的托儿服务
我将无法参加家庭成员的葬礼
减少/取消与医生的预约
我将无法与家人或朋友进行婴儿送礼会或其他婴儿庆祝活动
其他

新冠肺炎在多大程度上对您的以下生活产生了负面影响？
睡觉
一点也不
一点点
适度
很多
饮食
一点也不
一点点

5134	适度
5135	很多
5136	健身
5137	一点也不
5138	一点点
5139	适度
5140	很多
5141	工作
5142	一点也不
5143	一点点
5144	适度
5145	很多
5146	财务
5147	一点也不
5148	一点点
5149	适度
5150	很多
5151	家庭
5152	一点也不
5153	一点点
5154	适度
5155	很多
5156	人际关系
5157	一点也不
5158	一点点
5159	适度
5160	很多
5161	宗教活动
5162	一点也不

一点点
适度
很多
性格（例如耐心，信任）
一点也不
一点点
适度
很多

自从新冠肺炎大流行开始以来，以下任何事情发生在您身上的频率是多少？
您受到的礼遇比其他人少
几乎每天
至少每周一次
一个月几次
从不
您在餐馆或商店获得的服务比其他人差
几乎每天
至少每周一次
一个月几次
从不
人们的举止好像在怕您
几乎每天
至少每周一次
一个月几次
从不
您受到了谩骂或侮辱
几乎每天
至少每周一次

一个月几次
从不
您受到威胁或骚扰
几乎每天
至少每周一次
一个月几次
从不
您被人殴打了
几乎每天
至少每周一次
一个月几次
从不

您认为这些经历的主要原因是什么？（选择所有适用项）
您的血统或国籍
您的性别
您的种族或民族
您的年龄
您的信仰
您的身高
您的体重
您怀孕了
您的性取向
您的学历或收入水平
其他

请根据最近 2 周来回答以下问题。

您多久觉得自己无法控制生活中的重要事情？
从不
几乎从不
有时
相当频繁
常常
您对自己处理个人问题的能力有多少自信？
从不
几乎从不
有时
相当频繁
常常
您多久觉得事情进展顺利？
从不
几乎从不
有时
相当频繁
常常
您多久感到困难堆积如山，以致无法克服？
从不
几乎从不
有时
相当频繁
常常

在最近 2 周内，您多久被以下问题困扰？

感到紧张，焦虑或不安

一点也不

几天

超过半天

几乎每一天

无法停止或控制忧虑

一点也不

几天

超过半天

几乎每一天

情绪低落，沮丧或绝望

一点也不

几天

超过半天

几乎每一天

做事没有兴趣或乐趣

一点也不

几天

超过半天

几乎每一天

自从新冠肺炎危机开始以来，您多久出于以下原因大吃一次…

因为您沮丧或悲伤

几乎永远不会

很少

5269 有时
5270 经常
5271 几乎总是或总是
5272 作为帮助您应对的方式
5273 几乎永远不会
5274 很少
5275 有时
5276 经常
5277 几乎总是或总是
5278 安慰自己
5279 几乎永远不会
5280 很少
5281 有时
5282 经常
5283 几乎总是或总是
5284 因为您感到一文不值或自身不足
5285 几乎永远不会
5286 很少
5287 有时
5288 经常
5289 几乎总是或总是
5290 作为避免思考不愉快或分散自己注意力的一种方式
5291 几乎永远不会

很少
有时
经常
几乎总是或总是

表示您对以下陈述的赞同程度
我一生中有很多事情值得感谢
强烈反对
不同意
不太同意
既不同意也不反对
有些同意
同意
非常同意
如果我必须列出所有我感激的东西，那将是一个很长的清单
强烈反对
不同意
不太同意
既不同意也不反对
有些同意
同意
非常同意
我感谢各种各样的人
强烈反对
不同意
不太同意

既不同意也不反对
有些同意
同意
非常同意

请说明新冠肺炎危机对您的影响最大的方面：

自从新冠肺炎危机开始以来，以下人员的支持程度如何？
丈夫或其他重要的人
一点也不
一点点
适度
有点
非常
不适用（没有这样的人）
父母或法定监护人
一点也不
一点点
适度
有点
非常
不适用（没有这样的人）
孩子
一点也不
一点点
适度
有点
非常

不适用（没有这样的人）
兄弟姐妹
一点也不
一点点
适度
有点
非常
不适用（没有这样的人）
朋友
一点也不
一点点
适度
有点
非常
不适用（没有这样的人）
同事
一点也不
一点点
适度
有点
非常
不适用（没有这样的人）

自从新冠肺炎危机开始以来，以下人员的支持程度如何？
自从新冠肺炎危机开始以来，以下人员的支持程度如何？
丈夫或其他重要的人
一点也不
一点点

5374 适度
5375 有点
5376 非常
5377 不适用（没有这样的人）
5378 父母或法定监护人
5379 一点也不
5380 一点点
5381 适度
5382 有点
5383 非常
5384 不适用（没有这样的人）
5385 孩子
5386 一点也不
5387 一点点
5388 适度
5389 有点
5390 非常
5391 不适用（没有这样的人）
5392 兄弟姐妹
5393 一点也不
5394 一点点
5395 适度
5396 有点
5397 非常
5398 不适用（没有这样的人）
5399 朋友
5400 一点也不
5401 一点点

适度
有点
非常
不适用（没有这样的人）
同事
一点也不
一点点
适度
有点
非常
不适用（没有这样的人）

自从新冠肺炎危机开始以来...
您多久觉得自己缺乏陪伴？
几乎没有
一些时间
经常
您多久感到被疏忽？
几乎没有
一些时间
经常
您多久感到与他人隔离？
几乎没有
一些时间
经常

考虑您在过去 7 天内的经历，请指出您对以下每个陈述的赞同或反对程度

我不觉得自己属于我所谓的社区
非常同意
同意
有些同意
既不同意也不反对
不太同意
不同意
强烈反对

我有一些宝贵的东西可以给世界
非常同意
同意
有些同意
既不同意也不反对
不太同意
不同意
强烈反对

我与社区中的其他人感觉很亲密
非常同意
同意
有些同意
既不同意也不反对
不太同意
不同意
强烈反对

我无法理解世界上正在发生的事情

非常同意
同意
有些同意
既不同意也不反对
不太同意
不同意
强烈反对
我的社区是舒适之源
非常同意
同意
有些同意
既不同意也不反对
不太同意
不同意
强烈反对
我相信人很善良
非常同意
同意
有些同意
既不同意也不反对
不太同意
不同意
强烈反对

请根据最近 7 天来回答以下问题
我感到警惕或无法放松
一点也不

5481 一点点
5482 适度
5483 有点
5484 非常
5485 其他事情让我一直想起新冠肺炎
5486 一点也不
5487 一点点
5488 适度
5489 有点
5490 非常
5491 我知道我对新冠肺炎有很多感觉，但是我不去理会
5492 一点也不
5493 一点点
5494 适度
5495 有点
5496 非常
5497 我尽量不要考虑新冠肺炎
5498 一点也不
5499 一点点
5500 适度
5501 有点
5502 非常
5503 我无法集中精力
5504 一点也不
5505 一点点
5506 适度
5507 有点
5508 非常

请提供有关此调查的任何反馈：

您是怎么知道我们的？
(***list not transferred)

Arabic Pregistry Survey

في أي دولة تعيشين؟
(***list of all countries)

في أي مقاطعة/ولاية تعيشين؟

في أي مدينة تعيشين؟

هل انت حامل؟
نعم
لا

كم عدد أسابيع الحمل؟
(***list 5 to 43)

منذ متى أنجبتِ طفلك؟
(***list not transferred)

كم عمركِ؟
(***list 18 to 50)

آپ کس نسل ست تعلق رکھتی ہیں؟ (جوجو بھی لاگو ہوتا ہے اسے منتخب کریں))
أبيض/قوقازي
لاتيني/أسباني
آسيوي
آسيوي جنوبي
أسود
شرق أوسطي
سكان هاواي الأصليين أو جزء المحيط الهادئ الأخرى
هندي أمريكي أو من سكان ألاسكا الأصليين
أخرى/ معددة الأعراق

ما هي حالتكِ الزوجية؟
عزباء
متزوجة
أعيش مع والديّ
خلع يافتہ
مطلقة
أرملة

كم عدد الأشخاص الذين يعيشون في منزلك (بما في ذلك أنتِ)؟
(***list not transferred)

ما هو أعلى مؤهل دراسي حصلتِ عليه؟
لم أدخل المدرسة مطلقًا
المدرسة الابتدائية
المدرسة الإعدادية
تخرجت من المدرسة الثانوية أو أحمل دبلومة مكافئة.
دراسة جامعية
دبلومة أو درجة جامعية
ماجيستير
درجة احترافية
درجة دكتوراة

برجاء تحديد أي مما يلي ينطبق عليكِ خلال فترة وباء كوفيد-19:
عملتِ في مجال الرعاية الصحية في مستشفى أو عيادة
عملتِ في دار رعاية
كنتِ عامل أساسي (وفق تعريف الحكومة)
لا شيء من هذا
لا أعرف

هل لديك تأمين طبي من خلال حكومتك أو صاحب العمل أو أحد أفراد الأسرة؟
(***list not transferred)

کیا آپ نے سارز-کوو-2 (جو کرونا کاباعث بنتا ہے) کا معائنہ کرایا ہے؟ اگر ہاں تو کیا رزلٹ تھا؟

إيجابية، كنت مصابة بالفيروس
سلبية، لم أكن مصابة بالفيروس
نعم، ولكنني لا أعرف النتيجة بعد أو لم تكن النتيجة حاسمة
لا، لم أخضع للاختبار

هل شخصكِ أخصائي الرعاية الطبية (الطبيب أو الممرضة مثلًا) بالإصابة بفيروس كوفيد-19 اعتمادًا على الأعراض الظاهرة عليك فقط؟
(***list not transferred)

هل اختلطتِ بشخص يعاني من كوفيد-19 حاليًا أو سابقًا؟
(***list not transferred)

أي من الإجراءات التالية نفذتها خلال الأيام السبعة الماضية للحفاظ على سلامتك أو الآخرين من كوفيد-19؟ (حددي كل الاختيارات المنطبقة عليكِ)
ارتديتِ قناعًا للوجه
غسلتِ يديكِ بالماء والصابون أو استخدمتِ مطهّر يدين عدة مرات يوميًا
استخدمتِ مظهر أسطح في الأماكن المحيطة
خزّنتِ مطهر يدين أو مناديل مطهرة للأسطح
خزّنتِ الطعام أو الماء
ألغيتِ أو أجّلتِ سفرًا جويًا للعمل
ألغيتِ أو أجّلتِ سفرًا جويًا للمتعة
ألغيتِ أو أجّلتِ نشاطات في العمل أو المدرسة
ألغيتِ أو أجّلتِ نشاطات شخصية أو اجتماعية
تجنّبتِ التعامل مع أشخاص يحتمل أن يكونوا ذوي خطورة مرتفعة
تجنّبتِ الأماكن العامة أو التجمعات أو الحشود
تجنّبتِ تناول الطعام في المطاعم
عملتِ أو درستِ من المنزل
استشرتِ طبيبًا
ألغيتِ موعدًا مع طبيب
خزّنتِ الأدوية
صلّيتِ.

كم مرة تشاهدين أخبار حول كوفيد-19؟
مطلقًا
مرة/يوم >

مرة/يوميًا
مرة/يوم 2-4
مرة/يوم 5-8
مرة/يوم 9-16
مرة/يوم 16 <
كم مرة تتفقدين وسائل التواصل الاجتماعي حول كوفيد-19؟ (مثل واتساب وفيسبوك)
مطلقًا
مرة/يوم >
مرة/يوميًا
مرة/يوم 2-4
مرة/يوم 5-8
مرة/يوم 9-16
مرة/يوم 16 <
كم مرة تناقشين وباء كوفيد-19 في وسائل الإعلام الجماهيرية؟ (مثل مجموعات واتساب وتويتر)
مطلقًا
مرة/يوم >
مرة/يوميًا
مرة/يوم 2-4
مرة/يوم 5-8
مرة/يوم 9-16
مرة/يوم 16 <
كم مرة تناقشين وباء كوفيد-19 مع شخص آخر؟
مطلقًا
مرة/يوم >
مرة/يوميًا
مرة/يوم 2-4
مرة/يوم 5-8
مرة/يوم 9-16
مرة/يوم 16 <

ما مدى قلقكِ بشأن كوفيد-19؟
(***list not transferred)

:ما هي الأشياء التي تشعركِ بالقلق بشأن كوفيد-19؟ حددي كل الاختيارات المنطبقة عليكِ
إصابتي بوباء كوفيد-19 ونقل العدوى للمنزل
إصابة زوجي بوباء كوفيد-19 ونقل العدوى للمنزل
إصابة أفراد عائلتي/أصدقائي بوباء كوفيد-19
.تأثير وباء كوفيد-19 على وضعي المالي/الاقتصادي بصورة كبيرة (خسارة العمل مثلًا)
إصابة طفلي بوباء كوفيد-19
اضطراري لتغيير خطة الولادة بسبب وباء كوفيد-19
أن زوجي/معيلي لن يتمكن من التواجد معي أثناء الولادة بسبب كوفيد-19
عدم تمكن عائلتي من زيارتي أنا وطفلي بعد الولادة بسبب احتياطات منع انتشار كوفيد-19
إصابة أطفالي الآخرين بوباء كوفيد-19
عدم تمكن والديّ/جديّ من زيارة الطفل بسبب احتياطات منع انتشار كوفيد-19
عدم تمكني من إرضاع طفلي بسبب وباء كوفيد-19
عدم تمكني من تقديم رعاية مناسبة لأطفالي الآخرين
عدم تمكني من حضور جنازة أحد أفراد العائلة
تفويت/إلغاء مواعيد الطبيب
عدم تمكني من إقامة حفل أو مناسبة أخرى لطفلي مع العائلة أو الأصدقاء
أخرى

إلى أي مدى أثّر وباء كوفيد-19 بصورة سلبية على المناحي التالية في حياتك؟
النوم
على الإطلاق
قليلًا
بصورة متوسطة
كثيرًا
النظام الغذائي
النوم
على الإطلاق
قليلًا
بصورة متوسطة
كثيرًا
اللياقة
النوم
على الإطلاق

5679	قليلًا
5680	بصورة متوسطة
5681	كثيرًا
5682	العمل
5683	النوم
5684	على الإطلاق
5685	قليلًا
5686	بصورة متوسطة
5687	كثيرًا
5688	الماليات
5689	النوم
5690	على الإطلاق
5691	قليلًا
5692	بصورة متوسطة
5693	كثيرًا
5694	العائلة
5695	النوم
5696	على الإطلاق
5697	قليلًا
5698	بصورة متوسطة
5699	كثيرًا
5700	العلاقات
5701	النوم
5702	على الإطلاق
5703	قليلًا
5704	بصورة متوسطة
5705	كثيرًا
5706	تأدية الشعائر الدينية
5707	النوم
5708	على الإطلاق
5709	قليلًا
5710	بصورة متوسطة
5711	كثيرًا

الصفات الشخصية (كالصبر والثقة)

النوم

على الإطلاق

قليلًا

بصورة متوسطة

كثيرًا

كم مرة حدثت الأشياء التالية لكِ منذ ظهور وباء كوفيد-19؟

تم التعامل معكِ بكياسة أقل من الآخرين

كل يوم تقريبًا

مرة كل أسبوع على الأقل

بضع مرات شهريًا

مطلقًا

تلقيّتِ خدمة أسوأ من الآخرين في المطاعم أو المتاجر

كل يوم تقريبًا

مرة كل أسبوع على الأقل

بضع مرات شهريًا

مطلقًا

تصرّف أشخاص وكأنهم خائفون منك

كل يوم تقريبًا

مرة كل أسبوع على الأقل

بضع مرات شهريًا

مطلقًا

أطلق أشخاص عليك أسماءً أو تعرضتِ لإهانة

كل يوم تقريبًا

مرة كل أسبوع على الأقل

بضع مرات شهريًا

مطلقًا

تعرضّتِ للتهديد أو المضايقة

كل يوم تقريبًا

مرة كل أسبوع على الأقل

بضع مرات شهريًا

مطلقًا

تعرضّتِ لهجوم جسدي
كل يوم تقريبًا
مرة كل أسبوع على الأقل
بضع مرات شهريًا
مطلقًا

ما رأيك هو السبب الرئيسي لهذه التجارب؟ (اختر كل ما يمكن تطبيقه)
أصولك أو أصولك الوطنية
جنسك
عرقك أو عرقك
عمرك
دينك
طولك
وزنك
حملك
ميولك الجنسية
مستوى تعليمك أو دخلك
آخر

برجاء الإجابة على الأسئلة التالية بناءً على الأسبوعين السابقين
كم مرة شعرتِ بعدم قدرتكِ على التحكم في الأشياء المهمة في حياتك؟
مطلقًا
بصورة نادرة
حيانًا
بصورة متكررة
بصورة متتكرة للغاية
كم مرة شعرتِ بالثقة في قدرتكِ على التعامل مع المشاكل الشخصية؟
مطلقًا
بصورة نادرة
حيانًا
بصورة متكررة
بصورة متتكرة للغاية
كم مرة شعرتِ بأن الأشياء تسير حسب رغبتك؟

مطلقًا
بصورة نادرة
حيانًا
بصورة متكررة
بصورة متتكرة للغاية
كم مرة شعرتِ بتراكم الصعوبات لدرجة عدم تمكنك من التغلّب عليها؟
مطلقًا
بصورة نادرة
حيانًا
بصورة متكررة
بصورة متتكرة للغاية

كم مرة تعرضتِ للمشكلات التالية خلال الأسبوعين الماضيين؟
الشعور بالتوتّر أو العصبية أو الاضطراب
مطلقًا
عدة أيام
أكثر من نصف عدد الأيام
كل يوم تقريبًا
عدم التمكن من التوقف عن القلق أو التحكم فيه
مطلقًا
عدة أيام
أكثر من نصف عدد الأيام
كل يوم تقريبًا
الشعور بالإحباط أو الاكتئاب أو اليأس
مطلقًا
عدة أيام
أكثر من نصف عدد الأيام
كل يوم تقريبًا
عدم إيجاد متعة في أداء الأشياء
مطلقًا
عدة أيام
أكثر من نصف عدد الأيام
كل يوم تقريبًا

ما مقدار تناولك للطعام للأسباب التالية منذ بدء أزمة وباء كوفيد-19
لأنكِ مكتئبة أو حزينة
مطلقًا
نادرًا
أحيانًا
كثيرًا
دائمًا تقريبًا
كوسيلة للتكيّف
مطلقًا
نادرًا
أحيانًا
كثيرًا
دائمًا تقريبًا
كوسيلة لتعزية نفسك
مطلقًا
نادرًا
أحيانًا
كثيرًا
دائمًا تقريبًا
لأنكِ تشعرين بانعدام القيمة أو الكفاءة
مطلقًا
نادرًا
أحيانًا
كثيرًا
دائمًا تقريبًا
كطريقة لتجنب التفكير في شيء غير سار أو لإلهاء نفسك
مطلقًا
نادرًا
أحيانًا
كثيرًا
دائمًا تقريبًا

حددي مدى موافقتك على التصريحات التالية أو اختلافك معها
لدي الكثير من الأشياء التي تشعرني بالامتنان
أختلف بشدة
لا أوافق
لا أوافق إلى درجة ما
لا أوافق ولا أرفض
أوافق إلى درجة ما
أوافق
أوافق بشدة
سأكتب قائمة طويلة للغاية إن اضررت لكتابة قائمة بالأشياء التي تشعرني بالامتنان
أختلف بشدة
لا أوافق
لا أوافق إلى درجة ما
لا أوافق ولا أرفض
أوافق إلى درجة ما
أوافق
أوافق بشدة
أشعر بالشكر تجاه العديد من الأشخاص
أختلف بشدة
لا أوافق
لا أوافق إلى درجة ما
لا أوافق ولا أرفض
أوافق إلى درجة ما
أوافق
أوافق بشدة

ما مدى دعم الأشخاص التالي ذكرهم منذ بدء أزمة وباء كوفيد-19؟
الزوج أو الشريك
مطلقًا
قليلًا
بصورة متوسطة
إلى حد ما
بصورة كبيرة

5877	لا يوجد شخص كذلك
5878	الأبوين أو الوصي القانوني
5879	مطلقًا
5880	قليلًا
5881	بصورة متوسطة
5882	إلى حد ما
5883	بصورة كبيرة
5884	لا يوجد شخص كذلك
5885	الأطفال
5886	مطلقًا
5887	قليلًا
5888	بصورة متوسطة
5889	إلى حد ما
5890	بصورة كبيرة
5891	لا يوجد شخص كذلك
5892	الأقارب
5893	مطلقًا
5894	قليلًا
5895	بصورة متوسطة
5896	إلى حد ما
5897	بصورة كبيرة
5898	لا يوجد شخص كذلك
5899	الأصدقاء
5900	مطلقًا
5901	قليلًا
5902	بصورة متوسطة
5903	إلى حد ما
5904	بصورة كبيرة
5905	لا يوجد شخص كذلك
5906	زملاء العمل
5907	مطلقًا
5908	قليلًا
5909	بصورة متوسطة

إلى حد ما
بصورة كبيرة
لا يوجد شخص كذلك

ما مدى شعوركِ بالضيق تجاه الأشخاص التالي ذكرهم منذ بدء أزمة وباء كوفيد-19؟
الزوج أو الشريك
مطلقًا
قليلًا
بصورة متوسطة
إلى حد ما
بصورة كبيرة
لا يوجد شخص كذلك
الأبوين أو الوصي القانوني
مطلقًا
قليلًا
بصورة متوسطة
إلى حد ما
بصورة كبيرة
لا يوجد شخص كذلك
الأطفال
مطلقًا
قليلًا
بصورة متوسطة
إلى حد ما
بصورة كبيرة
لا يوجد شخص كذلك
الأقارب
مطلقًا
قليلًا
بصورة متوسطة
إلى حد ما
بصورة كبيرة
لا يوجد شخص كذلك

الأصدقاء
مطلقًا
قليلًا
بصورة متوسطة
إلى حد ما
بصورة كبيرة
لا يوجد شخص كذلك
زملاء العمل
مطلقًا
قليلًا
بصورة متوسطة
إلى حد ما
بصورة كبيرة
لا يوجد شخص كذلك

...منذ بدء أزمة وباء كوفيد-19
كم مرة تشعرين بانعدام الرفقة؟
عادة
بعض الوقت
مطلقًا
كم مرة تشعرين بالإهمال؟
عادة
بعض الوقت
مطلقًا
كم مرة تشعرين بانعزالك عن الآخرين؟
عادة
بعض الوقت
مطلقًا

برجاء تحديد مدى موافقتكِ أو اختلافك مع العبارات التالية اعتمادًا على تجربتكِ خلال الأيام السبعة الماضية
لا أشعر بانتمائي إلى كيان يمكن تسميته بالمجتمع
أوافق بشدة
أوافق

5976	أوافق إلى درجة ما
5977	لا أوافق ولا أرفض
5978	لا أوافق إلى درجة ما
5979	لا أوافق
5980	لا أوافق بشدة
5981	لدي شيء ثمين أقدمه للعالم
5982	أوافق بشدة
5983	أوافق
5984	أوافق إلى درجة ما
5985	لا أوافق ولا أرفض
5986	لا أوافق إلى درجة ما
5987	لا أوافق
5988	لا أوافق بشدة
5989	أشعر بأنني أقرب للأشخاص في مجتمعي
5990	أوافق بشدة
5991	أوافق
5992	أوافق إلى درجة ما
5993	لا أوافق ولا أرفض
5994	لا أوافق إلى درجة ما
5995	لا أوافق
5996	لا أوافق بشدة
5997	لا يمكنني إدراك ما يحدث في العالم
5998	أوافق بشدة
5999	أوافق
6000	أوافق إلى درجة ما
6001	لا أوافق ولا أرفض
6002	لا أوافق إلى درجة ما
6003	لا أوافق
6004	لا أوافق بشدة
6005	مجتمعي هو مصدر الراحة
6006	أوافق بشدة
6007	أوافق
6008	أوافق إلى درجة ما

لا أوافق ولا أرفض
لا أوافق إلى درجة ما
لا أوافق
لا أوافق بشدة
أعتقد بأن الناس لطفاء
أوافق بشدة
أوافق
أوافق إلى درجة ما
لا أوافق ولا أرفض
لا أوافق إلى درجة ما
لا أوافق
لا أوافق بشدة

برجاء الإجابة على الأسئلة التالية بناءً على الأيام السبعة الأخيرة
أشعر بالحذر أو الاحتراس
على الإطلاق
قليلًا
بصورة متوسطة
كثيرًا
بصورة مفرطة
تدفعني أشياء أخرى للتفكير بوباء كوفيد-19
على الإطلاق
قليلًا
بصورة متوسطة
كثيرًا
بصورة مفرطة
أدرك امتلاكي للكثير من المشاعر حول وباء كوفيد-19 ولكنني لا أتعامل معها
على الإطلاق
قليلًا
بصورة متوسطة
كثيرًا
بصورة مفرطة
أحاول ألا أفكر في وباء كوفيد-19

على الإطلاق
قليلًا
بصورة متوسطة
كثيرًا
بصورة مفرطة
أعاني من صعوبة في التركيز
على الإطلاق
قليلًا
بصورة متوسطة
كثيرًا
بصورة مفرطة

برجاء تقديم أية آراء حول استطلاع الرأي هذا:

كيف سمعتِ بنا؟
(***list not transferred)

Urdu Pregistry survey

آپ کس ملک میں رہتی ہیں؟
(***list of all countries)

آپ کس ریاست/صوبے میں رہتی ہیں؟

آپ کس شہر میں رہتی ہیں؟

آپ حاملہ ہیں
جی ہاں
نہیں

آپ کتنے ہفتوں سے حاملہ ہیں؟
(***list from 5 to 43)

آپ نے کتنی دیر پہلے پیدائش کی؟
(***list not transferred)

آپ کتنے سال کی ہیں؟
(***list from 18 to 50)

آپ کس ریس (ریس) کی سب سے زیادہ شناخت کرتے ہیں؟ (جو بھی لاگو ہوتا ہے اسے چیک کریں)
سفید / کاکیشین
لاطینی / هسپانوی
ایشین
جنوبی ایشین
سیاہ
مشرق وسطی
آبائی ہوائی یا دوسرا بحر الکاہل جزیرہ
امریکی ہندوستانی یا الاسکا آبائی
دیگر / ملٹیراس

آپ کی ازدواجی حیثیت کیا ہے؟
سنگل
شادی شدہ
ساتھی کے ساتھ رہنا
خلع یافتہ
طلاق ہوگئی
بیوہ

آپ کے گھر والے کتنے لوگ (اپنے آپ سمیت) رہتے ہیں؟
(***List not transferred)

آپ نے جو اعلی تعلیم حاصل کی ہے وہ کونسی ہے؟
کبھی اسکول نہیں پڑھا
ابتدائی اسکول
کچھ ہائی سکول
ہائی اسکول گریجویٹ یا عام مساوات ڈپلوما (جی ای ڈی)
کچھ کالج / یونیورسٹی
کالج ڈپلوما یا یونیورسٹی کی ڈگری
ماسٹرز کی ڈگری
پروفیشنل ڈگری
ڈاکٹریٹ کی ڈگری

:براہ کرم اس بات کی نشاندہی کریں کہ اگر کوویڈ-19 وبائی مرض کے دوران آپ کو
اسپتال یا کلینک میں ہیلتھ کیئر ورکر تھے
نرسنگ ہوم میں کام کیا
ایک لازمی / کلیدی کارکن تھے (جیسا کہ حکومت نے بیان کیا ہے)
ان میں سے کوئی نہیں
پتہ نہیں

کیا آپ کی حکومت ، اپنے آجر ، یا کنبہ کے ممبر کے ذریعہ آپ کا میڈیکل انشورنس ہے؟
(***list not transferred)

کیا آپ نے سارز-کوو-2 (جو کرونا کاباعث بنتا ہے) کا معائنہ کرایا ہے؟ اگر ہاں تو کیا رزلٹ تھا؟

مثبت ، مجھے وائرس تھا
منفی ، مجھے وائرس نہیں تھا
ہاں ، لیکن مجھے ابھی تک نتیجہ معلوم نہیں ہے یا نتیجہ نہیں نکلا تھا
نہیں ، میرا امتحان نہیں لیا گیا

کیا کسی صحت کی دیکھ بھال کے پیشہ ور (جیسے ، ڈاکٹر ، نرس) نے آپ کی علامات کی بنیاد پرآپ کو کرونا ہونے کی تشخیص کی ہے؟
(***list not transferred)

کیا آپ کسی ایسے فرد کے ساتھ رابطے میں رہے ہیں جس کو کرونا تھا؟
(***list not transferred)

اپنے اور دوسروں کو کرونا وائرس سے محفوظ رکھنے کے لئے آپ نے گذشتہ 7 دنوں میں مندرجہ ذیل میں سے کون سا کام انجام دیا ہے؟ (وہ سب سلیکٹ کریں جو مناسب ہے)
چہرہ ماسک پہنا تھا
اپنے ہاتھوں کو روزانہ کئی بار صابن سے دھویا یا ہینڈ سینیائزر استعمال کیا
اپنے گرد کی جگہوں کو جراثیم سے پاک کیا
ہینڈ سینیٹائزر یا جراثیم کش وائپس ذخیرہ کئے
ذخیرہ شدہ کھانا یا پانی
کام کے لئے منسوخ یا ملتوی ہوائی سفر
خوشی کے مواقع کے لئے ہوائی سفر منسوخ یا ملتوی کیا
منسوخ یا ملتوی کام یا اسکول کی سرگرمیاں
منسوخ یا ملتوی ذاتی یا سماجی سرگرمیاں
ایسے لوگوں سے رابطے سے گریز کریں جو زیادہ خطرہ ہوسکتے ہیں
عوامی مقامات ، اجتماعات یا ہجوم سے اجتناب کیا
ریستوراں میں کھانے سے پرہیز کیا
گھر میں کام کیا یا تعلیم حاصل کی
ڈاکٹر سے ملنے گئے
ڈاکٹر کی تقرری منسوخ کردی
ذخیرہ شدہ دوائیں
دعا کی

آپ کوویڈ- 19 سے متعلق خبروں کو کتنی بار دیکھتے ہیں؟
کبھی نہیں

<1 x / دن
1 x / دن
2-4 x / دن
5-8 x / دن
9-16 x / دن
> 16 x / دن
آپ کوویڈ-19 کے بارے میں کتنی بار سوشل میڈیا پر چیک کرتی ہیں؟ (جیسے واٹس ایپ ، فیس بک وغیرہ)
کبھی نہیں
<1 x / دن
1 x / دن
2-4 x / دن
5-8 x / دن
9-16 x / دن
> 16 x / دن
سوشل میڈیا پر آپ کتنی دفعہ کوویڈ-19 کےبارے میں کتنی بار بحث کرتے ہیں؟ (جیسے واٹس ایپ گروپ ، ٹویٹر)
کبھی نہیں
<1 x / دن
1 x / دن
2-4 x / دن
5-8 x / دن
9-16 x / دن
> 16 x / دن
آپ کوویڈ-19 کے بارے میں کسی اور شخص کے ساتھ کتنی بار گفتگو کرتی ہیں؟
کبھی نہیں
<1 x / دن
1 x / دن
2-4 x / دن
5-8 x / دن
9-16 x / دن
> 16 x / دن

آپ کوویڈ- 19 کے بارے میں کتنے پریشان ہیں؟
(***list not transferred)

کوویڈ-19 کے بارے میں کیا آپ کو سب سے زیادہ پریشان کرتا ہے؟ وہ سب سلیکٹ کریں جو مناسب ہے:
کہ مجھے کرونا وائرس ہو جائے گا اور میں انفیکشن کو گھر لاؤں گی
کہ میرے ساتھی یا دوستوں کو کرونا وائرس ہو جائے گا اور وہ انفیکشن گھر لے آئیں گے
کہ میرے کنبے کے ممبر / دوست کرونا وائرس سے متاثر ہوں گے۔
کہ کوویڈ-19 وبائی مرض سے میری معاشی صورتحال / مالی معاملات پر نمایاں اثر پڑے گا (مثال کے طور پر ، میری ملازمت سے محروم ہوجائیں)
کہ میرے غیر پیدا شدہ بچے کو کرونا وائرس ہو جائے گا۔
کرونا وائرس کی وجہ سے میرے ڈیلیوری کے پلان میں تبدیلی ہو جائے گی
کہ میرا شریک / معاون فرد کرونا کی وجہ سے ڈلیوری کے دوران میرے ساتھ نہیں رہ سکے گا
کہ کوویڈ-19کو روکنے کے اقدامات کی وجہ سے میرا خاندان ڈلیوری کے بعد مجھ اور بچے سے ملنے کے قابل نہیں ہوگا
کہ میرے دوسرے بچوں کو کرونا وائرس ہو جائے گا
کہ میرے والدین / دادا دادی کرونا وائرس کو روکنے کے اقدامات کی وجہ سے بچے سے ملنے کے قابل نہیں ہوں گے۔
کہ میں کرونا وائرس کی وجہ سے دودھ نہیں پلا سکوں گی۔
کہ میں اپنے دوسرے بچوں کے لئے مناسب طور پر بچوں کی دیکھ بھال نہیں کر پاوں گی
کہ میں فیملی ممبر کی آخری رسومات میں شرکت نہیں کروں گی
ڈاکٹر کی ملاقات منسوخ/ملتوی ہو رہی ہے
کہ میں کنبہ یا دوستوں کے ساتھ بیبی شاور یا بچے کی پیدائش کے دوسرے جشن نہیں منا سکوں گی۔
دیگر

کرونا وائرس نے آپ کی زندگی کے مندرجہ ذیل شعبوں پر کس حد تک منفی اثر ڈالا ہے؟
سونا
بلکل بھی نہیں
تھوڑا سا
اعتدال سے
بہت زیادہ
غذا
بلکل بھی نہیں
تھوڑا سا
اعتدال سے
بہت زیادہ
صحت
بلکل بھی نہیں
تھوڑا سا

6223	اعتدال سے
6224	بہت زیادہ
6225	کام
6226	بلکل بھی نہیں
6227	تھوڑا سا
6228	اعتدال سے
6229	بہت زیادہ
6230	مالی حیثیت
6231	بلکل بھی نہیں
6232	تھوڑا سا
6233	اعتدال سے
6234	بہت زیادہ
6235	کنبہ
6236	بلکل بھی نہیں
6237	تھوڑا سا
6238	اعتدال سے
6239	بہت زیادہ
6240	تعلقات
6241	بلکل بھی نہیں
6242	تھوڑا سا
6243	اعتدال سے
6244	بہت زیادہ
6245	مذہبی عمل
6246	بلکل بھی نہیں
6247	تھوڑا سا
6248	اعتدال سے
6249	بہت زیادہ
6250	کریکٹر (جیسے ، صبر ، اعتماد)
6251	بلکل بھی نہیں
6252	تھوڑا سا
6253	اعتدال سے
6254	بہت زیادہ
6255	

چونکہ کوویڈ 19 وبائی بیماری کا آغاز ہوا ، مندرجہ ذیل میں سے کسی کو آپ کے ساتھ کتنی بار ہوا ہے؟
دوسرے لوگوں کی نسبت آپ کے ساتھ سلوک کم رہا
تقریبا ہر دن
کم از کم ہفتے میں ایک بار
ایک مہینہ میں کچھ بار
کبھی نہیں
آپ کو ریستوران یا اسٹورز میں دوسرے لوگوں کے مقابلے میں کم خدمت ملی
تقریبا ہر دن
کم از کم ہفتے میں ایک بار
ایک مہینہ میں کچھ بار
کبھی نہیں
لوگوں نے ایسا برتاؤ کیا جیسے وہ آپ سے ڈرتے ہیں
تقریبا ہر دن
کم از کم ہفتے میں ایک بار
ایک مہینہ میں کچھ بار
کبھی نہیں
آپ کو نام سے پکارا گیا یا توہین کیا گیا
تقریبا ہر دن
کم از کم ہفتے میں ایک بار
ایک مہینہ میں کچھ بار
کبھی نہیں
آپ کو دھمکی دی گئی یا پریشان کیا گیا
تقریبا ہر دن
کم از کم ہفتے میں ایک بار
ایک مہینہ میں کچھ بار
کبھی نہیں
آپ پر جسمانی حملہ کیا گیا
تقریبا ہر دن
کم از کم ہفتے میں ایک بار
ایک مہینہ میں کچھ بار
کبھی نہیں

آپ کے خیال میں ان تجربات کی سب سے بڑی وجہ کیا ہے؟ (جو بھی لاگو ہوتا ہے اس کا انتخاب کریں)

6289 آپ کے آباؤ اجداد یا قومی اصل
6290 آپ کی صنف
6291 آپ کی نسل یا نسل
6292 آپ کی عمر
6293 آپ کا مذہب
6294 آپ کا قد
6295 آپ کا وزن
6296 آپ کا حمل
6297 آپ کا جنسی رجحان
6298 آپ کی تعلیم یا آمدنی کی سطح
6299 دیگر
6300

6301 براہ کرم گذشتہ 2 ہفتوں کی بنیاد پر درج ذیل سوالات کے جوابات دیں۔
6302 آپ نے کتنی بار محسوس کیا ہے کہ آپ اپنی زندگی کی اہم چیزوں پر قابو نہیں پاسکتے ہیں؟
6303

6304 کبھی نہیں
6305 تقریبا کبھی نہیں
6306 کبھی کبھی
6307 کافی بار
6308 بہت اکر
6309 آپ نے اپنی ذاتی پریشانیوں سے نمٹنے کی اپنی صلاحیت کے بارے میں کتنی بار اعتماد محسوس کیا ہے؟
6310 کبھی نہیں
6311 تقریبا کبھی نہیں
6312 کبھی کبھی
6313 کافی بار
6314 بہت اکر
6315 آپ نے کتنی بار محسوس کیا ہے کہ معاملات آپ کے راستے پر چل رہے ہیں؟
6316 کبھی نہیں
6317 تقریبا کبھی نہیں
6318 کبھی کبھی
6319 کافی بار
6320 بہت اکر
6321 آپ نے کتنی بار محسوس کیا ہے کہ مشکلات اتنی زیادہ ہو رہی ہیں کہ آپ ان پر قابو نہیں پا سکتیں؟

6322 کبھی نہیں
6323 تقریبا کبھی نہیں
6324 کبھی کبھی
6325 کافی بار
6326 بہت اکر
6327
6328 آپ پچھلے 2 ہفتوں کے دوران رج ذیل مسائل سے کتنی بار پریشان ہو چکے ہیں؟
6329 گھبراہٹ ، پریشان یا کنارے پر محسوس کرنا
6330 بلکل بھی نہیں
6331 کئی دن
6332 آدھے دن سے زیادہ
6333 تقریبا روزانہ
6334 پریشانی کو روکنے یا کنٹرول کرنے کے قابل نہیں ہونا
6335 بلکل بھی نہیں
6336 کئی دن
6337 آدھے دن سے زیادہ
6338 تقریبا روزانہ
6339 افسردہ ، افسردہ یا مایوس
6340 بلکل بھی نہیں
6341 کئی دن
6342 آدھے دن سے زیادہ
6343 تقریبا روزانہ
6344 کام کرنے میں تھوڑی دلچسپی یا خوشی
6345 بلکل بھی نہیں
6346 کئی دن
6347 آدھے دن سے زیادہ
6348 تقریبا روزانہ
6349
6350 ... جب سے آپ کوویڈ 19 بحران شروع ہوا ہے ، آپ کتنی بار کھاتی ہیں
6351 جب آپ افسردہ یا غمزدہ ہوں
6352 تقریبا کبھی یا کبھی نہیں
6353 شاذ و نادر ہی
6354 کبھی کبھی

6355 اکثر
6356 تقریبا ہمیشہ یا ہمیشہ
6357 آپ کو مقابلہ کرنے میں مدد کرنے کے ایک طریقہ کے طور پر
6358 تقریبا کبھی یا کبھی نہیں
6359 شاذ و نادر ہی
6360 کبھی کبھی
6361 اکثر
6362 تقریبا ہمیشہ یا ہمیشہ
6363 اپنے آپ کو تسلی دینے کا ایک طریقہ
6364 تقریبا کبھی یا کبھی نہیں
6365 شاذ و نادر ہی
6366 کبھی کبھی
6367 اکثر
6368 تقریبا ہمیشہ یا ہمیشہ
6369 کیونکہ آپ خود کو ناکارہ یا ناکافی محسوس کرتے ہیں
6370 تقریبا کبھی یا کبھی نہیں
6371 شاذ و نادر ہی
6372 کبھی کبھی
6373 اکثر
6374 تقریبا ہمیشہ یا ہمیشہ
6375 کسی ناخوشگوار چیز کے بارے میں سوچنے سے گریز کرنے یا اپنے آپ کو ہٹانے کے راستے کے طور پر
6376 تقریبا کبھی یا کبھی نہیں
6377 شاذ و نادر ہی
6378 کبھی کبھی
6379 اکثر
6380 تقریبا ہمیشہ یا ہمیشہ
6381
6382 بتائیں کہ آپ مندرجہ ذیل بیانات سے کتنا متفق ہیں
6383 میں زندگی میں بہت کچھ کروں جس کی میں شکر گذار رہوں
6384 بہت زیادہ اختلاف
6385 متفق نہیں
6386 کسی حد تک متفق نہیں ہوں
6387 نہ ہی متفق ہیں اور نہ ہی متفق ہیں

کسی حد تک راضی ہوں
متفق ہوں
بہت زیادہ اتفاق
مجھے اگر مجھے ایسی لسٹ بنانے کا موقع ملتا ک جن کی میں شکر گزار ہوں ، تو یہ بہت لمبی فہرست ہوتی
بہت زیادہ اختلاف
متفق نہیں
کسی حد تک متفق نہیں ہوں
نہ ہی متفق ہیں اور نہ ہی متفق ہیں
کسی حد تک راضی ہوں
متفق ہوں
بہت زیادہ اتفاق
میں بہت سارے لوگوں کا مشکور ہوں
بہت زیادہ اختلاف
متفق نہیں
کسی حد تک متفق نہیں ہوں
نہ ہی متفق ہیں اور نہ ہی متفق ہیں
کسی حد تک راضی ہوں
متفق ہوں
بہت زیادہ اتفاق

:براہ کرم وضاحت کریں کہ کوویڈ-19 بحران نے آپ پر سب سے زیادہ اثر کس طرح کیا ہے

چونکہ کوویڈ 19 بحران شروع ہوا ، مندرجہ ذیل افراد کتنے معاون ہیں؟
شوہر یا دوسرے اہم لوگ
بلکل بھی نہیں
تھوڑا سا
اعتدال سے
بہت تھوڑا سا
انتہائی
N / A (ایسا کوئی شخص نہیں ہے)
بچے
بلکل بھی نہیں
تھوڑا سا

6421 اعتدال سے
6422 بہت تھوڑا سا
6423 انتہائی
6424 N / A (ایسا کوئی شخص نہیں ہے)
6425 بہن بھائی
6426 بلکل بھی نہیں
6427 تھوڑا سا
6428 اعتدال سے
6429 بہت تھوڑا سا
6430 انتہائی
6431 N / A (ایسا کوئی شخص نہیں ہے)
6432 دوست
6433 بلکل بھی نہیں
6434 تھوڑا سا
6435 اعتدال سے
6436 بہت تھوڑا سا
6437 انتہائی
6438 N / A (ایسا کوئی شخص نہیں ہے)
6439 ساتھی کارکنان
6440 بلکل بھی نہیں
6441 تھوڑا سا
6442 اعتدال سے
6443 بہت تھوڑا سا
6444 انتہائی
6445 N / A (ایسا کوئی شخص نہیں ہے)
6446
6447 چونکہ کوویڈ 19 بحران شروع ہوا ، مندرجہ ذیل لوگوں کے ذریعہ آپ کو کتنا دباؤ محسوس ہوتا ہے؟
6448 شوہر یا دوسرے اہم لوگ
6449 بلکل بھی نہیں
6450 تھوڑا سا
6451 اعتدال سے
6452 بہت تھوڑا سا
6453 انتہائی

(ایسا کوئی شخص نہیں ہے) N / A
بچے
بلکل بھی نہیں
تھوڑا سا
اعتدال سے
بہت تھوڑا سا
انتہائی
(ایسا کوئی شخص نہیں ہے) N / A
بہن بھائی
بلکل بھی نہیں
تھوڑا سا
اعتدال سے
بہت تھوڑا سا
انتہائی
(ایسا کوئی شخص نہیں ہے) N / A
دوست
بلکل بھی نہیں
تھوڑا سا
اعتدال سے
بہت تھوڑا سا
انتہائی
(ایسا کوئی شخص نہیں ہے) N / A
ساتھی کارکنان
بلکل بھی نہیں
تھوڑا سا
اعتدال سے
بہت تھوڑا سا
انتہائی
(ایسا کوئی شخص نہیں ہے) N / A

چونکہ کوویڈ- 19 بحران شروع ہوا
آپ کتنی بار محسوس کرتے ہیں کہ آپ کی صحبت کا فقدان ہے؟
شاید ہی کبھی

کچھ وقت
اکثر
آپ کو کتنی بار چھوڑا ہوا محسوس ہوتا ہے؟
شاید ہی کبھی
کچھ وقت
اکثر
آپ کتنی بار دوسروں سے الگ تھلگ محسوس کرتے ہو؟
شاید ہی کبھی
کچھ وقت
اکثر

گذشتہ 7 دنوں میں اپنے تجربے کے بارے میں سوچتے ہوئے ، براہ کرم اس بات کی نشاندہی کریں کہ آپ درج ذیل میں سے ہر ایک کے بیان سے کس حد تک متفق یا متفق نہیں ہیں۔
مجھے نہیں لگتا کہ میں کسی بھی کمیونٹی یا ایسی کہلانے والی کسی بھی چیز سے تعلق رکھتی ہوں
بہت زیادہ اتفاق
متفق ہوں
کسی حد تک راضی ہوں
نہ ہی متفق ہیں اور نہ ہی متفق ہیں
کسی حد تک متفق نہیں ہوں
متفق نہیں
بہت زیادہ اختلاف
میرے پاس دنیا کو دینے کے لئے کچھ قیمتی چیز ہے
بہت زیادہ اتفاق
متفق ہوں
کسی حد تک راضی ہوں
نہ ہی متفق ہیں اور نہ ہی متفق ہیں
کسی حد تک متفق نہیں ہوں
متفق نہیں
بہت زیادہ اختلاف
میں اپنی برادری کے دوسرے لوگوں سے قربت محسوس کرتی ہوں
بہت زیادہ اتفاق
متفق ہوں
کسی حد تک راضی ہوں

نہ ہی متفق ہیں اور نہ ہی متفق ہیں
کسی حد تک متفق نہیں ہوں
متفق نہیں
بہت زیادہ اختلاف
میں دنیا میں کیا ہو رہا ہے اس کا احساس نہیں کرسکتی
بہت زیادہ اتفاق
متفق ہوں
کسی حد تک راضی ہوں
نہ ہی متفق ہیں اور نہ ہی متفق ہیں
کسی حد تک متفق نہیں ہوں
متفق نہیں
بہت زیادہ اختلاف
میری برادری سکون کا باعث ہے
بہت زیادہ اتفاق
متفق ہوں
کسی حد تک راضی ہوں
نہ ہی متفق ہیں اور نہ ہی متفق ہیں
کسی حد تک متفق نہیں ہوں
متفق نہیں
بہت زیادہ اختلاف
مجھے یقین ہے کہ لوگ مہربان ہیں
بہت زیادہ اتفاق
متفق ہوں
کسی حد تک راضی ہوں
نہ ہی متفق ہیں اور نہ ہی متفق ہیں
کسی حد تک متفق نہیں ہوں
متفق نہیں
بہت زیادہ اختلاف

براہ کرم گذشتہ 7 دنوں کی بنیاد پر درج ذیل سوالات کے جوابات دیں
مجھے باحفاظت یا محفوظ محسوس ہوتا ہے
بلکل بھی نہیں
تھوڑا سا

اعتدال سے
بہت تھوڑا سا
انتہائی
دوسری چیزیں مجھےکرونا وائرس کے بارے میں سوچنے پر مجبور کرتی ہیں
بلکل بھی نہیں
تھوڑا سا
اعتدال سے
بہت تھوڑا سا
انتہائی
میں جانتی ہوں کہ کرونا وائرس کے بارے میں میرے بہت زیادہ جذبات ہیں ، لیکن میں ان کی پرواہ نہیں کرتی ہوں۔
بلکل بھی نہیں
تھوڑا سا
اعتدال سے
بہت تھوڑا سا
انتہائی
میں کرونا وائرس کے بارے میں کم سوچنے کی کوشش کرتی ہوں
بلکل بھی نہیں
تھوڑا سا
اعتدال سے
بہت تھوڑا سا
انتہائی
مجھے توجہ دینے میں دشواری ہے
بلکل بھی نہیں
تھوڑا سا
اعتدال سے
بہت تھوڑا سا
انتہائی

:براہ کرم اس سروے کے بارے میں کوئی تاثرات فراہم کریں

ہمارے بارے میں آپ نے کیسے سنا؟
(***list not transferred)
